# Measuring the availability of human resources for health and its relationship to universal health coverage for 204 countries and territories from 1990 to 2019: a systematic analysis for the Global Burden of Disease Study 2019

**DOI:** 10.1016/S0140-6736(22)00532-3

**Published:** 2022-06-04

**Authors:** GBD 2019 Human Resources for Health Collaborators, Annie Haakenstad, Caleb Mackay Salpeter Irvine, Megan Knight, Corinne Bintz, Aleksandr Y Aravkin, Peng Zheng, Vin Gupta, Michael R M Abrigo, Abdelrahman I Abushouk, Oladimeji M Adebayo, Gina Agarwal, Fares Alahdab, Ziyad Al-Aly, Khurshid Alam, Turki M Alanzi, Jacqueline Elizabeth Alcalde-Rabanal, Vahid Alipour, Nelson Alvis-Guzman, Arianna Maever L Amit, Catalina Liliana Andrei, Tudorel Andrei, Carl Abelardo T Antonio, Jalal Arabloo, Olatunde Aremu, Martin Amogre Ayanore, Maciej Banach, Till Winfried Bärnighausen, Celine M Barthelemy, Mohsen Bayati, Habib Benzian, Adam E Berman, Kelly Bienhoff, Ali Bijani, Boris Bikbov, Antonio Biondi, Archith Boloor, Reinhard Busse, Zahid A Butt, Luis Alberto Cámera, Ismael R Campos-Nonato, Rosario Cárdenas, Felix Carvalho, Collins Chansa, Soosanna Kumary Chattu, Vijay Kumar Chattu, Dinh-Toi Chu, Xiaochen Dai, Lalit Dandona, Rakhi Dandona, William James Dangel, Ahmad Daryani, Jan-Walter De Neve, Meghnath Dhimal, Isaac Oluwafemi Dipeolu, Shirin Djalalinia, Hoa Thi Do, Chirag P Doshi, Leila Doshmangir, Elham Ehsani-Chimeh, Maha El Tantawi, Eduarda Fernandes, Florian Fischer, Nataliya A Foigt, Artem Alekseevich Fomenkov, Masoud Foroutan, Takeshi Fukumoto, Nancy Fullman, Mohamed M Gad, Keyghobad Ghadiri, Mansour Ghafourifard, Ahmad Ghashghaee, Thomas Glucksman, Houman Goudarzi, Rajat Das Gupta, Randah R Hamadeh, Samer Hamidi, Josep Maria Haro, Edris Hasanpoor, Simon I Hay, Mohamed I Hegazy, Behzad Heibati, Nathaniel J Henry, Michael K Hole, Naznin Hossain, Mowafa Househ, Olayinka Stephen Ilesanmi, Mohammad-Hasan Imani-Nasab, Seyed Sina Naghibi Irvani, Sheikh Mohammed Shariful Islam, Mohammad Ali Jahani, Ankur Joshi, Rohollah Kalhor, Gbenga A Kayode, Nauman Khalid, Khaled Khatab, Adnan Kisa, Sonali Kochhar, Kewal Krishan, Barthelemy Kuate Defo, Dharmesh Kumar Lal, Faris Hasan Lami, Anders O Larsson, Janet L Leasher, Kate E LeGrand, Lee-Ling Lim, Narayan B Mahotra, Azeem Majeed, Afshin Maleki, Narayana Manjunatha, Benjamin Ballard Massenburg, Tomislav Mestrovic, GK Mini, Andreea Mirica, Erkin M Mirrakhimov, Yousef Mohammad, Shafiu Mohammed, Ali H Mokdad, Shane Douglas Morrison, Mohsen Naghavi, Duduzile Edith Ndwandwe, Ionut Negoi, Ruxandra Irina Negoi, Josephine W Ngunjiri, Cuong Tat Nguyen, Yeshambel T Nigatu, Obinna E Onwujekwe, Doris V Ortega-Altamirano, Nikita Otstavnov, Stanislav S Otstavnov, Mayowa O Owolabi, Abhijit P Pakhare, Veincent Christian Filipino Pepito, Norberto Perico, Hai Quang Pham, David M Pigott, Khem Narayan Pokhrel, Mohammad Rabiee, Navid Rabiee, Vafa Rahimi-Movaghar, David Laith Rawaf, Salman Rawaf, Lal Rawal, Giuseppe Remuzzi, Andre M N Renzaho, Serge Resnikoff, Nima Rezaei, Aziz Rezapour, Jennifer Rickard, Leonardo Roever, Maitreyi Sahu, Abdallah M Samy, Juan Sanabria, Milena M Santric-Milicevic, Sivan Yegnanarayana Iyer Saraswathy, Soraya Seedat, Subramanian Senthilkumaran, Edson Serván-Mori, Masood Ali Shaikh, Aziz Sheikh, Diego Augusto Santos Silva, Caroline Stein, Dan J Stein, Mariya Vladimirovna Titova, Stephanie M Topp, Marcos Roberto Tovani-Palone, Saif Ullah, Bhaskaran Unnikrishnan, Marco Vacante, Pascual R Valdez, Tommi Juhani Vasankari, Narayanaswamy Venketasubramanian, Vasily Vlassov, Theo Vos, Jamal Akeem Yearwood, Naohiro Yonemoto, Mustafa Z Younis, Chuanhua Yu, Siddhesh Zadey, Sojib Bin Zaman, Taddese Alemu Zerfu, Zhi-Jiang Zhang, Arash Ziapour, Sanjay Zodpey, Stephen S Lim, Christopher J L Murray, Rafael Lozano

## Abstract

**Background:**

Human resources for health (HRH) include a range of occupations that aim to promote or improve human health. The UN Sustainable Development Goals (SDGs) and the WHO Health Workforce 2030 strategy have drawn attention to the importance of HRH for achieving policy priorities such as universal health coverage (UHC). Although previous research has found substantial global disparities in HRH, the absence of comparable cross-national estimates of existing workforces has hindered efforts to quantify workforce requirements to meet health system goals. We aimed to use comparable and standardised data sources to estimate HRH densities globally, and to examine the relationship between a subset of HRH cadres and UHC effective coverage performance.

**Methods:**

Through the International Labour Organization and Global Health Data Exchange databases, we identified 1404 country-years of data from labour force surveys and 69 country-years of census data, with detailed microdata on health-related employment. From the WHO National Health Workforce Accounts, we identified 2950 country-years of data. We mapped data from all occupational coding systems to the International Standard Classification of Occupations 1988 (ISCO-88), allowing for standardised estimation of densities for 16 categories of health workers across the full time series. Using data from 1990 to 2019 for 196 of 204 countries and territories, covering seven Global Burden of Diseases, Injuries, and Risk Factors Study (GBD) super-regions and 21 regions, we applied spatiotemporal Gaussian process regression (ST-GPR) to model HRH densities from 1990 to 2019 for all countries and territories. We used stochastic frontier meta-regression to model the relationship between the UHC effective coverage index and densities for the four categories of health workers enumerated in SDG indicator 3.c.1 pertaining to HRH: physicians, nurses and midwives, dentistry personnel, and pharmaceutical personnel. We identified minimum workforce density thresholds required to meet a specified target of 80 out of 100 on the UHC effective coverage index, and quantified national shortages with respect to those minimum thresholds.

**Findings:**

We estimated that, in 2019, the world had 104·0 million (95% uncertainty interval 83·5–128·0) health workers, including 12·8 million (9·7–16·6) physicians, 29·8 million (23·3–37·7) nurses and midwives, 4·6 million (3·6–6·0) dentistry personnel, and 5·2 million (4·0–6·7) pharmaceutical personnel. We calculated a global physician density of 16·7 (12·6–21·6) per 10 000 population, and a nurse and midwife density of 38·6 (30·1–48·8) per 10 000 population. We found the GBD super-regions of sub-Saharan Africa, south Asia, and north Africa and the Middle East had the lowest HRH densities. To reach 80 out of 100 on the UHC effective coverage index, we estimated that, per 10 000 population, at least 20·7 physicians, 70·6 nurses and midwives, 8·2 dentistry personnel, and 9·4 pharmaceutical personnel would be needed. In total, the 2019 national health workforces fell short of these minimum thresholds by 6·4 million physicians, 30·6 million nurses and midwives, 3·3 million dentistry personnel, and 2·9 million pharmaceutical personnel.

**Interpretation:**

Considerable expansion of the world's health workforce is needed to achieve high levels of UHC effective coverage. The largest shortages are in low-income settings, highlighting the need for increased financing and coordination to train, employ, and retain human resources in the health sector. Actual HRH shortages might be larger than estimated because minimum thresholds for each cadre of health workers are benchmarked on health systems that most efficiently translate human resources into UHC attainment.

**Funding:**

Bill & Melinda Gates Foundation.

**Research in context**

**Evidence before this study**

Monitoring health worker densities and distribution is crucial to health-systems analysis and planning at both national and international levels. Much existing research has assessed the size, composition, and efficacy of health-care workforces in individual countries and regions. Although useful, the lack of comparability of these studies impedes assessments of relative health workforce levels across countries and territories. WHO's Global Health Observatory compiles data on workforce densities by professional cadre that are mostly obtained from national statistical organisations and ministry of health repositories. These sources do not report data for all years and use a variety of data collection methods and standards, limiting the comparability of data across locations over time. WHO has issued two sets of minimum human resources for health (HRH) thresholds. The 2006 World Health Report threshold of 22·8 skilled health workers per 10 000 population was based on the mean level of physicians, nurses, and midwives observed across countries achieving a skilled birth attendance of 80%. In 2016, WHO used Global Health Observatory data to produce an updated threshold for the same aggregate cadres. This threshold was based on skilled health worker densities in countries with a median observed achievement on an index composed of 12 indicators of the UN Sustainable Development Goals (SDGs). Based on this method, WHO calculated 44·5 physicians, nurses, and midwives per 10 000 population as a new minimum density threshold. WHO has also issued two reports on nursing and midwifery, which include estimates of the scale of the global shortage of nurses and midwives: the *State of the World's Nursing 2020* report and the *State of the World's Midwifery 2021* report.

**Added value of this study**

This analysis used standard methods and comparable data to quantify densities for 16 HRH cadres in 204 countries and territories for every year from 1990 to 2019. We then used the time series of HRH densities and the universal health coverage (UHC) effective coverage index to calculate, for the first time, health workforce minimum thresholds for each of the four health worker cadres identified in SDG indicator 3.c.1 and related shortages. These thresholds represent the minimum levels of HRH required to achieve target levels of UHC, if countries are efficient in translating human resources into UHC attainment. This new threshold approach is a compromise between the ongoing demand from policy communities for standardised workforce benchmarks and the reality that considerable variation in skill mix undermines the utility of inflexible global targets. Rather than identify ideal levels of HRH intended to pertain to all contexts, our density thresholds specifically represent the minimum levels of human resources needed to achieve a UHC performance goal of 80 out of 100 in UHC effective coverage. This reflects a high performance level that still falls within the spectrum of observed attainment among a diverse set of countries examined, making the corresponding thresholds broadly useful for health-system strengthening efforts. Furthermore, this novel approach to estimating the frontier of UHC effective coverage at a given level of HRH might also be useful in other health-system performance or efficiency analyses.

**Implications of all the available evidence**

HRH densities and disparities are strongly related to sociodemographic development. In 2019, 168 of 204 countries and territories had workforce gaps in one or more of the four cadres of HRH compared to what is needed to achieve a UHC effective coverage score of 80 out of 100. This is likely to be an underestimate of actual shortages given that the threshold calculation assumes maximally efficient translation of health workforces into UHC attainment, and there is considerable variation in practice. Even with this potential underestimation, expansion of health-care workforces is needed in many locations to achieve improved UHC effective coverage.

## Introduction

Human resources for health (HRH) are crucial to health-system functioning,^1^, ^2^, ^3^, ^4^ but previous studies have found considerable differences in HRH densities across countries.^5^, ^6^, ^7^, ^8^, ^9^, ^10^ The importance of addressing workforce gaps is underscored by studies linking HRH to population-level health outcomes^11^, ^12^ and research suggesting that investing in health workforces promotes economic growth.^13^ The COVID-19 pandemic has also revealed the importance of health workers for an effective pandemic response.^14^ Health worker density and distribution is indicator 3.c.1 of the UN Sustainable Development Goals (SDGs), helping to track the “recruitment, development, training, and retention of health workforce[s]”.^15^ Additionally, WHO has outlined an ambitious agenda for expanding and improving the quality of health workforces by 2030.^16^

Despite this attention, comprehensive national health workforce estimates based on comparable data and standard methods are not available. Numerous studies of health workforces have been done at the national, regional, and subnational levels,^17^, ^18^, ^19^, ^20^, ^21^, ^22^, ^23^, ^24^, ^25^ but these do not present a comprehensive assessment of all or most countries and territories. WHO's Global Health Observatory releases workforce density data for various countries and cadres, including physicians, nurses and midwives, dentists, pharmacists, and other groupings.^26^ Gaps in the data and lack of standardisation across sources, however, restrict the comparability of these numbers.^27^, ^28^ The Global Health Observatory acts as a repository and WHO density numbers are based on an array of data sources that might differ in their definitions of HRH cadres across contexts. Additionally, many WHO sources are country reports, which might not capture health workers employed in the private sector and might rely on payroll lists from different providers that count the same health worker more than once.^29^

Estimates of how many health workers are needed to achieve health-system goals such as universal health coverage (UHC) have been affected by these data limitations as well as by other methodological choices.^30^ In 2006, WHO based minimum thresholds of skilled health workers (physicians, nurses, and midwives) on the mean workforce levels observed in countries achieving a skilled birth attendance of 80%.^6^ In 2016, WHO adopted a new method that quantified how many health workers are needed to achieve a median performance on an SDG index composed of 12 tracer indicators.^31^ WHO's aggregate density thresholds might not be sufficiently specific in that they do not identify nursing and midwifery needs separately from those of physicians, and they do not identify additional cadres that might contribute to the achievement of health outcomes. They also imply a 1:1 substitutability between health workers in different cadres that might not always be accurate. Finally, the WHO thresholds are estimated with respect to crude coverage indicators that might not reflect health service quality, and could pertain to factors beyond the direct activities of health systems (eg, the prevalence of tobacco smoking).^32^

The present study had two aims: to use comparable and standardised data sources to estimate levels of HRH for 16 health worker cadres across 204 countries and territories for a complete time series from 1990 to 2019, and to examine the relationship between a subset of HRH cadres and UHC effective coverage performance. Our study focused on the core cadres highlighted in SDG indicator 3.c.1 metadata: physicians, nurses and midwives, dentistry personnel, and pharmaceutical personnel. Quantification of the densities and minimum thresholds of HRH required for UHC effective coverage allows us to estimate where there are health workforce shortages that should be addressed.

This manuscript was produced as part of the Global Burden of Diseases, Injuries, and Risk Factors Study (GBD) Collaborator Network and in accordance with the GBD Protocol.

## Methods

### Overview

The main steps of the estimation process are presented below. Substantially more details and links to the codes and sources are available in appendix 1 (section 3). Some of these methods have been described in previous GBD publications.^32^, ^33^ Analyses were done with R (version 3.4.4), Python (version 2.7.14), or Stata (version 13.1), and figures were generated with R (version 3.4.4). This study fully adheres to the Guidelines for Accurate and Transparent Health Estimates Reporting (GATHER) statement.^34^ This study used the GBD 2019 location hierarchy covering seven GBD super-regions,^35^ 21 regions, and 204 countries and territories, along with corresponding estimates of population sizes.^36^ The study estimated densities of employed health workers in 16 HRH cadres for all of these locations from 1990 to 2019 inclusive.

### Data sources

Input data include data from WHO's Global Health Observatory and representative cross-sectional surveys and censuses that asked working-age respondents (defined as those aged 15–69 years) to self-report their employment status and current occupation. Surveys and censuses were restricted to those that coded responses to a level of detail that matched the granularity of the International Standard Classification of Occupations 1988 (ISCO-88) three-digit or four-digit codes. All survey and census sources were identified through the Global Health Data Exchange and International Labour Organization databases, and extracted if individual-level survey microdata were available. Most screened sources that inquired about occupation did not code responses to the level of detail required to identify health workers and were therefore excluded. From the WHO National Health Workforce Accounts,^29^ 2950 country-years of data were used, whereas 69 country-years of data from censuses and 1404 country-years of data from labour force participation surveys done between 1990 and 2019 were used. These sources provided data for 196 of the 204 countries and territories for which we produced estimates (appendix 2, figure S2) and covered locations that made up 99·9% of the world's population in 2019. The extracted indicators were total employment levels and the proportion of employed populations actively working in various occupations. Additional details on definitions and typical survey questions are included in appendix 1 (section 1).

### Definition of human resources for health

We analysed cadres of health workers identified in SDG indicator 3.c.1, as well as additional health worker cadres. Our data sources categorised occupations using a variety of coding systems, the most common of which was the ISCO. The ISCO applies a standard framework to classify occupations on the basis of skill level and degree of specialisation.^37^ Multiple versions of the ISCO exist and differ in their structure and level of detail. Although ISCO-08 was adopted more recently, ISCO-88 was the version used in the vast majority of included sources, and especially those earlier in the time series (1980–2008; see appendix 2, figure S3, for the uneven distribution across labour force surveys and censuses). We therefore defined our HRH categories using the ISCO-88 hierarchy, to minimise the inaccuracies inherent to converting across coding systems. After identifying ISCO-88 codes related to health care and consolidating similar occupations on that list, we were left with 16 HRH cadres. We mapped the coding systems of all included sources to our set of health-related occupations and split less-detailed codes as necessary using sources with more granular data. Additional details of this standardisation process are reported in appendix 1 (section 1).

The following are the 16 health worker cadres that we were able to estimate: physicians; nurses and midwives; dentists and dental assistants (dentistry personnel); pharmacists and pharmaceutical assistants (pharmaceutical personnel); clinical officers, medical assistants, and community health workers; medical imaging and therapeutic equipment technicians; health-care aides and ambulance workers; medical laboratory technicians; dietitians and nutritionists; optometrists and opticians; audiologists, speech therapists, and counsellors; physiotherapists and prosthetic technicians; psychologists; environmental health workers; home-based personal care workers; and traditional and complementary practitioners. It would have been preferable to further disaggregate some of these groupings to help better resolve important policy questions. For instance, community health workers play an important role in the global health workforce, yet the most granular ISCO-88 code for community health workers also includes clinical officers and medical assistants, precluding estimation of any of those individual positions.

### Adjusting data

We adjusted the WHO data to address inconsistencies in definitions, standards, and methods affecting lack of comparability in this data source. We matched 2636 WHO country-years of data across cadres with census or labour force survey datapoints for physicians, nurses and midwives, pharmacists, pharmacist technicians, dentists, and dental assistants. For each cadre, we first tested whether adjustments should be made using two separate lasso regressions with different sets of covariates: location indicators were included to test for location-specific adjustments, and region and super-region indicators were included to test for geographical adjustments to apply in locations that did not have matched pairs. In locations where we had matched pairs that were not estimated as zero by the lasso regression, we used the crosswalk package developed for GBD to estimate an adjustment factor for each location.^38^, ^39^ In locations where we did not have matched pairs, we used the regional and super-region indicators not estimated as zero in the lasso regression to adjust WHO data. Because matched pairs remained sparse in many locations and even in some super-regions, which prompted concerns about over-fitting, we included a Gaussian prior in our crosswalk model. Further information about the models and adjustments are available in appendix 1 (pp 18–29).

### Modelling health worker densities

We used spatiotemporal Gaussian process regression (ST-GPR) to estimate levels of HRH for missing geographies and years. ST-GPR is a flexible three-stage modelling approach used widely within GBD^40^ that draws strength across geography and time to produce full time series estimates with uncertainty intervals from data that are often unevenly distributed across space and time. Briefly, the first stage of the model fits a linear regression to the data with fixed effects on specified covariates. The second stage smooths the residuals between the regression fit and the data across time and geography to generate a non-linear trend that better follows available data in a location, region, and super-region. The third stage uses that trend as a mean function in a Gaussian process regression to account for input data variance and to generate uncertainty in the final estimates. The model leveraged available survey and census data along with related covariates, including Socio-demographic Index (SDI), total per capita health expenditure, and estimates of the professional workforce, to generate HRH densities by cadre and for all cadres together, for all 204 countries and territories from 1990 to 2019. Rescaling factors were applied to all component cadre results to ensure their consistency with estimates of total HRH and employment. More details on covariates and the strength and relevance of the ST-GPR method for modelling HRH are included in appendix 1 (section 1, pp 27–29).

Uncertainty in modelled estimates was derived from sampling uncertainty in the data and uncertainty from the ST-GPR models themselves and was propagated through all steps of the analysis. We produced 1000 draws of health worker densities for every cadre, location, and year, and calculated 95% uncertainty intervals (UIs) using the 2·5th and 97·5th percentiles of the corresponding distribution.

### SDI and UHC effective coverage index

We related our estimates of health workforce densities to two existing published indices capturing social and economic development and aspects of health-system performance.^32^, ^36^ First, SDI reflects levels of development through a composite indicator made up of a country's or territory's lag distributed income per capita, its total fertility rate among females younger than 25 years, and its mean educational attainment in years of completed school among females aged 15 years and older. Countries and territories were grouped into quintiles according to their 2019 SDI levels. Second, the UHC effective coverage index measures the use, quality, and efficacy of health service provision.^32^ The 23 indicators that comprise it capture a range of essential health services delivered across the life course, including interventions related to family planning, maternal and neonatal care, vaccination, and treatment for a variety of diseases including HIV, diabetes, and cancers. Each indicator is weighted according to the population health gains that the intervention could theoretically deliver in a given location and year, based on estimates of disease burden and intervention efficacy. Finally, an overall measure between 0 and 100 is constructed for every location and year, as the weighted average of all 23 indicators. Further details of the UHC effective coverage index are included in appendix 1 (section 2).

### Estimating the relationship between health worker densities and UHC effective coverage

In order to establish global evidence-based minimum thresholds for health worker densities, we used stochastic frontier meta-regression (SFM),^32^ an extension of traditional stochastic frontier analysis,^41^ to evaluate the relationship between various human resource inputs and the corresponding maximum expected UHC effective coverage. More details of this statistical approach are provided in appendix 1 (section 2). Briefly, we fit a production frontier to the combination of HRH estimates and corresponding values of UHC effective coverage. The production frontiers capture how efficiently a location is achieving a level of UHC effective coverage given its current HRH density. Because we are interested in examining the inputs of the production frontier, we also analyse the minimum HRH densities needed to achieve a given level of UHC effective coverage using the frontiers. The frontier values were estimated with an assumed distribution of efficiency across locations, as well as the known measurement uncertainty in UHC effective coverage. In this implementation of SFM, we used a flexible spline to estimate the functional form of the relationship between human resource densities and maximum possible UHC effective coverage. The spline was constrained to be monotonically increasing and concave based on a-priori expectations that were substantiated by preliminary analyses of modelled estimates. We used generalised trimming methods for systematic outlier detection, so that the most extreme 7·5% of observations were identified as outliers and excluded as the frontier was constructed.^42^, ^43^ The current implementation of SFM does not provide uncertainty in the fitted frontier, which precluded uncertainty estimation in the thresholds and corresponding health-worker shortages.

The cadres included in the frontier analyses were those specified in SDG indicator 3.c.1: physicians, nursing and midwifery personnel, dentistry personnel, and pharmaceutical personnel.^15^ We therefore generated four distinct production frontiers, each using all estimates for the cadre being analysed, for all locations and years. We determined minimum density thresholds for each health worker cadre to achieve performance targets of 80 out of 100 and 90 out of 100 on the UHC effective coverage index. For each performance target, we took the corresponding point on the frontier curve to represent the minimum level of HRH that would be required to obtain it.

Since the UHC effective coverage index measures effective coverage of essential health services, countries and territories should strive for the highest attainable index performance. Because SFM is fit to historical data, however, the fitted frontiers of the present study cannot estimate HRH needs for UHC effective coverage levels beyond those observed between 1990 and 2019. Given the small number of mostly high-income locations achieving UHC effective coverage levels of 90 or more, we chose to focus our discussion on the more stable and globally representative thresholds derived from a UHC target of 80.

The SFM also provides estimates of the productive efficiency of human resource use in generating UHC effective coverage for each location. Locations closer to the frontier are more efficient in this regard than locations far from the frontier. The frontier for a given HRH cadre is driven by locations that achieve a relatively high UHC with relatively low densities of that professional cadre.

Workforce thresholds for each of the specified HRH cadres represent minimum requirements to meet UHC effective coverage targets. It is important to note that they do not necessarily reflect an ideal skill mix for any given health system. Clearly, different locations achieve UHC using different skill mixes, which are likely to include allied health workers beyond the four cadres considered in the SFM analysis. Moreover, achieving the frontier level of UHC will also require additional contextual factors to be in place, such as adequate total health expenditure or the availability of medical equipment and infrastructure.

### Role of the funding source

The funder of the study had no role in study design, data collection, data analysis, data interpretation, or the writing of the report.

## Results

In 2019, the world had 104·0 million (95% UI 83·5–128·0) employed health workers. This total included 12·8 million (9·7–16·6) physicians, 29·8 million (23·3–37·7) nurses and midwives, 4·6 million (3·6–6·0) dentistry personnel, and 5·2 million (4·0–6·7) pharmaceutical personnel (appendix 2, table S1). We discuss the two largest HRH cadres here and provide additional details in appendix 2 (table S1).

**Figure 1:**
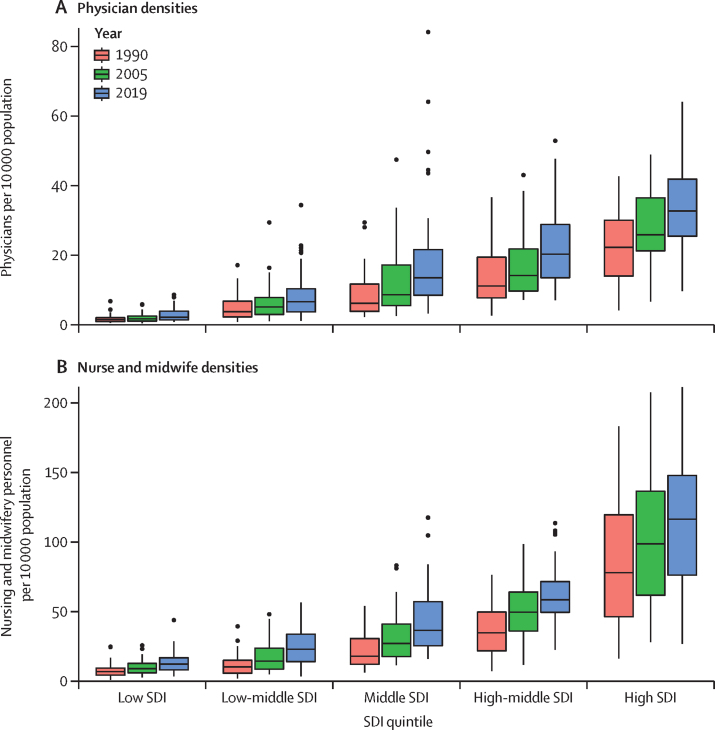
Physician densities (A) and nurse and midwife densities (B) by SDI quintile in 1990, 2005, and 2019 Boxplots show medians and IQRs. SDI=Socio-demographic Index.

**Figure 2:**
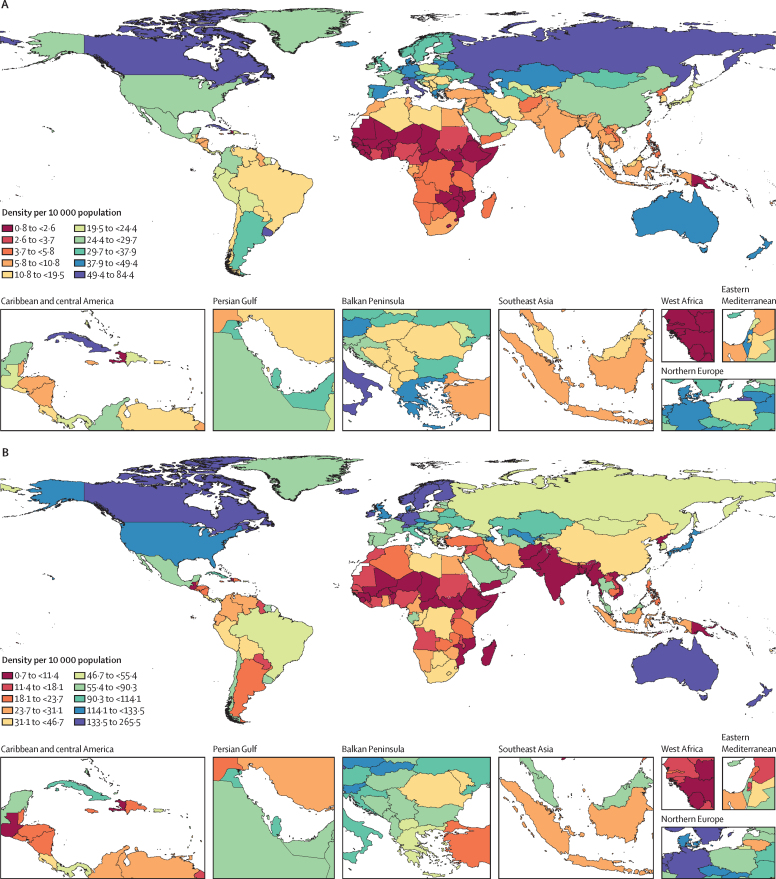
Density of physicians (A) and nurses and midwives (B) per 10 000 population by country and territory, 2019

In 2019, the global density of physicians was 16·7 (95% UI 12·6–21·6) per 10 000 population (table 1). There was more than a ten-fold difference in median physician densities between the lowest and highest SDI quintiles (figure 1A). Across GBD super-regions, densities ranged from 2·9 (2·1–4·0) per 10 000 population in sub-Saharan Africa to 38·3 (29·0–49·3) per 10 000 population in central Europe, eastern Europe, and central Asia (table 1). Physician densities were 10·8 per 10 000 or lower in sub-Saharan Africa, south Asia, and north Africa and the Middle East, whereas the remaining four GBD super-regions had densities of 19·5 per 10 000 or higher. Sizeable differences existed not only across super-regions^33^ in 2019, but also within them (figure 2A). Whereas the region of east Asia had a density of 26·5 (19·5–35·1) physicians per 10 000 population, southeast Asia had a density of 7·3 (5·0–10·2) per 10 000 population and Oceania had a density of 2·3 (1·6–3·3) per 10 000 population. Additionally, although eastern Europe had a density of 50·6 (38·8–64·2) per 10 000 population, central Europe had a much lower density of 22·2 (17·2–28·1) per 10 000 population. Even starker national-level differences within regions included Cuba, with a density of 84·4 (62·8–107·6) per 10 000 population, compared to Haiti, with a density of 2·1 (1·4–2·9) per 10 000 population, as well as the United Arab Emirates, with a density of 30·4 (21·4–41·9) per 10 000 population, compared to Afghanistan, with a density of 3·8 (2·6–5·3) per 10 000 population.

**Table 1:** Density of physicians, nursing and midwifery personnel, and other health workers for GBD super-regions, regions, and 204 countries and territories in 1990 and 2019

			**Physicians (95% UI)**			**Nursing and midwifery personnel (95% UI)**			**Other health workers (95% UI)**		
			Density per 10 000 population in 1990	Density per 10 000 population in 2019	Annualised rate of change 1990–2019 (%)	Density per 10 000 population in 1990	Density per 10 000 population in 2019	Annualised rate of change 1990–2019 (%)	Density per 10 000 population in 1990	Density per 10 000 population in 2019	Annualised rate of change 1990–2019 (%)
**Global**			**10·4 (7·7 to 13·7)**	**16·7 (12·6 to 21·6)**	**2·0% (−0·9 to 5·6)**	**23·3 (17·9 to 29·9)**	**38·6 (30·1 to 48·8)**	**2·1% (−0·7 to 5·5)**	**37·7 (30·9 to 45·3)**	**79·1 (65·4 to 95·1)**	**3·1% (0·2 to 6·6)**
Central Europe, eastern Europe, and central Asia*			**26·0 (19·9 to 33·5)**	**38·3 (29·0 to 49·3)**	**2·1% (−0·3 to 3·1)**	**45·2 (33·9 to 59·0)**	**73·5 (55·8 to 94·9)**	**1·8% (−0·7 to 2·7)**	**69·1 (58·3 to 81·5)**	**126·4 (105·8 to 150·4)**	**4·0% (1·3 to 5·1)**
	Central Asia		13·7 (9·7 to 18·7)	30·5 (21·7 to 41·6)	3·1% (1·0 to 7·1)	47·6 (34·4 to 64·7)	96·8 (71·2 to 128·0)	2·3% (0·4 to 4·2)	7·9 (7·0 to 9·0)	31·1 (25·8 to 37·0)	5·2% (2·8 to 7·6)
		Armenia	6·7 (4·7 to 9·5)	43·6 (32·2 to 57·2)	6·5% (4·9 to 8·0)	37·8 (27·8 to 50·6)	83·7 (62·8 to 107·5)	2·8% (1·4 to 4·2)	11·2 (8·6 to 14·3)	71·9 (56·5 to 89·0)	6·7% (5·1 to 8·1)
		Azerbaijan	14·4 (10·2 to 19·3)	44·6 (31·0 to 61·4)	3·9% (2·4 to 5·4)	53·8 (38·3 to 73·7)	117·4 (83·6 to 160·4)	2·7% (1·3 to 4·1)	9·9 (8·0 to 11·5)	53·3 (44·7 to 59·7)	6·4% (4·9 to 7·9)
		Georgia	18·9 (13·1 to 26·0)	49·8 (41·2 to 60·0)	3·3% (2·1 to 4·7)	36·3 (25·7 to 49·0)	56·9 (47·3 to 68·5)	1·6% (0·3 to 2·9)	23·9 (20·9 to 29·0)	62·3 (53·8 to 69·7)	3·4% (2·2 to 4·7)
		Kazakhstan	21·0 (15·0 to 28·3)	42·6 (29·8 to 58·8)	2·4% (0·9 to 4·0)	71·1 (51·3 to 96·5)	108·1 (79·7 to 143·2)	1·4% (−0·0 to 2·9)	16·1 (12·6 to 19·9)	66·6 (52·1 to 83·0)	4·4% (2·9 to 6·0)
		Kyrgyzstan	9·9 (7·1 to 13·4)	17·1 (11·6 to 24·1)	1·8% (0·3 to 3·5)	39·4 (28·1 to 53·5)	56·4 (40·0 to 76·4)	1·2% (−0·2 to 2·8)	4·6 (5·0 to 5·1)	10·4 (9·7 to 10·1)	4·1% (2·7 to 5·7)
		Mongolia	17·1 (11·8 to 22·8)	34·4 (28·0 to 41·6)	2·4% (1·1 to 3·8)	25·1 (18·4 to 33·7)	47·1 (39·0 to 55·6)	2·2% (0·9 to 3·4)	12·0 (10·8 to 13·6)	49·3 (43·6 to 58·9)	4·9% (3·6 to 6·2)
		Tajikistan	8·7 (6·2 to 11·9)	22·8 (16·1 to 31·3)	3·3% (1·8 to 5·0)	24·9 (18·1 to 34·3)	50·9 (37·1 to 69·2)	2·5% (0·9 to 3·9)	2·5 (1·9 to 2·8)	10·3 (10·0 to 10·7)	5·1% (3·6 to 6·7)
		Turkmenistan	11·4 (7·8 to 15·9)	30·6 (20·6 to 42·3)	3·4% (1·5 to 5·1)	37·2 (26·3 to 49·6)	83·9 (58·0 to 114·7)	2·8% (1·2 to 4·3)	2·8 (2·6 to 3·0)	27·6 (24·0 to 32·1)	5·5% (3·9 to 7·1)
		Uzbekistan	9·7 (6·9 to 13·5)	20·7 (14·3 to 28·9)	2·6% (1·0 to 4·0)	43·5 (31·8 to 59·1)	117·6 (87·0 to 153·9)	3·5% (2·0 to 4·8)	1·2 (0·2 to 3·5)	6·5 (5·9 to 7·9)	6·6% (5·1 to 8·0)
	Central Europe		15·8 (12·0 to 20·3)	22·2 (17·2 to 28·1)	1·9% (−0·3 to 4·8)	46·6 (36·9 to 58·5)	65·9 (52·3 to 81·7)	1·8% (−0·7 to 4·2)	55·1 (46·0 to 64·7)	127·6 (107·6 to 152·3)	3·5% (1·2 to 7·6)
		Albania	9·3 (6·8 to 12·5)	19·0 (15·0 to 23·7)	2·5% (1·1 to 3·7)	31·5 (24·0 to 41·3)	56·2 (45·8 to 69·0)	2·0% (0·8 to 3·2)	12·9 (9·6 to 16·5)	51·2 (43·5 to 58·1)	5·0% (3·8 to 6·3)
		Bosnia and Herzegovina	3·4 (2·3 to 4·8)	12·0 (8·4 to 17·0)	4·3% (2·7 to 6·0)	19·2 (13·7 to 25·9)	57·7 (41·7 to 77·8)	3·8% (2·4 to 5·2)	7·1 (5·6 to 8·9)	38·3 (29·8 to 46·7)	7·2% (5·8 to 8·7)
		Bulgaria	20·7 (15·3 to 27·6)	36·8 (26·2 to 49·8)	2·0% (0·4 to 3·5)	66·6 (50·8 to 86·4)	70·3 (51·2 to 93·7)	0·2% (−1·3 to 1·5)	65·8 (50·9 to 80·9)	185·6 (145·7 to 237·8)	4·0% (2·6 to 5·3)
		Croatia	16·2 (12·1 to 21·3)	28·8 (23·8 to 33·7)	2·0% (0·8 to 3·1)	43·4 (32·6 to 57·5)	93·1 (79·3 to 108·3)	2·7% (1·6 to 3·7)	30·4 (24·6 to 37·3)	102·7 (94·1 to 113·1)	4·4% (3·3 to 5·4)
		Czech Republic	22·2 (17·7 to 27·2)	37·3 (31·2 to 43·9)	1·8% (0·8 to 2·7)	91·6 (77·0 to 107·6)	116·5 (99·2 to 134·2)	0·8% (0·1 to 1·6)	62·0 (53·0 to 72·0)	162·9 (147·7 to 182·4)	3·3% (2·5 to 4·1)
		Hungary	15·0 (11·9 to 18·7)	17·0 (13·9 to 20·5)	0·4% (−0·6 to 1·4)	59·1 (48·0 to 71·8)	55·7 (46·5 to 65·3)	−0·2% (−1·1 to 0·7)	85·1 (73·2 to 97·0)	133·0 (117·9 to 147·1)	1·5% (0·6 to 2·4)
		Montenegro	11·7 (7·8 to 16·8)	18·6 (13·5 to 25·6)	1·6% (−0·1 to 3·3)	49·5 (34·0 to 66·4)	71·1 (50·7 to 94·8)	1·3% (−0·3 to 2·8)	26·8 (22·5 to 33·8)	83·9 (65·3 to 111·9)	2·5% (0·9 to 3·9)
		North Macedonia	10·8 (7·5 to 14·7)	19·3 (13·1 to 26·8)	2·0% (0·4 to 3·6)	25·3 (17·8 to 34·5)	49·4 (35·6 to 67·3)	2·3% (0·8 to 3·8)	20·3 (16·9 to 24·5)	72·0 (54·3 to 95·0)	4·3% (2·7 to 5·8)
		Poland	17·3 (13·1 to 22·3)	20·2 (14·6 to 27·3)	0·5% (−0·8 to 1·9)	49·6 (39·4 to 61·9)	55·8 (41·0 to 73·0)	0·4% (−0·8 to 1·7)	51·7 (42·7 to 63·1)	131·0 (103·2 to 167·4)	3·2% (1·9 to 4·4)
		Romania	16·1 (12·8 to 20·1)	17·4 (14·8 to 20·2)	0·3% (−0·7 to 1·2)	19·6 (15·1 to 25·4)	44·0 (37·5 to 51·3)	2·8% (1·8 to 3·9)	76·9 (66·5 to 87·3)	133·7 (120·7 to 149·4)	2·0% (1·2 to 2·8)
		Serbia	7·6 (5·1 to 10·8)	12·6 (8·7 to 17·2)	1·7% (0·2 to 3·4)	25·7 (18·0 to 35·4)	58·1 (42·2 to 77·2)	2·8% (1·4 to 4·4)	20·4 (15·7 to 23·9)	56·3 (45·0 to 69·0)	3·6% (2·2 to 5·1)
		Slovakia	18·1 (13·5 to 24·1)	32·0 (26·7 to 37·9)	2·0% (0·8 to 3·1)	77·9 (62·0 to 98·3)	116·3 (99·9 to 135·3)	1·4% (0·4 to 2·4)	57·5 (46·0 to 63·2)	175·2 (159·0 to 190·9)	3·7% (2·8 to 4·6)
		Slovenia	9·5 (7·2 to 12·4)	25·3 (19·1 to 32·2)	3·4% (2·0 to 4·7)	55·2 (43·8 to 68·5)	118·0 (94·1 to 148·0)	2·6% (1·5 to 3·7)	64·6 (52·8 to 76·6)	147·1 (119·2 to 174·7)	2·9% (1·9 to 3·9)
	Eastern Europe		35·4 (27·4 to 45·2)	50·6 (38·8 to 64·2)	1·3% (−0·5 to 3·6)	43·6 (32·1 to 57·6)	67·2 (50·9 to 87·4)	0·9% (−0·9 to 3·1)	95·3 (80·6 to 112·7)	168·2 (140·4 to 199·9)	3·5% (1·2 to 6·1)
		Belarus	21·3 (15·2 to 29·3)	43·7 (31·8 to 58·8)	2·5% (0·9 to 4·1)	53·1 (38·4 to 72·2)	106·1 (79·0 to 140·9)	2·4% (0·9 to 3·8)	31·2 (25·0 to 39·0)	150·2 (125·8 to 181·9)	4·6% (3·1 to 6·1)
		Estonia	27·4 (20·9 to 35·4)	27·9 (22·7 to 33·5)	0·1% (−1·0 to 1·1)	67·9 (53·1 to 85·6)	66·7 (55·2 to 80·7)	−0·0% (−1·1 to 1·0)	76·6 (64·3 to 89·4)	143·4 (126·5 to 159·3)	2·5% (1·6 to 3·5)
		Latvia	27·7 (21·6 to 34·8)	32·4 (26·7 to 38·9)	0·6% (−0·5 to 1·6)	72·9 (58·4 to 90·4)	66·5 (55·4 to 78·8)	−0·3% (−1·2 to 0·6)	49·6 (41·1 to 57·1)	129·8 (117·3 to 141·7)	3·5% (2·6 to 4·5)
		Lithuania	28·3 (21·2 to 36·8)	42·6 (35·7 to 50·4)	1·4% (0·3 to 2·6)	22·5 (16·7 to 29·6)	26·8 (20·7 to 32·9)	0·6% (−0·6 to 1·9)	65·3 (55·4 to 77·6)	201·8 (180·8 to 224·2)	4·1% (3·1 to 5·0)
		Moldova	11·5 (8·1 to 15·8)	24·0 (16·6 to 33·5)	2·5% (1·0 to 4·1)	28·0 (19·8 to 38·4)	38·2 (26·8 to 52·7)	1·1% (−0·3 to 2·5)	10·7 (9·2 to 14·0)	46·4 (35·9 to 54·7)	5·3% (3·8 to 6·7)
		Russia	42·7 (33·7 to 53·5)	58·4 (45·4 to 73·2)	1·1% (−0·1 to 2·2)	31·8 (24·0 to 41·5)	52·3 (40·1 to 67·7)	1·7% (0·4 to 3·0)	116·7 (100·3 to 137·3)	183·9 (153·7 to 216·8)	1·8% (0·8 to 2·8)
		Ukraine	20·3 (14·1 to 28·3)	30·3 (21·3 to 41·0)	1·4% (−0·1 to 3·0)	76·3 (54·2 to 102·1)	113·5 (84·3 to 148·3)	1·4% (0·0 to 2·8)	59·0 (45·5 to 71·2)	130·4 (107·1 to 162·0)	2·4% (1·0 to 3·9)
High income*			**22·2 (17·4–28·1)**	**33·4 (26·9 to 41·0)**	**1·5% (−0·8 to 2·4)**	**79·7 (63·5 to 99·0)**	**114·9 (94·7 to 137·7)**	**1·4% (−0·8 to 2·4)**	**128·0 (107·7 to 150·9)**	**243·9 (210·1 to 280·8)**	**2·6% (0·3 to 3·5)**
	Australasia		31·8 (24·6 to 40·5)	41·6 (30·7 to 54·4)	1·0% (−0·3 to 2·4)	175·9 (142·7 to 210·6)	152·3 (116·3 to 195·9)	−0·2% (−1·7 to 1·4)	124·3 (113·9 to 136·6)	259·1 (217·7 to 299·8)	2·5% (1·3 to 3·6)
		Australia	32·6 (25·2 to 41·3)	41·9 (31·0 to 54·8)	0·9% (−0·3 to 2·2)	183·1 (149·3 to 217·6)	151·6 (116·3 to 195·0)	−0·6% (−1·8 to 0·5)	122·3 (113·7 to 132·3)	241·6 (205·1 to 275·1)	2·7% (1·6 to 3·8)
		New Zealand	28·2 (21·3 to 36·8)	39·6 (29·5 to 52·1)	1·2% (−0·2 to 2·5)	140·4 (110·6 to 176·0)	155·9 (116·0 to 200·8)	0·4% (−0·9 to 1·6)	134·6 (114·8 to 157·6)	354·9 (286·6 to 434·5)	2·2% (1·1 to 3·4)
	High-income Asia Pacific		12·0 (8·5 to 16·7)	21·4 (16·2 to 27·7)	2·3% (0·4 to 4·3)	62·9 (45·1 to 85·9)	98·9 (78·0 to 124·1)	2·5% (0·4 to 5·0)	96·2 (76·1 to 122·2)	202·8 (166·4 to 240·0)	3·9% (1·3 to 6·5)
		Brunei	10·3 (7·0 to 14·8)	17·2 (12·1 to 23·9)	1·8% (0·2 to 3·3)	44·7 (31·1 to 61·9)	76·2 (54·4 to 102·7)	1·8% (0·4 to 3·4)	70·8 (55·5 to 90·0)	148·3 (110·1 to 197·4)	2·3% (0·8 to 3·7)
		Japan	13·9 (9·9 to 19·4)	23·5 (17·8 to 30·1)	1·9% (0·3 to 3·3)	80·0 (57·4 to 109·4)	119·2 (94·7 to 148·8)	1·4% (−0·1 to 2·8)	120·9 (95·9 to 152·9)	230·9 (190·5 to 270·5)	2·9% (1·6 to 4·2)
		Singapore	11·3 (7·6 to 15·6)	24·3 (16·7 to 33·3)	2·6% (1·0 to 4·3)	31·6 (21·7 to 43·9)	77·9 (55·3 to 104·8)	3·1% (1·5 to 4·7)	69·9 (54·5 to 89·9)	255·8 (195·8 to 333·2)	4·9% (3·5 to 6·4)
		South Korea	6·4 (4·5 to 9·0)	16·2 (12·2 to 21·5)	3·2% (1·7 to 4·8)	16·4 (11·8 to 22·3)	52·6 (40·7 to 67·4)	4·0% (2·5 to 5·5)	28·2 (21·5 to 37·2)	130·3 (105·9 to 157·5)	5·4% (4·1 to 6·8)
	High-income North America		22·0 (16·2 to 29·2)	29·7 (22·9 to 37·9)	1·0% (−0·7 to 2·4)	90·6 (68·8 to 116·7)	125·0 (100·6 to 152·7)	0·8% (−0·6 to 2·2)	158·0 (127·4 to 190·3)	280·4 (234·5 to 331·5)	2·1% (0·6 to 3·6)
		Canada	36·4 (27·9 to 47·6)	52·0 (38·6 to 68·7)	1·2% (−0·1 to 2·5)	122·4 (96·6 to 150·0)	141·5 (108·2 to 180·2)	0·5% (−0·7 to 1·6)	132·2 (110·9 to 152·6)	336·9 (268·1 to 421·3)	2·8% (1·7 to 3·9)
		Greenland	21·8 (14·4 to 31·0)	26·5 (17·7 to 37·9)	0·6% (−1·0 to 2·3)	71·8 (50·1 to 101·5)	89·1 (61·8 to 123·2)	0·7% (−0·8 to 2·4)	61·5 (47·5 to 74·1)	130·5 (100·3 to 162·6)	2·0% (0·6 to 3·4)
		USA	20·5 (14·9 to 27·2)	27·2 (21·2 to 34·5)	1·0% (−0·3 to 2·3)	87·2 (65·9 to 113·1)	123·2 (99·8 to 149·7)	1·2% (0·1 to 2·4)	160·8 (129·1 to 194·4)	274·2 (230·7 to 321·6)	1·6% (0·5 to 2·6)
	Southern Latin America		25·1 (18·4 to 34·2)	33·4 (25·7 to 42·8)	1·6% (−0·3 to 3·7)	15·0 (11·0 to 20·0)	37·4 (30·2 to 46·3)	3·0% (1·1 to 5·1)	74·0 (58·5 to 95·1)	155·7 (131·0 to 187·3)	3·4% (0·8 to 4·5)
		Argentina	30·4 (21·6 to 42·4)	37·5 (27·8 to 49·5)	0·7% (−0·8 to 2·3)	12·1 (8·1 to 17·3)	23·2 (16·7 to 31·7)	2·2% (0·6 to 4·0)	89·4 (68·0 to 119·1)	167·2 (135·4 to 208·7)	1·8% (0·4 to 3·2)
		Chile	11·4 (9·6 to 13·3)	17·5 (14·8 to 20·5)	1·4% (0·7 to 2·3)	18·0 (15·0 to 21·1)	66·2 (57·7 to 75·9)	4·5% (3·7 to 5·3)	37·2 (35·6 to 38·8)	123·0 (113·8 to 133·4)	4·1% (3·5 to 4·7)
		Uruguay	28·0 (21·3 to 36·3)	64·2 (56·4 to 72·6)	2·9% (1·9 to 3·9)	33·2 (24·3 to 44·2)	71·9 (63·2 to 81·6)	2·7% (1·6 to 3·8)	67·3 (55·4 to 79·5)	177·2 (163·8 to 193·2)	3·4% (2·5 to 4·3)
	Western Europe		26·1 (21·8 to 31·0)	41·0 (34·7 to 48·1)	1·5% (−0·9 to 4·8)	82·7 (70·5 to 96·3)	122·5 (105·4 to 141·1)	1·2% (−0·7 to 3·5)	127·6 (113·6 to 143·1)	243·5 (220·2 to 268·9)	2·5% (0·1 to 5·7)
		Andorra	33·5 (22·9 to 46·8)	40·9 (28·1 to 57·6)	0·7% (−1·1 to 2·3)	46·3 (32·1 to 64·4)	64·3 (43·6 to 88·8)	1·1% (−0·6 to 2·7)	319·4 (244·0 to 411·9)	488·9 (370·7 to 624·0)	1·2% (−0·2 to 2·5)
		Austria	26·6 (21·9 to 32·0)	45·6 (38·7 to 52·8)	1·9% (1·0 to 2·8)	64·8 (53·4 to 77·6)	109·8 (93·7 to 126·9)	1·8% (0·9 to 2·6)	103·0 (92·8 to 115·7)	231·3 (213·6 to 248·7)	2·9% (2·2 to 3·6)
		Belgium	26·6 (22·4 to 31·0)	32·7 (27·8 to 37·7)	0·7% (−0·0 to 1·5)	98·5 (85·8 to 111·9)	134·7 (118·3 to 154·2)	1·1% (0·4 to 1·8)	85·9 (81·4 to 91·9)	204·9 (193·4 to 217·2)	2·9% (2·3 to 3·5)
		Cyprus	21·3 (16·2 to 28·2)	32·1 (26·4 to 38·5)	1·4% (0·3 to 2·5)	32·7 (25·0 to 42·4)	64·3 (53·7 to 75·6)	2·4% (1·2 to 3·4)	42·8 (36·4 to 49·0)	107·6 (96·5 to 118·5)	3·2% (2·2 to 4·1)
		Denmark	33·2 (26·6 to 40·7)	40·8 (32·7 to 50·2)	0·7% (−0·3 to 1·8)	119·5 (96·4 to 144·9)	127·0 (100·9 to 158·9)	0·2% (−0·9 to 1·2)	413·2 (358·6 to 470·4)	509·4 (437·1 to 596·3)	0·8% (0·0 to 1·7)
		Finland	34·7 (27·3 to 44·4)	32·4 (26·5 to 39·2)	−0·3% (−1·3 to 0·9)	182·0 (146·0 to 224·0)	150·8 (128·0 to 176·7)	−0·6% (−1·5 to 0·3)	235·6 (205·8 to 272·5)	330·9 (295·4 to 363·6)	1·4% (0·6 to 2·2)
		France	31·9 (26·7 to 37·7)	24·8 (20·4 to 29·8)	−0·9% (−1·8 to 0·0)	72·2 (59·8 to 86·2)	87·7 (73·5 to 103·6)	0·7% (−0·2 to 1·5)	178·5 (156·6 to 199·4)	224·0 (201·0 to 253·7)	0·9% (0·2 to 1·7)
		Germany	30·0 (26·2 to 34·2)	46·9 (41·0 to 53·4)	1·5% (0·9 to 2·2)	95·0 (83·9 to 106·7)	176·1 (155·6 to 196·3)	2·1% (1·5 to 2·7)	131·7 (120·0 to 144·5)	265·8 (244·4 to 290·9)	2·3% (1·8 to 2·8)
		Greece	36·6 (30·8 to 43·7)	47·8 (40·6 to 55·9)	0·9% (0·1 to 1·7)	21·7 (17·8 to 26·2)	49·1 (41·9 to 57·2)	2·8% (2·0 to 3·7)	44·6 (39·0 to 50·3)	67·0 (59·8 to 74·3)	1·7% (0·9 to 2·4)
		Iceland	28·9 (22·7 to 36·8)	42·2 (34·8 to 50·4)	1·3% (0·2 to 2·4)	153·4 (124·3 to 186·8)	173·0 (147·1 to 205·0)	0·4% (−0·5 to 1·3)	154·2 (134·3 to 175·1)	317·6 (284·9 to 350·2)	2·4% (1·5 to 3·2)
		Ireland	6·1 (4·7 to 7·8)	25·3 (20·9 to 30·3)	4·9% (3·8 to 6·0)	79·7 (66·0 to 95·5)	147·7 (123·4 to 172·3)	2·1% (1·3 to 2·9)	44·4 (36·0 to 51·6)	213·2 (189·4 to 244·8)	5·8% (4·9 to 6·6)
		Israel	31·7 (24·2 to 40·7)	42·3 (32·9 to 52·8)	1·0% (−0·3 to 2·2)	44·8 (33·8 to 59·1)	62·8 (48·3 to 79·0)	1·2% (−0·1 to 2·5)	94·9 (77·8 to 112·8)	245·4 (205·8 to 289·7)	3·3% (2·2 to 4·5)
		Italy	28·5 (22·7 to 34·9)	53·0 (43·9 to 63·1)	2·1% (1·2 to 3·1)	52·8 (42·1 to 64·3)	93·0 (78·9 to 109·9)	1·9% (1·1 to 3·0)	56·7 (51·4 to 65·3)	136·4 (118·4 to 154·6)	3·0% (2·1 to 3·9)
		Luxembourg	17·9 (14·1 to 22·8)	34·8 (28·1 to 42·6)	2·3% (1·2 to 3·4)	49·6 (39·7 to 62·3)	85·1 (70·0 to 103·6)	1·9% (0·9 to 2·9)	78·0 (64·9 to 91·5)	204·6 (178·9 to 229·9)	3·4% (2·5 to 4·3)
		Malta	22·9 (15·9 to 32·6)	28·2 (20·0 to 39·1)	0·7% (−0·8 to 2·4)	48·3 (33·2 to 68·1)	105·3 (77·2 to 142·5)	2·7% (1·2 to 4·2)	54·3 (41·8 to 69·0)	161·3 (120·6 to 203·5)	3·9% (2·5 to 5·4)
		Monaco	24·9 (16·7 to 35·0)	51·2 (37·1 to 69·5)	2·5% (1·0 to 4·1)	142·8 (104·5 to 194·5)	165·2 (119·9 to 220·1)	0·5% (−0·9 to 1·9)	133·1 (102·0 to 170·2)	193·1 (151·9 to 250·0)	0·9% (−0·4 to 2·2)
		Netherlands	19·1 (15·5 to 23·6)	44·8 (37·5 to 53·4)	3·0% (2·0 to 3·8)	147·1 (122·4 to 172·4)	193·8 (163·7 to 226·9)	1·0% (0·1 to 1·7)	205·2 (184·5 to 231·6)	324·5 (301·3 to 350·3)	1·8% (1·0 to 2·5)
		Norway	26·4 (20·4 to 33·6)	36·7 (30·1 to 44·0)	1·1% (0·1 to 2·2)	166·8 (131·9 to 204·7)	211·0 (179·8 to 246·7)	0·8% (−0·1 to 1·8)	329·2 (279·6 to 386·1)	471·8 (417·1 to 526·5)	1·3% (0·5 to 2·1)
		Portugal	13·8 (10·9 to 17·1)	36·3 (29·7 to 44·0)	3·3% (2·3 to 4·4)	34·7 (27·8 to 43·7)	74·9 (62·3 to 89·3)	2·7% (1·6 to 3·6)	52·4 (45·9 to 60·3)	180·6 (160·3 to 204·2)	4·2% (3·3 to 5·2)
		San Marino	36·1 (25·1 to 49·7)	56·0 (39·6 to 76·6)	1·5% (0·0 to 3·1)	111·3 (76·8 to 152·3)	132·9 (94·4 to 179·3)	0·6% (−0·9 to 2·2)	149·0 (121·8 to 189·5)	280·2 (220·6 to 356·8)	1·8% (0·5 to 3·2)
		Spain	19·4 (16·0 to 23·0)	46·0 (38·9 to 53·0)	3·0% (2·2 to 3·8)	33·8 (28·2 to 40·1)	86·0 (72·6 to 100·0)	3·2% (2·3 to 4·1)	52·3 (45·2 to 59·5)	180·6 (163·9 to 196·6)	4·3% (3·6 to 5·1)
		Sweden	32·3 (25·3 to 40·8)	37·9 (31·9 to 44·7)	0·6% (−0·4 to 1·5)	139·9 (113·5 to 171·2)	148·9 (128·7 to 173·5)	0·2% (−0·6 to 1·1)	561·3 (473·8 to 660·3)	526·2 (472·5 to 575·4)	0·3% (−0·4 to 1·1)
		Switzerland	33·7 (28·1 to 40·1)	64·2 (55·1 to 74·6)	2·2% (1·4 to 3·0)	127·8 (110·5 to 147·3)	163·0 (142·0 to 186·7)	0·8% (0·2 to 1·6)	163·9 (148·6 to 178·7)	344·9 (314·5 to 369·3)	2·5% (1·9 to 3·2)
		UK	16·8 (14·5 to 19·3)	35·1 (30·0 to 41·1)	2·5% (1·8 to 3·3)	113·8 (102·3 to 127·4)	130·5 (113·7 to 148·3)	0·5% (−0·1 to 1·0)	101·9 (94·1 to 109·5)	300·1 (274·3 to 325·8)	3·4% (2·9 to 3·9)
Latin America and Caribbean*			**11·1 (7·9–15·0)**	**19·5 (14·6 to 25·5)**	**2·4% (−0·6 to 3·7)**	**17·0 (12·3 to 23·1)**	**44·3 (34·5 to 55·9)**	**2·3% (−0·9 to 3·4)**	**33·9 (26·9 to 42·3)**	**81·6 (67·6 to 98·2)**	**3·4% (1·0 to 4·2)**
	Andean Latin America		14·6 (10·4 to 19·5)	20·4 (15·7 to 26·4)	1·3% (−0·5 to 4·6)	14·0 (9·9 to 18·9)	32·9 (25·7 to 42·1)	3·1% (0·8 to 5·1)	20·2 (16·1 to 25·3)	55·9 (46·4 to 65·4)	3·6% (1·8 to 5·2)
		Bolivia	6·9 (5·4 to 8·6)	21·3 (18·7 to 24·3)	3·9% (3·0 to 4·8)	11·1 (8·9 to 13·8)	39·5 (34·6 to 44·6)	4·4% (3·5 to 5·2)	13·0 (11·5 to 14·0)	51·2 (48·2 to 54·2)	4·7% (3·9 to 5·4)
		Ecuador	14·5 (10·8 to 18·9)	19·6 (16·6 to 22·9)	1·1% (−0·1 to 2·2)	15·2 (11·2 to 19·9)	24·0 (20·7 to 27·6)	1·6% (0·5 to 2·7)	15·2 (12·4 to 19·0)	48·6 (44·5 to 53·4)	3·3% (2·2 to 4·4)
		Peru	16·9 (11·7 to 23·0)	20·5 (14·2 to 28·9)	0·7% (−0·9 to 2·3)	14·4 (9·6 to 19·9)	35·3 (25·1 to 48·6)	3·1% (1·5 to 4·8)	24·7 (19·2 to 31·5)	61·3 (46·7 to 75·5)	2·9% (1·5 to 4·4)
		Caribbean	13·2 (9·5 to 17·6)	29·2 (21·2 to 38·1)	3·1% (−1·0 to 7·5)	27·1 (19·4 to 36·4)	40·6 (29·6 to 53·8)	1·9% (−0·9 to 5·0)	33·4 (26·7 to 40·8)	96·5 (72·9 to 123·5)	3·4% (1·1 to 5·4)
		Antigua and Barbuda	2·6 (1·8 to 3·8)	22·2 (15·1 to 31·0)	7·4% (5·7 to 9·0)	30·5 (21·0 to 42·2)	58·5 (41·6 to 81·2)	2·2% (0·7 to 3·8)	57·0 (44·2 to 76·6)	127·9 (99·7 to 159·5)	2·3% (0·9 to 3·7)
		The Bahamas	7·9 (5·3 to 11·1)	22·7 (15·6 to 31·8)	3·6% (2·0 to 5·3)	34·2 (24·1 to 47·6)	57·7 (40·2 to 77·5)	1·8% (0·2 to 3·4)	86·0 (66·3 to 109·9)	313·6 (233·3 to 405·5)	4·0% (2·6 to 5·4)
		Barbados	8·8 (6·0 to 12·2)	23·1 (15·7 to 33·2)	3·3% (1·7 to 5·0)	43·2 (30·6 to 58·5)	41·3 (28·9 to 58·5)	−0·2% (−1·7 to 1·5)	60·5 (45·7 to 76·8)	169·5 (128·0 to 216·8)	3·4% (2·1 to 4·8)
		Belize	5·4 (3·6 to 7·8)	10·1 (6·8 to 14·3)	2·1% (0·6 to 4·0)	8·8 (6·0 to 12·8)	23·3 (16·1 to 32·1)	3·4% (1·7 to 5·2)	15·9 (12·3 to 20·5)	58·8 (46·1 to 76·1)	4·4% (2·9 to 6·0)
		Bermuda	17·6 (12·1 to 24·8)	30·8 (20·7 to 42·6)	2·0% (0·3 to 3·5)	74·1 (51·2 to 101·5)	96·0 (66·9 to 133·5)	0·9% (−0·7 to 2·5)	147·9 (112·0 to 189·6)	439·1 (331·6 to 557·4)	3·5% (2·2 to 4·9)
		Cuba	29·4 (21·7 to 38·4)	84·4 (62·8 to 107·6)	3·6% (2·2 to 5·0)	54·0 (39·4 to 70·8)	104·6 (78·4 to 135·5)	2·3% (0·9 to 3·7)	42·4 (35·5 to 49·2)	177·4 (132·5 to 227·8)	4·4% (3·2 to 5·9)
		Dominica	3·5 (2·4 to 5·1)	7·7 (5·2 to 10·7)	2·7% (1·0 to 4·4)	26·8 (18·8 to 37·7)	56·5 (40·6 to 77·0)	2·6% (1·1 to 4·1)	19·0 (14·7 to 23·2)	52·2 (39·8 to 68·2)	3·3% (1·8 to 4·9)
		Dominican Republic	8·6 (6·0 to 11·9)	22·1 (15·2 to 30·1)	3·3% (1·6 to 4·9)	12·6 (8·5 to 17·6)	19·3 (13·5 to 26·2)	1·4% (−0·1 to 3·2)	19·6 (16·0 to 24·7)	81·3 (63·4 to 102·3)	4·9% (3·4 to 6·2)
		Grenada	2·5 (1·7 to 3·6)	9·6 (6·4 to 13·6)	4·6% (2·9 to 6·3)	19·6 (13·9 to 27·0)	53·0 (37·1 to 72·6)	3·4% (1·9 to 5·0)	22·5 (17·1 to 28·2)	70·1 (55·8 to 89·0)	3·7% (2·1 to 5·1)
		Guyana	3·4 (2·2 to 4·8)	8·2 (5·6 to 11·6)	3·1% (1·3 to 4·7)	14·4 (9·9 to 20·2)	14·7 (10·0 to 20·3)	0·1% (−1·5 to 1·5)	17·0 (13·1 to 22·2)	51·0 (38·9 to 65·2)	3·6% (2·2 to 5·0)
		Haiti	2·7 (1·8 to 3·8)	2·1 (1·4 to 2·9)	−0·9% (−2·7 to 0·8)	2·4 (1·6 to 3·5)	9·3 (6·5 to 13·0)	4·7% (3·0 to 6·5)	10·7 (7·9 to 13·4)	17·5 (12·7 to 22·3)	1·7% (0·1 to 3·3)
		Jamaica	5·4 (3·6 to 7·6)	7·9 (5·2 to 11·4)	1·3% (−0·3 to 2·9)	14·8 (10·1 to 20·9)	23·7 (15·8 to 33·1)	1·6% (−0·1 to 3·2)	41·0 (32·1 to 51·9)	105·2 (81·2 to 141·0)	2·8% (1·4 to 4·2)
		Puerto Rico	9·5 (6·5 to 13·7)	15·3 (10·5 to 21·4)	1·7% (−0·1 to 3·2)	40·5 (28·3 to 55·9)	47·3 (32·8 to 65·5)	0·5% (−1·0 to 2·1)	72·1 (54·6 to 89·7)	169·7 (127·3 to 214·6)	2·6% (1·1 to 3·9)
		Saint Kitts and Nevis	4·9 (3·3 to 6·9)	21·0 (14·6 to 29·6)	5·1% (3·3 to 6·6)	52·9 (38·3 to 70·5)	57·0 (38·9 to 78·9)	0·3% (−1·3 to 1·7)	41·6 (32·0 to 55·7)	158·1 (119·8 to 199·2)	4·2% (2·7 to 5·6)
		Saint Lucia	3·1 (2·1 to 4·4)	13·1 (8·9 to 18·3)	4·9% (3·2 to 6·6)	18·8 (13·1 to 26·0)	37·3 (26·4 to 51·6)	2·4% (0·8 to 4·0)	32·0 (24·5 to 43·1)	90·9 (69·7 to 115·2)	3·4% (1·9 to 4·8)
		Saint Vincent and the Grenadines	4·0 (2·7 to 5·7)	5·2 (3·4 to 7·3)	0·9% (−0·9 to 2·5)	21·3 (14·9 to 29·7)	60·2 (42·9 to 81·7)	3·6% (2·0 to 5·1)	15·2 (12·2 to 19·2)	32·4 (25·0 to 40·8)	2·4% (0·8 to 3·9)
		Suriname	7·3 (5·0 to 10·2)	20·6 (15·1 to 27·4)	3·6% (2·2 to 5·2)	30·3 (20·9 to 42·1)	55·6 (40·2 to 72·8)	2·1% (0·6 to 3·6)	68·3 (52·1 to 89·4)	112·7 (94·5 to 138·5)	2·4% (1·1 to 3·7)
		Trinidad and Tobago	4·4 (3·2 to 6·1)	18·7 (13·5 to 25·6)	4·9% (3·5 to 6·6)	14·5 (10·8 to 19·3)	27·2 (19·8 to 36·6)	2·2% (0·7 to 3·6)	33·5 (26·4 to 40·1)	101·0 (73·2 to 133·0)	4·0% (2·8 to 5·3)
		Virgin Islands	12·0 (8·0 to 17·0)	20·7 (14·1 to 29·7)	1·9% (0·2 to 3·6)	49·9 (34·3 to 70·9)	61·0 (41·4 to 84·9)	0·7% (−0·9 to 2·3)	94·6 (72·8 to 121·5)	248·7 (190·2 to 312·0)	3·3% (1·9 to 4·7)
	Central Latin America		12·8 (9·0 to 17·5)	23·5 (17·3 to 31·4)	1·8% (0·0 to 3·8)	17·3 (12·5 to 23·5)	40·7 (30·9 to 52·5)	2·8% (−1·3 to 5·6)	33·6 (26·5 to 42·2)	79·5 (63·4 to 98·7)	3·4% (0·6 to 5·8)
		Colombia	15·4 (10·8 to 21·2)	25·9 (18·3 to 36·2)	1·8% (0·2 to 3·4)	10·7 (7·5 to 15·1)	26·4 (19·0 to 36·5)	3·1% (1·5 to 4·7)	35·0 (26·5 to 43·9)	79·0 (60·8 to 104·6)	2·4% (0·9 to 3·9)
		Costa Rica	14·4 (10·3 to 19·1)	18·7 (13·8 to 24·3)	0·9% (−0·6 to 2·4)	12·3 (8·6 to 17·2)	35·8 (26·9 to 46·4)	3·7% (2·1 to 5·3)	36·5 (30·2 to 43·7)	73·0 (59·6 to 90·5)	2·5% (1·2 to 3·9)
		El Salvador	9·5 (7·7 to 11·6)	15·7 (12·9 to 18·8)	1·7% (0·8 to 2·7)	10·5 (8·6 to 12·7)	23·3 (19·7 to 27·6)	2·8% (1·8 to 3·7)	9·8 (8·8 to 10·7)	35·4 (31·0 to 39·5)	4·2% (3·2 to 5·0)
		Guatemala	10·9 (7·8 to 15·0)	20·6 (14·4 to 28·6)	2·2% (0·7 to 3·7)	4·0 (2·7 to 5·7)	3·5 (2·3 to 5·0)	−0·5% (−2·4 to 1·2)	13·2 (9·7 to 17·4)	45·8 (34·7 to 59·8)	4·0% (2·5 to 5·5)
		Honduras	3·6 (2·5 to 5·2)	8·4 (5·9 to 11·7)	2·9% (1·2 to 4·5)	10·3 (7·4 to 13·8)	18·4 (13·0 to 25·0)	2·0% (0·5 to 3·4)	4·5 (3·9 to 5·3)	22·1 (17·4 to 27·1)	5·1% (3·6 to 6·5)
		Mexico	12·9 (8·9 to 17·7)	26·6 (20·0 to 34·8)	2·5% (1·0 to 4·0)	21·1 (15·1 to 28·6)	57·7 (44·6 to 72·6)	3·5% (2·0 to 4·9)	40·2 (31·8 to 51·2)	102·2 (82·7 to 124·4)	2·6% (1·3 to 3·9)
		Nicaragua	7·0 (5·1 to 9·2)	10·2 (7·3 to 14·3)	1·3% (−0·1 to 2·8)	5·5 (3·9 to 7·5)	23·5 (16·9 to 31·9)	5·0% (3·5 to 6·6)	9·9 (8·2 to 11·8)	32·3 (24·8 to 40·5)	4·2% (2·9 to 5·6)
		Panama	11·8 (8·7 to 15·4)	19·5 (13·7 to 26·5)	1·8% (0·3 to 3·2)	23·6 (17·6 to 31·5)	54·0 (39·5 to 71·5)	2·9% (1·5 to 4·2)	29·4 (23·9 to 35·2)	87·6 (67·5 to 113·9)	3·8% (2·4 to 5·1)
		Venezuela	13·2 (9·6 to 17·9)	18·8 (13·2 to 25·6)	1·2% (−0·4 to 2·8)	23·5 (17·1 to 31·2)	27·9 (20·0 to 38·4)	0·6% (−1·0 to 2·2)	28·9 (23·1 to 34·1)	41·2 (32·6 to 49·8)	1·1% (−0·3 to 2·7)
	Tropical Latin America		7·9 (5·6 to 10·5)	12·8 (10·0 to 15·9)	2·3% (0·5 to 4·5)	15·1 (11·0 to 20·6)	52·2 (42·0 to 64·1)	2·7% (−0·7 to 5·4)	37·8 (30·1 to 47·1)	88·1 (77·2 to 101·7)	3·2% (1·6 to 5·2)
		Brazil	8·0 (5·6 to 10·5)	12·7 (9·9 to 15·7)	1·6% (0·3 to 3·0)	15·1 (11·1 to 20·6)	53·3 (43·0 to 65·5)	4·4% (3·0 to 5·7)	38·3 (30·5 to 47·7)	88·9 (78·0 to 102·3)	2·6% (1·4 to 3·8)
		Paraguay	6·7 (4·8 to 9·2)	16·8 (12·0 to 22·4)	3·2% (1·4 to 4·7)	13·7 (9·9 to 18·2)	16·3 (11·5 to 22·2)	0·6% (−1·0 to 2·2)	18·3 (14·7 to 23·1)	64·5 (51·2 to 81·9)	4·0% (2·5 to 5·4)
North Africa and Middle East*			**4·4 (3·0 to 6·0)**	**10·8 (8·0 to 14·3)**	**2·7% (0·7 to 5·5)**	**9·6 (6·8 to 13·1)**	**25·8 (19·6 to 33·5)**	**3·0% (−0·3 to 5·5)**	**6·7 (5·5 to 8·3)**	**30·5 (24·7 to 37·1)**	**4·0% (1·9 to 6·9)**
		Afghanistan	1·9 (1·3 to 2·7)	3·8 (2·6 to 5·3)	2·4% (0·8 to 4·1)	4·9 (3·5 to 6·9)	4·5 (3·1 to 6·3)	−0·3% (−1·9 to 1·3)	1·9 (1·4 to 2·4)	4·8 (4·1 to 6·0)	2·6% (1·1 to 4·1)
		Algeria	4·2 (2·8 to 5·8)	13·8 (9·6 to 19·5)	4·1% (2·5 to 5·8)	9·1 (6·5 to 12·6)	21·8 (15·2 to 30·5)	3·0% (1·4 to 4·6)	2·8 (2·2 to 3·3)	21·6 (16·6 to 26·7)	4·9% (3·3 to 6·4)
		Bahrain	10·5 (7·2 to 14·8)	17·6 (12·6 to 23·6)	1·8% (0·2 to 3·3)	34·7 (23·6 to 47·0)	58·4 (42·4 to 78·9)	1·8% (0·4 to 3·3)	29·5 (23·2 to 37·2)	114·7 (86·8 to 146·6)	4·4% (3·1 to 6·0)
		Egypt	6·1 (4·3 to 8·4)	10·8 (8·9 to 12·8)	2·0% (0·8 to 3·4)	13·1 (9·6 to 17·7)	25·8 (21·9 to 30·1)	2·4% (1·2 to 3·6)	9·4 (7·8 to 11·1)	25·4 (24·1 to 27·3)	3·8% (2·6 to 5·0)
		Iran	3·1 (2·2 to 4·3)	12·2 (9·2 to 15·9)	4·7% (3·1 to 6·3)	6·9 (5·1 to 9·4)	27·1 (20·5 to 34·4)	4·8% (3·2 to 6·2)	5·5 (4·4 to 6·8)	29·7 (24·2 to 37·1)	5·4% (3·9 to 6·9)
		Iraq	2·9 (2·0 to 4·0)	6·3 (4·7 to 8·4)	2·7% (1·1 to 4·2)	6·2 (4·4 to 8·3)	19·1 (14·7 to 24·3)	3·9% (2·5 to 5·4)	7·3 (5·7 to 9·8)	23·5 (19·6 to 27·3)	4·9% (3·4 to 6·3)
		Jordan	8·7 (6·2 to 11·7)	13·0 (10·4 to 16·3)	1·4% (0·0 to 2·8)	20·8 (15·4 to 27·6)	38·6 (31·4 to 47·2)	2·2% (0·9 to 3·4)	5·4 (4·7 to 5·7)	34·8 (29·0 to 39·8)	3·0% (1·7 to 4·2)
		Kuwait	15·6 (10·7 to 21·8)	31·4 (21·6 to 43·5)	2·4% (0·9 to 3·9)	49·6 (34·0 to 68·6)	105·5 (76·0 to 145·2)	2·6% (1·1 to 4·1)	42·5 (33·9 to 51·9)	110·3 (87·9 to 139·9)	2·8% (1·3 to 4·1)
		Lebanon	13·3 (9·1 to 18·2)	22·4 (16·1 to 30·0)	1·8% (0·2 to 3·3)	7·3 (4·9 to 10·1)	22·6 (15·9 to 30·6)	3·9% (2·3 to 5·5)	11·8 (9·6 to 15·5)	50·7 (38·8 to 67·3)	4·1% (2·6 to 5·5)
		Libya	5·7 (3·9 to 8·0)	11·1 (7·9 to 14·9)	2·3% (0·8 to 3·9)	18·5 (13·3 to 25·1)	43·6 (31·6 to 58·7)	2·9% (1·6 to 4·5)	3·7 (3·1 to 4·8)	26·9 (20·6 to 35·6)	4·1% (2·6 to 5·6)
		Morocco	2·9 (2·0 to 4·1)	6·6 (4·6 to 9·1)	2·8% (1·2 to 4·5)	5·1 (3·6 to 7·1)	13·5 (9·5 to 18·4)	3·4% (1·8 to 4·8)	3·2 (2·4 to 4·1)	10·0 (7·5 to 12·6)	3·4% (2·0 to 4·7)
		Oman	6·3 (4·3 to 8·8)	22·2 (16·1 to 30·2)	4·3% (2·8 to 6·0)	20·5 (14·7 to 28·5)	60·9 (44·3 to 82·3)	3·8% (2·3 to 5·2)	7·8 (6·2 to 10·1)	54·4 (41·5 to 71·9)	6·7% (5·2 to 8·2)
		Palestine	3·8 (2·6 to 5·3)	13·5 (9·7 to 18·6)	4·4% (2·9 to 6·0)	3·6 (2·5 to 5·2)	13·1 (9·4 to 17·5)	4·4% (2·9 to 6·0)	3·3 (2·7 to 4·2)	8·5 (6·7 to 11·1)	2·9% (1·4 to 4·4)
		Qatar	19·7 (13·6 to 27·1)	33·8 (23·1 to 46·4)	1·9% (0·3 to 3·5)	44·0 (30·5 to 61·0)	106·8 (75·4 to 144·1)	3·1% (1·6 to 4·5)	45·7 (37·8 to 55·8)	144·4 (109·4 to 198·3)	3·6% (2·1 to 5·0)
		Saudi Arabia	6·4 (4·3 to 8·9)	25·4 (18·2 to 34·5)	4·8% (3·2 to 6·5)	16·2 (11·3 to 22·1)	68·8 (50·0 to 92·4)	5·0% (3·4 to 6·6)	11·5 (9·2 to 14·8)	77·4 (58·7 to 99·8)	5·4% (3·9 to 6·8)
		Sudan	1·7 (1·1 to 2·4)	3·6 (2·4 to 5·0)	2·6% (0·9 to 4·5)	5·7 (3·8 to 7·9)	14·0 (9·9 to 19·0)	3·1% (1·5 to 4·8)	1·2 (1·1 to 1·2)	9·1 (7·0 to 11·5)	5·5% (3·9 to 7·1)
		Syria	3·9 (2·7 to 5·3)	7·2 (5·0 to 10·1)	2·2% (0·7 to 3·8)	7·0 (5·0 to 9·7)	12·5 (8·5 to 17·2)	2·0% (0·4 to 3·4)	3·9 (3·1 to 4·9)	12·7 (10·2 to 15·9)	3·8% (2·3 to 5·3)
		Tunisia	4·2 (3·0 to 5·9)	10·1 (7·2 to 13·8)	3·1% (1·4 to 4·7)	12·4 (8·8 to 16·9)	26·1 (18·7 to 35·7)	2·6% (1·1 to 4·1)	3·9 (3·1 to 4·4)	15·8 (11·3 to 18·4)	3·3% (1·7 to 4·9)
		Turkey	4·9 (3·4 to 6·8)	9·9 (7·7 to 12·8)	2·4% (1·1 to 3·9)	9·7 (6·6 to 13·4)	23·0 (18·0 to 29·4)	3·0% (1·5 to 4·5)	9·4 (7·8 to 11·7)	41·5 (34·1 to 48·1)	4·4% (3·1 to 5·8)
		United Arab Emirates	13·9 (9·4 to 19·2)	30·4 (21·4 to 41·9)	2·7% (1·2 to 4·3)	49·0 (35·4 to 65·9)	88·2 (64·8 to 120·0)	2·0% (0·5 to 3·6)	44·9 (33·4 to 58·7)	203·7 (148·8 to 259·9)	4·5% (3·1 to 6·0)
	Yemen		1·7 (1·1 to 2·4)	3·7 (2·5 to 5·4)	2·8% (1·2 to 4·5)	3·3 (2·3 to 4·6)	8·1 (5·7 to 11·0)	3·0% (1·5 to 4·6)	2·0 (1·6 to 2·4)	5·0 (4·0 to 6·0)	3·4% (1·9 to 5·1)
South Asia*			**3·8 (2·7 − 5·2)**	**6·5 (4·8 to 8·5)**	**2·4% (0·6 to 4·3)**	**3·5 (2·4 to 4·8)**	**9·7 (7·3 to 12·8)**	**3·4% (1·5 to 5·3)**	**9·6 (7·3 to 12·5)**	**30·3 (24·5 to 37·3)**	**3·5% (1·5 to 5·2)**
		Bangladesh	2·6 (1·8 to 3·8)	6·5 (4·5 to 9·1)	3·2% (1·5 to 4·9)	2·2 (1·5 to 3·1)	5·0 (3·4 to 7·1)	2·8% (1·1 to 4·7)	10·1 (7·5 to 13·2)	31·7 (23·9 to 41·7)	4·0% (2·5 to 5·5)
		Bhutan	2·7 (1·8 to 3·9)	6·1 (4·2 to 8·6)	2·8% (1·1 to 4·5)	9·5 (6·5 to 13·2)	28·4 (20·1 to 39·3)	3·8% (2·3 to 5·4)	16·8 (12·6 to 22·7)	56·9 (42·0 to 72·7)	4·0% (2·6 to 5·5)
		India	3·8 (2·7 to 5·3)	6·2 (4·6 to 8·3)	1·7% (0·1 to 3·3)	3·5 (2·4 to 4·8)	10·1 (7·5 to 13·2)	3·7% (2·1 to 5·3)	9·4 (7·2 to 12·4)	30·8 (25·0 to 38·2)	3·7% (2·2 to 5·0)
		Nepal	3·5 (2·3 to 5·0)	6·8 (4·6 to 9·8)	2·3% (0·6 to 4·0)	8·5 (6·0 to 11·9)	27·8 (19·8 to 39·5)	4·1% (2·5 to 5·7)	11·9 (8·9 to 14·6)	22·7 (17·7 to 25·9)	2·4% (0·9 to 3·8)
		Pakistan	4·2 (3·0 to 5·9)	7·8 (6·3 to 9·7)	2·1% (0·8 to 3·5)	4·0 (2·8 to 5·4)	8·3 (6·6 to 10·2)	2·5% (1·2 to 4·0)	9·6 (7·3 to 12·5)	26·6 (22·5 to 30·6)	3·4% (2·1 to 4·7)
Southeast Asia, east Asia, and Oceania*			**7·9 (5·6 to 11·1)**	**20·3 (14·8 to 27·1)**	**2·1% (−1·2 to 3·3)**	**8·4 (6·0 to 11·5)**	**29·9 (21·9 to 40·2)**	**2·3% (−0·6 to 3·6)**	**14·9 (11·2 to 18·8)**	**71·1 (54·9 to 90·8)**	**3·1% (0·4 to 4·3)**
	East Asia		9·8 (7·0 to 13·8)	26·5 (19·5 to 35·1)	2·7% (−1·2 to 4·6)	6·5 (4·6 to 8·9)	31·6 (23·4 to 42·3)	4·1% (1·5 to 6·6)	13·9 (10·3 to 17·5)	80·1 (61·9 to 102·5)	4·3% (0·3 to 7·4)
		China	10·0 (7·1 to 14·0)	27·2 (20·0 to 36·0)	3·5% (1·9 to 4·9)	6·3 (4·5 to 8·7)	31·6 (23·4 to 42·4)	5·6% (4·1 to 6·9)	13·7 (10·1 to 17·2)	81·1 (62·6 to 103·9)	6·3% (4·9 to 7·6)
		North Korea	5·0 (3·3 to 7·1)	4·9 (3·4 to 7·0)	−0·0% (−1·7 to 1·6)	4·7 (3·1 to 6·8)	10·1 (6·9 to 14·1)	2·7% (1·0 to 4·3)	12·0 (9·7 to 15·9)	16·3 (12·3 to 21·1)	1·4% (−0·2 to 2·9)
		Taiwan (province of China)	4·1 (2·8 to 5·8)	9·6 (7·1 to 12·6)	3·0% (1·4 to 4·7)	17·2 (12·1 to 23·6)	55·3 (42·7 to 69·4)	4·0% (2·6 to 5·5)	29·7 (23·0 to 36·8)	91·1 (74·7 to 109·4)	4·3% (2·9 to 5·7)
	Oceania		1·8 (1·2 to 2·7)	2·3 (1·6 to 3·3)	1·5% (−1·4 to 4·3)	10·6 (7·4 to 14·8)	11·4 (7·9 to 15·8)	1·8% (−0·7 to 7·6)	11·3 (8·5 to 14·4)	18·2 (14·0 to 23·1)	2·3% (0·2 to 5·2)
		American Samoa	6·8 (4·5 to 10·0)	7·0 (4·7 to 10·0)	0·1% (−1·6 to 1·9)	38·0 (26·5 to 51·3)	50·3 (36·0 to 69·1)	0·9% (−0·6 to 2·4)	32·8 (26·9 to 39·9)	51·4 (40·5 to 65·2)	1·4% (−0·1 to 2·9)
		Cook Islands	9·6 (6·3 to 13·7)	15·0 (10·6 to 20·8)	1·5% (−0·0 to 3·2)	37·0 (26·2 to 52·8)	69·6 (50·6 to 93·4)	2·2% (0·7 to 3·6)	47·9 (37·8 to 59·3)	88·8 (69·4 to 115·6)	1·8% (0·5 to 3·3)
		Federated States of Micronesia	6·7 (4·7 to 9·2)	6·2 (4·1 to 8·8)	−0·3% (−2·1 to 1·4)	2·0 (1·4 to 2·9)	18·0 (12·3 to 24·9)	7·5% (5·8 to 9·3)	15·0 (11·4 to 20·4)	26·8 (21·1 to 34·2)	1·8% (0·2 to 3·3)
		Fiji	4·2 (2·8 to 5·9)	8·4 (5·7 to 11·8)	2·4% (0·8 to 4·0)	20·6 (14·4 to 28·3)	34·4 (24·3 to 45·8)	1·8% (0·1 to 3·2)	21·5 (15·8 to 27·5)	39·1 (30·5 to 52·3)	2·3% (0·7 to 3·8)
		Guam	12·5 (8·4 to 18·4)	9·9 (6·8 to 13·7)	−0·8% (−2·5 to 0·9)	79·1 (57·4 to 108·0)	80·2 (56·4 to 110·7)	0·0% (−1·6 to 1·5)	105·0 (79·7 to 132·0)	128·9 (98·0 to 161·2)	0·8% (−0·5 to 2·3)
		Kiribati	0·9 (0·6 to 1·4)	2·3 (1·6 to 3·4)	3·1% (1·2 to 4·9)	13·8 (9·5 to 18·7)	34·9 (25·2 to 47·7)	3·2% (1·7 to 4·8)	2·2 (1·8 to 2·9)	11·6 (9·1 to 14·6)	4·0% (2·5 to 5·7)
		Marshall Islands	3·0 (2·0 to 4·2)	5·7 (3·9 to 8·3)	2·2% (0·5 to 4·0)	15·1 (10·8 to 21·0)	33·7 (24·2 to 45·9)	2·8% (1·3 to 4·2)	7·1 (5·4 to 8·6)	22·6 (16·2 to 29·7)	3·0% (1·4 to 4·6)
		Nauru	7·0 (4·8 to 9·7)	8·3 (5·8 to 11·3)	0·6% (−1·0 to 2·2)	29·0 (20·5 to 39·5)	45·8 (33·6 to 60·7)	1·6% (0·2 to 3·0)	13·7 (10·6 to 18·3)	29·6 (19·7 to 41·2)	2·0% (0·5 to 3·5)
		Niue	8·5 (5·7 to 11·8)	13·5 (9·3 to 19·6)	1·6% (−0·1 to 3·4)	35·4 (24·6 to 48·4)	64·5 (46·8 to 87·3)	2·1% (0·6 to 3·6)	28·6 (21·7 to 36·7)	52·5 (39·8 to 66·9)	2·2% (0·8 to 3·7)
		Northern Mariana Islands	11·1 (7·6 to 15·8)	10·7 (7·4 to 15·7)	−0·1% (−1·8 to 1·7)	64·9 (45·7 to 87·5)	82·3 (57·5 to 111·8)	0·9% (−0·7 to 2·3)	69·1 (55·4 to 87·8)	102·4 (79·8 to 132·3)	1·0% (−0·5 to 2·4)
		Palau	10·7 (7·2 to 15·2)	15·8 (11·0 to 21·9)	1·3% (−0·3 to 3·0)	44·5 (31·4 to 60·9)	71·5 (51·4 to 97·2)	1·6% (0·2 to 3·2)	45·1 (35·3 to 60·0)	89·4 (65·6 to 114·3)	1·9% (0·4 to 3·5)
		Papua New Guinea	0·9 (0·6 to 1·3)	1·5 (1·0 to 2·2)	1·8% (−0·1 to 3·7)	6·3 (4·3 to 9·0)	6·2 (4·2 to 8·9)	−0·1% (−1·9 to 1·6)	7·3 (5·4 to 9·4)	15·1 (11·6 to 18·8)	2·2% (0·6 to 4·0)
		Samoa	3·1 (2·1 to 4·4)	3·1 (2·1 to 4·5)	−0·0% (−1·6 to 1·7)	11·6 (8·1 to 16·3)	17·0 (12·2 to 23·3)	1·3% (−0·2 to 2·9)	9·5 (6·9 to 11·9)	15·6 (11·6 to 19·8)	2·0% (0·5 to 3·4)
		Solomon Islands	0·9 (0·6 to 1·3)	2·4 (1·6 to 3·4)	3·5% (1·8 to 5·2)	6·4 (4·6 to 9·1)	23·5 (17·0 to 31·2)	4·5% (3·1 to 5·9)	3·1 (2·1 to 3·8)	13·8 (10·3 to 19·2)	4·7% (3·1 to 6·2)
		Tokelau	3·9 (2·6 to 5·7)	6·2 (4·1 to 8·7)	1·6% (−0·1 to 3·2)	17·7 (12·4 to 24·2)	39·9 (28·0 to 54·8)	2·8% (1·2 to 4·3)	9·8 (7·8 to 12·6)	30·2 (22·9 to 38·5)	3·8% (2·2 to 5·3)
		Tonga	2·1 (1·4 to 3·1)	3·9 (2·6 to 5·5)	2·1% (0·3 to 3·9)	17·9 (12·9 to 24·2)	27·8 (19·7 to 37·1)	1·5% (0·0 to 3·0)	8·5 (6·6 to 10·3)	13·6 (10·2 to 19·0)	1·8% (0·2 to 3·3)
		Tuvalu	4·7 (3·2 to 6·7)	8·7 (5·9 to 11·9)	2·1% (0·4 to 3·8)	23·8 (17·0 to 32·9)	34·9 (25·0 to 46·6)	1·3% (−0·1 to 2·7)	8·0 (6·7 to 9·6)	22·1 (17·1 to 28·5)	2·6% (1·1 to 4·1)
		Vanuatu	0·8 (0·5 to 1·2)	2·0 (1·4 to 2·9)	3·2% (1·5 to 5·0)	12·1 (8·8 to 16·3)	18·3 (12·8 to 25·8)	1·4% (−0·1 to 2·9)	4·4 (3·3 to 5·9)	16·6 (12·6 to 19·9)	4·1% (2·5 to 5·8)
	Southeast Asia		3·1 (2·2 to 4·3)	7·3 (5·0 to 10·2)	2·9% (−0·7 to 8·2)	13·4 (9·7 to 18·2)	26·6 (19·0 to 35·9)	2·8% (−0·5 to 6·9)	17·6 (13·6 to 22·5)	52·4 (40·5 to 66·5)	4·0% (1·7 to 7·6)
		Cambodia	6·7 (5·1 to 8·6)	8·6 (6·5 to 11·0)	0·9% (−0·4 to 2·1)	4·3 (3·1 to 5·6)	19·5 (15·2 to 24·2)	5·3% (3·9 to 6·5)	10·1 (8·8 to 11·5)	36·2 (31·8 to 41·8)	4·3% (3·2 to 5·3)
		Indonesia	2·1 (1·4 to 3·1)	7·3 (4·9 to 10·7)	4·3% (2·6 to 6·0)	9·9 (6·9 to 13·7)	24·0 (16·5 to 33·0)	3·1% (1·5 to 4·7)	19·2 (14·5 to 25·4)	55·1 (41·7 to 70·9)	3·6% (2·1 to 5·0)
		Laos	3·0 (2·1 to 4·2)	5·8 (4·0 to 8·2)	2·3% (0·5 to 3·9)	7·0 (4·9 to 9·8)	11·4 (7·8 to 15·9)	1·7% (0·0 to 3·3)	8·4 (6·3 to 10·8)	32·7 (25·1 to 42·3)	4·7% (3·2 to 6·1)
		Malaysia	5·6 (4·0 to 7·6)	12·7 (9·0 to 17·3)	2·8% (1·2 to 4·4)	24·9 (18·4 to 32·7)	59·0 (42·5 to 78·6)	3·0% (1·5 to 4·4)	23·3 (18·6 to 29·6)	76·6 (59·3 to 98·1)	3·9% (2·5 to 5·3)
		Maldives	3·5 (2·4 to 5·0)	34·4 (24·2 to 47·5)	7·9% (6·2 to 9·6)	7·1 (4·9 to 9·9)	46·5 (32·9 to 63·2)	6·5% (5·0 to 8·1)	19·9 (14·9 to 26·1)	65·8 (51·3 to 84·9)	4·1% (2·8 to 5·6)
		Mauritius	6·0 (4·0 to 8·5)	16·5 (11·9 to 22·2)	3·5% (1·9 to 5·3)	9·2 (6·1 to 12·8)	19·8 (14·0 to 26·9)	2·7% (1·0 to 4·3)	23·3 (17·8 to 31·2)	83·7 (61·0 to 111·2)	4·2% (2·7 to 5·7)
		Myanmar	2·2 (1·5 to 3·1)	9·2 (6·3 to 12·8)	5·0% (3·2 to 6·6)	2·8 (1·9 to 3·9)	11·0 (7·6 to 15·2)	4·7% (3·1 to 6·4)	4·7 (3·6 to 5·7)	36·4 (27·6 to 47·0)	7·3% (5·7 to 8·7)
		Philippines	4·2 (3·2 to 5·4)	3·8 (2·9 to 4·8)	−0·4% (−1·6 to 1·0)	21·4 (16·7 to 27·1)	19·8 (16·0 to 24·3)	−0·2% (−1·4 to 0·9)	16·9 (13·7 to 20·1)	40·8 (34·9 to 48·3)	2·8% (1·9 to 3·9)
		Seychelles	7·6 (5·2 to 11·0)	12·9 (9·0 to 18·4)	1·8% (0·2 to 3·5)	24·2 (17·0 to 33·7)	42·6 (30·4 to 59·7)	1·9% (0·5 to 3·6)	40·6 (31·2 to 52·4)	99·3 (74·7 to 124·9)	2·6% (1·2 to 4·0)
		Sri Lanka	2·6 (1·8 to 3·7)	9·3 (6·6 to 13·2)	4·3% (2·7 to 6·0)	7·6 (5·2 to 10·4)	16·1 (11·4 to 22·1)	2·6% (1·1 to 4·3)	17·3 (12·9 to 23·2)	52·4 (38·6 to 70·1)	3·6% (2·2 to 5·0)
		Thailand	4·3 (2·9 to 6·0)	7·0 (4·8 to 9·8)	1·7% (0·1 to 3·3)	34·5 (25·1 to 47·7)	75·1 (53·0 to 102·4)	2·7% (1·3 to 4·1)	29·4 (23·4 to 36·1)	60·2 (46·9 to 75·6)	2·3% (0·8 to 3·7)
		Timor-Leste	2·2 (1·4 to 3·2)	5·3 (3·7 to 7·4)	3·1% (1·5 to 4·8)	4·5 (3·1 to 6·5)	8·8 (6·1 to 12·3)	2·3% (0·7 to 3·9)	3·3 (2·6 to 4·0)	12·5 (9·5 to 16·5)	4·4% (2·8 to 6·0)
		Vietnam	3·4 (2·5 to 4·7)	7·7 (5·2 to 10·8)	2·7% (1·1 to 4·4)	4·9 (3·5 to 6·7)	9·9 (6·9 to 14·1)	2·4% (0·8 to 4·2)	12·6 (9·3 to 16·2)	58·8 (44·0 to 76·0)	5·3% (4·0 to 6·9)
Sub-Saharan Africa*			**1·8 (1·3 to 2·6)**	**2·9 (2·1 to 4·0)**	**1·7% (−1·7 to 2·8)**	**10·6 (7·7 to 14·4)**	**18·3 (13·6 to 24·0)**	**1·9% (−0·5 to 3·0)**	**9·8 (7·8 to 12·1)**	**19·1 (15·4 to 23·4)**	**2·1% (−0·5 to 3·4)**
	Central sub-Saharan Africa		3·0 (2·0 to 4·4)	4·4 (3·0 to 6·1)	2·5% (−1·1 to 5·9)	22·1 (16·2 to 30·2)	35·9 (26·7 to 46·9)	2·0% (−1·2 to 5·0)	9·4 (6·7 to 11·5)	22·9 (18·5 to 27·9)	3·6% (0·5 to 7·7)
		Angola	1·3 (0·9 to 1·9)	4·6 (3·2 to 6·5)	4·3% (2·6 to 6·1)	16·8 (12·0 to 22·9)	16·3 (11·6 to 22·2)	−0·1% (−1·7 to 1·5)	11·7 (9·1 to 14·9)	26·9 (20·8 to 33·4)	3·1% (1·6 to 4·7)
		Central African Republic	1·9 (1·3 to 2·8)	2·5 (1·7 to 3·6)	1·0% (−0·8 to 2·7)	9·3 (6·5 to 13·0)	9·4 (6·4 to 13·5)	0·0% (−1·6 to 1·7)	11·6 (9·1 to 14·8)	16·9 (12·4 to 21·8)	1·3% (−0·2 to 2·7)
		Congo (Brazzaville)	5·7 (3·9 to 8·3)	5·7 (3·8 to 8·2)	0·0% (−1·8 to 1·6)	14·6 (10·1 to 20·4)	35·9 (24·9 to 49·9)	3·1% (1·5 to 4·7)	9·9 (7·9 to 11·7)	39·9 (28·7 to 51·7)	4·8% (3·2 to 6·3)
		Democratic Republic of the Congo	3·3 (2·2 to 4·9)	4·1 (2·8 to 5·7)	0·8% (−1·0 to 2·6)	24·7 (18·2 to 33·8)	43·8 (33·0 to 56·6)	2·0% (0·5 to 3·4)	8·4 (5·7 to 10·2)	18·6 (15·9 to 22·1)	2·9% (1·3 to 4·4)
		Equatorial Guinea	4·1 (2·8 to 5·7)	12·7 (8·5 to 18·4)	3·9% (2·3 to 5·6)	7·1 (4·9 to 10·1)	22·8 (15·3 to 32·4)	4·0% (2·3 to 5·7)	20·3 (14·6 to 26·8)	144·5 (107·4 to 186·9)	6·9% (5·5 to 8·3)
		Gabon	2·2 (1·4 to 3·2)	8·7 (6·0 to 12·4)	4·7% (3·0 to 6·4)	35·0 (24·9 to 47·5)	67·7 (49·0 to 92·9)	2·3% (0·8 to 3·7)	9·6 (7·3 to 12·9)	34·8 (24·7 to 39·2)	3·9% (2·3 to 5·4)
	Eastern sub-Saharan Africa		1·2 (0·8 to 1·7)	2·2 (1·6 to 3·0)	1·8% (−0·6 to 4·0)	6·6 (4·8 to 8·7)	13·8 (10·3 to 18·2)	1·9% (−0·4 to 6·8)	7·4 (6·1 to 9·0)	15·1 (12·1 to 18·6)	2·3% (−0·0 to 4·9)
		Burundi	0·7 (0·4 to 1·0)	1·1 (0·7 to 1·6)	1·7% (−0·1 to 3·6)	9·6 (6·9 to 13·1)	14·3 (10·3 to 19·7)	1·4% (−0·0 to 2·8)	5·6 (4·4 to 7·1)	7·4 (5·3 to 8·9)	0·9% (−0·6 to 2·5)
		Comoros	2·1 (1·5 to 3·0)	4·6 (3·2 to 6·5)	2·7% (0·9 to 4·4)	9·7 (6·7 to 13·5)	18·3 (12·8 to 25·7)	2·2% (0·6 to 3·8)	9·7 (7·6 to 13·1)	16·8 (12·7 to 21·2)	2·1% (0·5 to 3·6)
		Djibouti	1·4 (1·0 to 2·1)	2·8 (1·8 to 4·1)	2·3% (0·6 to 4·2)	7·5 (5·3 to 10·3)	8·7 (6·0 to 12·1)	0·5% (−1·1 to 2·2)	3·6 (3·0 to 4·5)	13·4 (10·3 to 16·7)	4·1% (2·5 to 5·7)
		Eritrea	0·7 (0·5 to 1·1)	0·8 (0·5 to 1·1)	0·2% (−1·5 to 2·1)	7·9 (5·5 to 11·1)	14·8 (10·6 to 20·3)	2·1% (0·6 to 3·8)	6·2 (4·9 to 7·5)	8·8 (6·5 to 10·8)	1·1% (−0·5 to 2·6)
		Ethiopia	0·4 (0·3 to 0·5)	0·8 (0·5 to 1·1)	2·0% (0·5 to 3·5)	1·0 (0·8 to 1·3)	6·9 (4·8 to 9·3)	6·5% (5·0 to 7·9)	4·7 (4·1 to 5·2)	9·2 (7·2 to 11·6)	2·8% (1·4 to 4·0)
		Kenya	2·1 (1·4 to 3·1)	3·3 (2·3 to 4·7)	1·6% (−0·3 to 3·3)	9·3 (6·4 to 13·2)	25·4 (18·1 to 35·2)	3·5% (1·9 to 5·1)	11·5 (8·6 to 14·5)	22·4 (16·6 to 28·1)	2·3% (0·8 to 3·9)
		Madagascar	3·0 (2·0 to 4·3)	4·6 (3·2 to 6·5)	1·5% (−0·1 to 3·2)	7·0 (4·8 to 9·9)	8·4 (5·7 to 11·9)	0·6% (−1·0 to 2·2)	12·2 (9·1 to 15·2)	19·8 (14·7 to 26·4)	1·8% (0·4 to 3·2)
		Malawi	0·4 (0·3 to 0·6)	0·9 (0·6 to 1·3)	2·7% (0·9 to 4·3)	6·8 (4·9 to 9·5)	13·0 (9·1 to 18·1)	2·2% (0·7 to 3·7)	6·0 (4·8 to 7·7)	18·6 (13·9 to 25·1)	4·1% (2·5 to 5·6)
		Mozambique	0·7 (0·5 to 0·9)	0·8 (0·6 to 1·1)	0·5% (−1·0 to 2·0)	3·4 (2·5 to 4·5)	9·4 (6·9 to 12·3)	3·5% (2·1 to 4·9)	7·7 (6·3 to 9·4)	12·9 (10·5 to 16·4)	1·8% (0·5 to 3·0)
		Rwanda	1·5 (1·1 to 2·2)	3·1 (2·4 to 4·1)	2·5% (0·8 to 4·1)	5·4 (3·9 to 7·3)	16·7 (13·3 to 20·6)	3·9% (2·7 to 5·2)	3·0 (2·5 to 3·3)	8·8 (7·5 to 10·7)	3·9% (2·5 to 5·3)
		Somalia	0·7 (0·4 to 1·0)	0·9 (0·6 to 1·2)	0·9% (−0·7 to 2·7)	2·4 (1·6 to 3·3)	3·3 (2·3 to 4·7)	1·1% (−0·6 to 2·8)	5·1 (3·8 to 6·8)	9·1 (7·0 to 12·0)	2·0% (0·4 to 3·4)
		South Sudan	1·2 (0·8 to 1·8)	1·6 (1·1 to 2·4)	1·0% (−0·8 to 2·9)	8·1 (5·6 to 11·4)	11·4 (7·9 to 16·2)	1·2% (−0·4 to 2·9)	6·7 (5·0 to 8·4)	9·3 (7·1 to 11·5)	0·9% (−0·6 to 2·6)
		Uganda	1·1 (0·9 to 1·5)	1·8 (1·3 to 2·5)	1·5% (0·0 to 2·8)	9·8 (8·4 to 11·3)	14·9 (11·3 to 19·4)	1·4% (0·3 to 2·5)	9·7 (8·9 to 10·7)	13·7 (11·1 to 16·7)	1·2% (0·1 to 2·3)
		Tanzania	1·9 (1·3 to 2·8)	4·9 (3·8 to 6·4)	3·3% (1·8 to 4·8)	12·2 (8·8 to 16·2)	22·0 (17·8 to 26·8)	2·0% (0·8 to 3·4)	8·0 (6·6 to 10·0)	23·3 (20·5 to 25·5)	3·0% (1·6 to 4·3)
		Zambia	1·0 (0·7 to 1·3)	1·9 (1·3 to 2·5)	2·3% (0·8 to 3·8)	14·3 (10·9 to 18·2)	21·8 (17·2 to 27·1)	1·5% (0·3 to 2·6)	9·4 (8·0 to 11·0)	22·9 (19·8 to 27·6)	3·0% (1·9 to 4·1)
	Southern sub-Saharan Africa		3·3 (2·4 to 4·6)	6·6 (5·2 to 8·4)	2·5% (−0·8 to 5·0)	20·1 (14·6 to 26·7)	33·5 (26·4 to 42·0)	2·6% (−0·2 to 5·3)	24·6 (19·9 to 30·5)	48·4 (41·2 to 55·3)	2·8% (−0·9 to 5·8)
		Botswana	2·2 (1·4 to 3·1)	5·4 (3·7 to 7·5)	3·2% (1·5 to 4·8)	13·6 (9·4 to 18·8)	46·5 (33·3 to 62·2)	4·2% (2·7 to 5·8)	12·2 (9·8 to 15·1)	54·5 (41·5 to 72·6)	4·7% (3·3 to 6·1)
		Eswatini	0·7 (0·4 to 1·0)	1·9 (1·3 to 2·8)	3·5% (1·6 to 5·5)	17·1 (12·0 to 23·6)	38·8 (27·6 to 52·0)	2·8% (1·3 to 4·3)	2·6 (1·9 to 3·0)	9·4 (8·6 to 12·2)	4·1% (2·3 to 5·8)
		Lesotho	0·8 (0·5 to 1·2)	1·0 (0·6 to 1·5)	0·7% (−1·1 to 2·4)	9·8 (6·7 to 13·6)	32·8 (22·9 to 44·8)	4·2% (2·6 to 5·7)	8·6 (6·7 to 11·2)	9·6 (8·4 to 11·0)	0·7% (−0·8 to 2·2)
		Namibia	1·8 (1·2 to 2·6)	5·1 (3·6 to 6·9)	3·6% (2·0 to 5·3)	13·4 (9·4 to 18·6)	24·3 (17·3 to 32·3)	2·1% (0·6 to 3·6)	7·6 (6·0 to 9·6)	27·7 (22·0 to 34·1)	4·3% (2·7 to 5·9)
		South Africa	4·1 (2·9 to 5·5)	8·3 (6·6 to 10·3)	2·5% (1·2 to 3·8)	20·0 (14·7 to 26·2)	34·6 (28·3 to 41·8)	1·9% (0·7 to 3·1)	30·0 (24·5 to 37·1)	60·5 (52·0 to 68·4)	2·2% (1·1 to 3·2)
		Zimbabwe	1·7 (1·1 to 2·6)	1·9 (1·3 to 2·8)	0·4% (−1·5 to 2·3)	24·4 (17·4 to 33·3)	28·6 (20·4 to 39·9)	0·5% (−0·9 to 2·0)	13·5 (10·0 to 17·0)	14·4 (11·4 to 17·1)	0·1% (−1·6 to 1·6)
	Western sub-Saharan Africa		1·7 (1·2 to 2·5)	2·5 (1·8 to 3·5)	1·3% (−3·0 to 4·5)	8·8 (6·2 to 12·2)	14·6 (10·6 to 19·5)	1·8% (−0·1 to 4·2)	8·3 (6·5 to 10·3)	16·5 (13·1 to 21·0)	1·5% (−0·6 to 5·2)
		Benin	0·8 (0·5 to 1·1)	1·3 (0·9 to 1·8)	1·8% (0·2 to 3·5)	5·7 (4·0 to 8·0)	7·8 (5·5 to 10·7)	1·0% (−0·5 to 2·7)	9·2 (7·0 to 12·5)	16·0 (12·3 to 20·8)	1·3% (−0·1 to 2·9)
		Burkina Faso	0·7 (0·4 to 1·0)	0·9 (0·6 to 1·3)	1·0% (−0·7 to 2·8)	6·8 (4·9 to 9·5)	11·2 (7·7 to 15·5)	1·7% (0·2 to 3·2)	5·0 (3·7 to 6·4)	6·9 (5·4 to 8·9)	0·9% (−0·6 to 2·3)
		Cameroon	2·3 (1·6 to 3·3)	3·0 (2·1 to 4·1)	0·9% (−1·0 to 2·6)	16·6 (11·8 to 22·8)	26·6 (19·2 to 35·8)	1·7% (0·1 to 3·2)	20·0 (16·0 to 25·4)	39·7 (30·9 to 50·0)	2·3% (0·8 to 3·7)
		Cape Verde	3·8 (2·6 to 5·4)	9·8 (6·6 to 13·3)	3·3% (1·7 to 5·0)	7·9 (5·6 to 10·7)	22·9 (16·2 to 31·6)	3·7% (2·2 to 5·3)	5·0 (3·8 to 6·3)	16·7 (13·0 to 20·2)	3·6% (2·1 to 5·1)
		Chad	0·8 (0·5 to 1·1)	1·0 (0·7 to 1·4)	0·9% (−0·8 to 2·8)	2·6 (1·7 to 3·8)	6·8 (4·6 to 9·4)	3·3% (1·6 to 5·0)	7·8 (5·8 to 10·1)	7·6 (5·9 to 9·7)	−0·1% (−1·8 to 1·5)
		Côte d'Ivoire	1·9 (1·2 to 2·7)	3·1 (2·1 to 4·4)	1·8% (0·1 to 3·6)	9·2 (6·5 to 13·0)	13·0 (9·3 to 17·8)	1·2% (−0·4 to 2·8)	9·6 (7·1 to 11·8)	15·1 (11·5 to 19·1)	1·1% (−0·5 to 2·5)
		The Gambia	1·3 (0·9 to 1·9)	1·4 (0·9 to 2·0)	0·1% (−1·8 to 1·9)	11·5 (8·0 to 15·6)	19·4 (14·3 to 27·1)	1·8% (0·3 to 3·3)	8·7 (6·8 to 12·0)	7·9 (5·6 to 8·8)	0·4% (−1·1 to 2·0)
		Ghana	1·8 (1·2 to 2·5)	1·7 (1·2 to 2·3)	−0·1% (−1·9 to 1·6)	13·0 (9·3 to 17·8)	30·8 (23·4 to 39·9)	3·0% (1·5 to 4·6)	8·5 (7·1 to 10·2)	17·4 (13·7 to 22·2)	1·5% (−0·0 to 3·0)
		Guinea	1·5 (1·0 to 2·1)	1·5 (1·0 to 2·1)	0·1% (−1·5 to 1·9)	3·1 (2·1 to 4·3)	5·4 (3·7 to 7·6)	1·9% (0·3 to 3·6)	3·6 (2·9 to 4·7)	6·9 (5·3 to 8·8)	2·2% (0·8 to 3·8)
		Guinea-Bissau	1·2 (0·8 to 1·7)	2·2 (1·5 to 3·1)	2·1% (0·4 to 4·0)	8·6 (6·2 to 11·8)	13·5 (9·6 to 18·3)	1·5% (−0·1 to 3·0)	4·8 (3·8 to 6·1)	9·0 (6·8 to 11·5)	1·7% (0·1 to 3·2)
		Liberia	0·5 (0·3 to 0·7)	1·2 (0·8 to 1·8)	3·3% (1·5 to 5·1)	5·8 (4·1 to 8·1)	13·2 (9·3 to 18·6)	2·8% (1·1 to 4·4)	5·6 (4·2 to 6·9)	22·6 (17·3 to 30·0)	5·1% (3·5 to 6·7)
		Mali	1·2 (0·8 to 1·8)	2·1 (1·4 to 3·0)	1·9% (0·3 to 3·6)	6·2 (4·4 to 8·7)	8·0 (5·3 to 11·0)	0·9% (−0·7 to 2·4)	5·3 (4·2 to 6·7)	9·3 (7·4 to 12·3)	1·7% (0·2 to 3·1)
		Mauritania	1·6 (1·0 to 2·3)	2·4 (1·6 to 3·4)	1·4% (−0·4 to 3·1)	10·2 (7·1 to 13·9)	17·3 (12·4 to 23·3)	1·8% (0·4 to 3·5)	4·5 (3·7 to 5·1)	6·9 (5·2 to 8·7)	0·8% (−0·9 to 2·4)
		Niger	0·6 (0·4 to 0·8)	0·9 (0·6 to 1·3)	1·7% (−0·1 to 3·4)	4·1 (2·8 to 5·8)	6·3 (4·3 to 8·7)	1·5% (−0·2 to 3·0)	5·6 (4·4 to 6·9)	8·2 (6·2 to 10·8)	1·0% (−0·5 to 2·4)
		Nigeria	2·1 (1·4 to 3·0)	3·3 (2·3 to 4·5)	1·6% (−0·2 to 3·3)	9·1 (6·4 to 12·6)	14·6 (10·7 to 19·2)	1·7% (0·1 to 3·1)	8·3 (6·5 to 9·9)	18·2 (14·7 to 23·0)	2·2% (0·8 to 3·7)
		São Tomé and Príncipe	2·7 (1·8 to 3·9)	1·2 (0·8 to 1·7)	−2·9% (−4·7 to −1·1)	15·0 (11·0 to 20·5)	22·9 (16·5 to 31·1)	1·5% (−0·1 to 2·9)	0·3 (0·0 to 0·7)	6·5 (4·5 to 8·6)	2·3% (0·7 to 3·9)
		Senegal	2·1 (1·4 to 3·0)	2·1 (1·4 to 2·9)	−0·0% (−1·7 to 1·7)	8·7 (6·2 to 11·6)	12·3 (8·9 to 16·8)	1·2% (−0·4 to 2·8)	5·2 (4·1 to 6·5)	6·5 (4·9 to 8·1)	0·7% (−0·7 to 2·2)
		Sierra Leone	0·6 (0·4 to 0·9)	1·9 (1·3 to 2·7)	3·9% (2·2 to 5·6)	5·7 (3·8 to 8·1)	10·4 (7·3 to 14·3)	2·1% (0·5 to 3·8)	12·4 (9·2 to 16·2)	18·8 (14·7 to 24·0)	0·9% (−0·5 to 2·2)
		Togo	1·1 (0·8 to 1·7)	1·4 (0·9 to 2·0)	0·6% (−1·3 to 2·4)	8·4 (5·8 to 11·9)	13·3 (9·3 to 18·3)	1·6% (−0·2 to 3·1)	9·0 (7·0 to 11·7)	17·5 (13·3 to 22·8)	1·7% (0·1 to 3·2)

Data in parentheses are 95% uncertainty intervals. GBD=Global Burden of Diseases, Injuries, and Risk Factors Study.

* Refers to GBD super-region.

The density of physicians increased globally between 1990 and 2019, with an annualised rate of change of 2·0% (95% UI −0·9 to 5·6). From 1990 to 2019, the GBD super-region encompassing north Africa and the Middle East had the largest annualised rate of change (increasing by 2·7% [0·7 to 5·5]), whereas the high-income super-region had the smallest annualised rate of change (increasing by 1·5% [–0·8 to 2·4]; table 1).

In comparison, the global density of nurses and midwives in 2019 was 38·6 (95% UI 30·1–48·8) per 10 000 population. A greater than ten-fold difference also existed in median nurse and midwife densities between the lowest and highest SDI quintiles (figure 1B). A large increase in this cadre was observed between the high-middle and high SDI countries. Across super-regions, densities ranged from 9·7 (7·3–12·8) per 10 000 in south Asia to 114·9 (94·7–137·7) per 10 000 population in the high-income super-region (table 1). Differences within super-regions were especially large in the high-income super-region, where a density of 152·3 (116·3–195·9) nurses and midwives per 10 000 population in the Australasia region contrasted with a density of 37·4 (30·2–46·3) per 10 000 population in southern Latin America in 2019. Notable differences at the national level existed within both well-resourced and poorly resourced regions. Japan, with a density of 119·2 (94·7–148·8) nurses and midwives per 10 000 population, contrasted with South Korea's density of 52·6 (40·7–67·4) per 10 000 population, Botswana's density of 46·5 (33·3–62·2) nurses and midwives per 10 000 population differed from Lesotho's density of 32·8 (22·9–44·8) per 10 000 population, and Bhutan had a density of 28·4 (20·1–39·3) nurses and midwives per 10 000 population compared to Pakistan's density of 8·3 (6·6–10·2) per 10 000 population.

The density of nurses increased globally between 1990 and 2019, with an annualised rate of change of 2·1% (95% UI −0·7 to 5·5) per 10 000 population. As with physicians, the largest annualised rate of change in nurse and midwife densities at the super-region level from 1990 to 2019 was in north Africa and the Middle East (3·0% [–0·3 to 5·5] per 10 000 population) and the smallest annualised rate was in the high-income super-region (1·4% [–0·8 to 2·4] per 10 000 population).

To achieve a UHC effective coverage of 80 out of 100 at the global level, the minimum required number of health workers per 10 000 population was 20·7 for physicians, 70·6 for nurses and midwives, 8·2 for dentistry personnel, and 9·4 for pharmaceutical personnel (table 2). By comparison, to achieve a UHC effective coverage of 90, the minimum numbers of health workers per 10 000 population was estimated to be 35·4 for physicians, 114·5 for nurses and midwives, 14·5 for dentistry personnel, and 15·8 for pharmaceutical personnel.

**Table 2:** Cadre-specific minimum density thresholds per 10 000 population for achieving UHC 80 and UHC 90

	**Threshold for UHC 80 (per 10 000)**	**Threshold for UHC 90 (per 10 000)**
Physicians	20·7	35·4
Nurses and midwives	70·6	114·5
Dentistry personnel	8·2	14·5
Pharmaceutical personnel	9·4	15·8

UHC=universal health coverage. UHC 80=achieving a performance target of 80 out of 100 on the UHC effective coverage index. UHC 90=achieving a performance target of 90 out of 100 on the UHC effective coverage index.

**Figure 3:**
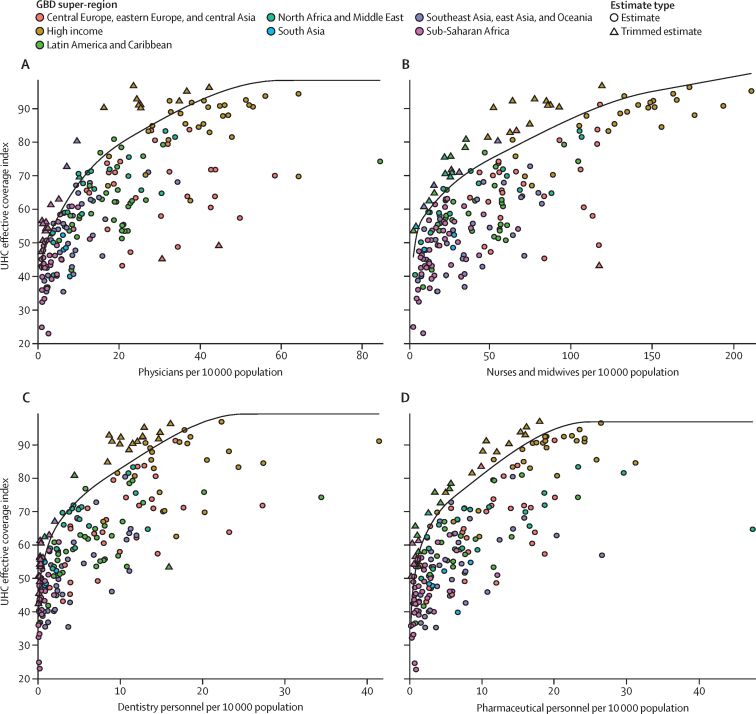
Maximum possible UHC effective coverage index achievement given densities in 2019 of physicians (A), nursing and midwifery personnel (B), dentistry personnel (C), and pharmaceutical personnel (D) Each point represents one out of 204 countries and territories. The solid black line refers to the frontier, which is the maximum expected UHC attainment at a given health worker density. The frontier was fit using all estimates from 1990 to 2017. This figure only shows the 2019 values for each location. GBD=Global Burden of Diseases, Injuries, and Risk Factors Study. UHC=universal health coverage.

In relation to a UHC effective coverage of 80 out of 100, in 2019, 132 of 204 countries and territories had workforce shortages for physicians, as did 154 countries and territories for nurses and midwives, 131 countries and territories for dentistry personnel, and 135 countries and territories for pharmaceutical personnel (appendix 2, table S2). In absolute terms, these corresponded to an aggregate shortage of approximately 6·4 million physicians, 30·6 million nurses and midwives, 3·3 million dentistry personnel, and 2·9 million pharmaceutical personnel globally (table 3). The HRH workforce gaps were larger and more concentrated among countries in the following GBD super-regions: sub-Saharan Africa, south Asia, and north Africa and the Middle East (figure 3). In terms of absolute shortages, the largest gaps were observed in sub-Saharan Africa (short by 1·9 million physicians, 5·6 million nurses and midwives, 824 000 dentistry personnel, and 856 0000 pharmaceutical personnel), southeast Asia, east Asia, and Oceania (short by 995 000 physicians, 8·8 million nurses and midwives, 745 000 dentistry personnel, and 560 000 pharmaceutical personnel), and south Asia (short by 2·6 million physicians, 11·0 million nurses and midwives, 1·3 million dentistry personnel, and 971 000 pharmaceutical personnel; table 3).

**Table 3:** Health worker shortages for four cadre groups at UHC effective coverage of 80 out of 100 on the UHC effective coverage index by GBD super-region, 2019

	**Number of countries with shortage**	Proportion of countries with shortage*(%)	**Sum of country-level shortages (number of workers)**
**Physicians (threshold: 20·7 per 10 000 per population)**			
Global	132	64·7%	6 410 000
Central Europe, eastern Europe, and central Asia	9	31·0%	25 000
High-income	3	8·3%	30 000
Latin America and Caribbean	22	66·7%	238 000
North Africa and Middle East	15	71·4%	636 000
South Asia	5	100·0%	2 570 000
Southeast Asia, east Asia, and Oceania	32	94·1%	995 000
Sub-Saharan Africa	46	100·0%	1 920 000
**Nurses and midwives (threshold: 70·6 per 10 000 population)**			
Global	154	75·5%	30 600 000
Central Europe, eastern Europe, and central Asia	17	58·6%	482 000
High-income	7	19·4%	348 000
Latin America and Caribbean	31	93·9%	1 570 000
North Africa and Middle East	18	85·7%	2 760 000
South Asia	5	100·0%	11 000 000
Southeast Asia, east Asia, and Oceania	30	88·2%	8 810 000
Sub-Saharan Africa	46	100·0%	5 640 000
**Dentistry personnel (threshold: 8·2 per 10 000 population)**			
Global	131	64·2%	3 280 000
Central Europe, eastern Europe, and central Asia	12	41·4%	38 500
High-income	1	2·8%	1300
Latin America and Caribbean	23	69·7%	32 800
North Africa and Middle East	17	81·0%	302 000
South Asia	5	100·0%	1 340 000
Southeast Asia, east Asia, and Oceania	27	79·4%	745 000
Sub-Saharan Africa	46	100·0%	824 000
**Pharmaceutical personnel (threshold: 9·4 per population)**			
Global	135	66·2%	2 890 000
Central Europe, eastern Europe, and central Asia	13	44·8%	52 200
High-income	4	11·1%	10 900
Latin America and Caribbean	28	84·8%	263 000
North Africa and Middle East	13	61·9%	182 000
South Asia	5	100·0%	971 000
Southeast Asia, east Asia, and Oceania	27	79·4%	560 000
Sub-Saharan Africa	45	97·8%	856 000

UHC=universal health coverage. GBD=Global Burden of Diseases, Injuries, and Risk Factors Study.

* The proportion of countries and territories within a super-region that exhibit a shortage.

## Discussion

Globally, HRH steadily increased between 1990 and 2019; yet, for all cadres, substantial differences persisted both within and across GBD super-regions. These differences translate into substantial health-worker shortages worldwide compared to estimated workforce levels necessary for achieving high levels of UHC effective coverage. Based on minimum threshold estimates for reaching a UHC effective coverage of 80 out of 100, national health workforce shortages in 2019 amounted to daunting totals: approximately 6·4 million physicians, 30·6 million nurses and midwives, 3·3 million dentistry personnel, and 2·9 million pharmaceutical personnel. Shortages in the GBD super-regions of sub-Saharan Africa and south Asia alone accounted for more than half of the global shortfall in each cadre; this finding aligns with shortage estimates published in recent WHO reports on nursing in 2020 and midwifery in 2021.^9^, ^10^

Minimum density thresholds represent a compromise between the ongoing demand from policy communities for standardised workforce benchmarks and the reality that considerable variation in skill mix undermines the utility of inflexible global targets. Rather than identifying ideal levels of HRH intended to pertain to all contexts, our density thresholds suggest a minimum health workforce common denominator; they represent the minimum human resources needed to achieve UHC performance goals. A goal of 80 out of 100 in the UHC effective coverage index reflects a high performance level that still falls within the spectrum of observed attainment among a diverse set of countries examined, making the corresponding thresholds broadly useful for health-system strengthening efforts.

Since our study fit workforce thresholds independently to each cadre, the minimum density value for a given HRH category is driven by countries that achieve high UHC with skill mixes that are relatively less reliant on that cadre. Other locations with skill mixes more reliant on a particular cadre are therefore likely to need workforce densities beyond these minimums. Consequently, these thresholds and their implied shortages should still apply to locations whose HRH skills mixes heavily favour certain cadres, including other allied health professionals such as community health workers.

Summing the minimum density thresholds calculated for physicians and for nurses and midwives at a UHC effective coverage index of 80 out of 100, the combined threshold is 91·3 per 10 000 population, more than double the WHO threshold of 44·5 for the aggregate of these same cadres.^31^ Another recent analysis similarly found that WHO methods might underestimate—by nearly double—the true scale of the midwife shortage.^44^ Unlike WHO's thresholds, ours are based on a more ambitious health-system performance target, and are primarily driven by locations with maximal, rather than median, translation of HRH to health coverage. As such, locations with lower productive efficiencies or additional challenges such as sparse population distribution might well need even larger numbers of health workers than those identified in these minimum thresholds.

We observed the greatest shortages in 2019 in densities of physicians, nurses and midwives, dentistry personnel, and pharmaceutical personnel in the super-regions of sub-Saharan Africa, south Asia, and north Africa and the Middle East. These areas contend with high rates of disease burden as well as expanding health-care needs due to the increasing prevalence of non-communicable diseases^40^ and due to population growth. Countries with rapidly growing populations and workforce shortages face a greater challenge. At the same time, these regions are home to countries and territories with some of the lowest indices of health-care access and quality, reflecting the clear association between adequate HRH densities and health service delivery. These workforce shortfalls might exist because of gaps in both supply and demand for health workers. Gaps in supply might be due to insufficient educational capacity. Limited demand for HRH can occur when there is insufficient employment capacity to absorb available workers. These dynamics are further exacerbated by a range of issues, including the out-migration of health workers, also known as a “brain drain”,^45^, ^46^ as well as absenteeism,^47^ wars and political unrest,^48^ violence against health workers,^49^, ^50^, ^51^ and insufficient financial and non-financial incentives to retain health workers.^52^ Efforts to scale up HRH will need to take into account the complex and varied causes of health worker shortages. WHO's Global Strategy on Human Resources for Health makes this clear.^16^ For example, it calls for different policy responses in locations with sizeable health worker out-migrations in contrast to locations with large in-migrations. And it emphasises that countries will need to address both the supply and the demand factors that produce gaps in HRH. This is a sizeable task that involves considering the scale and scope of training, education, and the broader workforce, as well as how far or close a country is to the minimum thresholds we estimate. Progressive realisation of UHC and the health workforce required to achieve UHC is a long-term effort. By doing a stochastic frontier analysis, we sought to improve UHC—in terms of both access and quality—by improving the allocation of resources. A realistic understanding of the gaps in UHC provides countries with a clearer picture of what is desirable, even though it might not always be possible to achieve. High-income locations can adopt responsible recruitment practices detailed in WHO's global code of practice on the international recruitment of health personnel to avoid further contributing to workforce gaps in GBD super-regions such as sub-Saharan Africa and south Asia.^53^ Responsible international recruitment will need to be coupled with appropriate workforce planning strategies to ensure domestic health-care needs are met.

Middle and low SDI locations seeking to increase HRH might continue to test and pursue retention strategies and incentives to reduce losses from out-migration.^54^, ^55^ The time and expenses involved in scaling up the training required for HRH means that expansion of educational opportunities can only be a long-term solution. Additionally, scaling up educational infrastructure alone will not help if large out-migrations of health-care personnel persist. In the shorter term, countries can direct funding towards expanding employment capacity.

Underlying most of these policy possibilities is the need to bolster health information systems that can better assess the size and composition of the workforce. The WHO National Health Workforce Accounts implementation guide^56^, ^57^ recommends multisectoral action to improve standardised data collection on health workforce characteristics.

Our finding that there is substantial variation in UHC effective coverage attainment at given levels of HRH suggests that increasing HRH should be just one element in a broader strategy to increase health coverage. Achieving UHC will require working conditions where health workers can thrive, boosting engagement, satisfaction, and ultimately workforce productivity. Other evidence-based strategies might include training physicians to work in rural locations,^58^ expanding public health programmes, and increasing access to essential medicines.^59^

Our analysis has a number of strengths. First, this study estimated health worker densities by use of standardised census and survey data and administrative or registry-based sources adjusted to be consistent with population-based sources. Adjustments were crucial to ensuring estimates were comparable across countries. Administrative and registry data rely on national health information systems that might omit workers in the private sector and double-count public sector workers with multiple positions. Furthermore, such sources do not adhere to a common process for classifying and collecting data on HRH cadres, which compromises comparability across locations. By using all possible data sources, our models included data from 96% of all 204 locations in our study.

Second, our approach to estimating the frontier of UHC effective coverage at a given level of HRH also has important advantages. Traditional stochastic frontier analysis requires specifying the functional form of the relationship between input and output. Our SFM approach avoided this requirement by fitting a production frontier with a flexible semi-parametric model. SFM also incorporated additional information on the uncertainty intervals of the dependent variable directly in the likelihood function to aid in frontier estimation. Additionally, including trimming within the likelihood prevented a small number of outliers from substantially shifting the frontier. This approach could be useful in other health-system performance or efficiency analyses. Future versions of the SFM model could include uncertainty in the fitted frontier and allow for flexible splines on more than one input variable, allowing direct estimation of substitution effects between cadres.

Third, we believe our new health workforce minimum threshold approach will be broadly useful for health-system strengthening efforts. We believe the thresholds for each cadre—physicians, nurses and midwives, pharmacists, and dentists—can be used to promote greater access to or better performance of the health system. We are not suggesting these minimum thresholds are compulsory, but rather aspirational. Each policy maker can take their own experience and use the threshold as a reference. Some locations have better UHC with fewer HRH, and vice versa; some with worse UHC have more HRH. The threshold is a convenient and innovative new metric for trying to determine the gaps. It is a not just a matter of quantity, but also quality.

This study has several limitations. First, some characteristics of our input data restricted our analysis. Some surveys had relatively small samples sizes for estimating the small prevalence values characteristic of health-worker densities in many places and times. This resulted in large sampling errors. These are not systematic sources of bias, however, because they are just as likely to result in overestimation as underestimation of a given indicator. For most data sources, the level of detail available in standard coding systems was also restricted, precluding disaggregation of some cadres into distinct professions (eg, community health workers and midwives) or by subspecialty (eg, specialist versus generalist physicians). Some of these limitations are inherent to even the most recent versions of such coding systems, whereas others reflect the preponderance of data coded to older versions of a system, such as ISCO-88. Mapping across coding systems and splitting aggregate codes during data preparation resulted in some loss of precision and conditioned the validity of estimates from less granular three-digit input data on the accuracy of available four-digit sources. The self-report nature of sources presents the potential for misclassifying occupations due to response bias or miscoding by interviewers. Our data sources also did not permit us to track whether health workers are employed in full-time or part-time positions, or whether they are professionals or associate professionals. The latter is an important limitation because the skills and competencies of associate professionals tend to be less advanced than those of professionals. Another limitation of the input data is the treatment of nurses and midwives as a single occupational grouping. Nursing and midwifery are separate disciplines that are not interchangeable and ideally require separate analysis. Additionally, available input data rarely provided information beyond the national level, precluding investigation of subnational heterogeneities in the supply and demand for HRH. Second, this study's frontier analyses did not account for potential substitution effects between cadres. In practice, roles and responsibilities among various cadres can overlap, particularly for task-shifting subgroups such as nurse practitioners. Consequently, the thresholds identified in this analysis probably underestimate true workforce requirements, because countries driving the frontier for one cadre might be compensating with higher densities in another. For instance, the low physician densities driving a frontier might only be possible with an unusually high density of nurses. In this way, our minimum density thresholds might collectively mask some workforce needs. Densities at or above the minimum threshold for any cadre might also mask deficits of specialists within that cadre grouping. It should also be noted that the UHC index does not include any particular input related to dentistry, although it might broadly represent better performance in dentistry correlated with the inputs. This is important when considering the minimum thresholds for dentistry. More precise estimates of the effective coverage of dentistry needs could improve the precision of minimum thresholds for dentistry.

Third, our analysis does not take into account some crucial characteristics of health workforces. For example, we do not currently produce estimates of the health workforce by age or sex. Analysing human resources for health in the context of gender is vital to discussions of economic development, equity advancement, and gender equity in health systems. We believe this topic both merits and requires a dedicated, separate analysis to appropriately address gender inequities in HRH and UHC. Such an analysis splitting health worker cadres by sex was outside the scope of the existing analysis but is a natural future extension of our research, as detailed below.

Last, we did not consider other important health workforce characteristics. Specifically, we did not examine variations in either the adequacy of workforce training or in workforce performance. Understanding both training and performance globally would require substantial improvements in country-level process-oriented data collection. Nor did we attempt to analyse the proportion of trained workforces that is unemployed, employed in non-health occupations, or that has emigrated from the country. Information on the prevalence of unemployment, non-health employment, and out-migration among workers with health-care training could, however, provide crucial insight into the mechanisms underlying low workforce densities and the relative potential of efforts to expand workforces by increasing supply and training as opposed to demand and retention.^60^ Our data sources did not allow us to assess either unpaid informal care providers, such as family members, or temporary health workers, such as international humanitarian workers. Regarding the contexts in which health workers practice, our global thresholds are not sensitive to differences in national disease burdens or to varying population densities and distributions, both of which are likely to affect required workforce levels.

This study suggests several avenues for future research. First, further research should examine key characteristics of health workforces. Research should recognise additional cadres that contribute to UHC attainment across locations and extend the threshold analysis accordingly. Understanding the contributions of specialists, such as obstetricians, paediatricians, and surgeons, is another essential avenue. Work is also needed to quantify when surpluses in some cadres can compensate for deficits in others. Such research could identify the specific contextual factors that make training community health workers and shifting health-care tasks better options for expanding UHC than attempting to increase the density of physicians or of other cadres traditionally emphasised in global policy dialogue.

Second, further study of health workforce composition is warranted. Disaggregating HRH densities by sex and examining differences in the sex distribution among and within cadres is crucial for examining the gendered nature of health work. To provide effective coverage, health systems explicitly rely on women's paid labour. Notably, nurses and midwives comprise the largest health worker cadre globally, and in some countries more than 90% of nurses and midwives are women.^61^ In the paid workforce, underemployment, unemployment, and labour wastage continue to be gendered phenomena that disadvantage female health workers.^62^ Additionally, the provision of effective health care implicitly relies on unpaid labour. Results from the Global Valuing the Invaluable analysis indicate that unpaid labour accounts for 31–49% of women's total contribution to the health sector, depending on the valuation method,^61^ and women contribute a disproportionate amount of informal, unpaid labour to the health sector compared to men due to domestic caregiving norms.^63^ Systematic gender-based discrimination affects female health workers' paid and unpaid labour, and future research must examine gender differences in the health workforce to empower health workers and promote initiatives that improve gender equity.

Third, the threshold analysis could account for other population health needs and health system goals. For instance, as more countries obtain higher levels of UHC, it will be possible to establish reliable minimum thresholds of HRH with respect to even higher targets of UHC effective coverage. Some work has also assessed HRH densities in relation to disease and injury burden.^63^ Such research could help societies avert health loss, particularly from non-communicable diseases that are on the rise globally. Information on the availability of gerontologists could help societies prepare to care for ageing populations, for instance, and understanding the prevalence of psychologists, psychiatrists, and other mental health professionals could facilitate efforts to address the global burden of depression and suicide. Research on how the size and composition of the health workforce affect pandemic preparedness is also clearly of paramount importance. The 2014 west Africa Ebola virus outbreak and the more recent spread of this disease in the Democratic Republic of the Congo showed how shortfalls in HRH bear not only on UHC but also on global health security more generally.^64^, ^65^, ^66^ The COVID-19 pandemic highlighted the crucial importance of addressing these shortfalls for disaster responses and health-system resilience.^67^

Fourth, additional research above and below the national level would be fruitful. Analyses by region or type of health system could yield more precise HRH targets by taking into account prevalent skill mixes. More granular research is also important because national-level estimates could mask considerable subnational disparities and shortages in health workers and health outcomes. Previous work has highlighted how HRH tend to concentrate in urban areas,^68^ leaving shortfalls in rural and remote areas that could be rectified through national attention and policies.

Fifth, there is substantial opportunity for investigating how total national health expenditure^69^ corresponds to the gaps and shortfalls in HRH documented here. In many countries, human resources make up a major part of health sector expenditures, and understanding how these resources are balanced against other demands, such as capital investments in buildings and equipment, as well as drugs and devices, is crucial. Similarly, the allocation of expenditure for HRH is crucial, as investments in different levels of HRH will have different ramifications for both the amount of resources spent as well as the care that can be provided.

Finally, future research could build on existing forecasts of future workforce needs.^9^, ^10^, ^52^ Forecasts could incorporate trends in migration, technology, health financing, and health worker training capacity. This would enable decision makers facing resource scarcities to make timely investments in training and recruitment in anticipation of future scenarios.

A strong health workforce is recognised as being crucial to a range of policy priorities, yet HRH estimates across countries show there are considerable disparities in HRH. This analysis illuminated widespread shortages in HRH whose elimination will be necessary—albeit insufficient on its own—in global efforts to achieve effective UHC for all people. As the WHO Global Strategy on Human Resources for Health^16^ suggests, successful policy solutions will vary across contexts to address the local drivers of insufficient workforce supply and demand. Taking these diverse factors seriously is important not only to extending effective health-care coverage in the present, but also to ensuring global health security in the future.

## Data sharing

To download the data used in these analyses, please visit the Global Health Data Exchange GBD 2019 results website.

## Declaration of interests

T W Bärnighausen reports research grants from the European Union (Horizon 2020 and EIT Health), German Research Foundation (DFG), US National Institutes of Health, German Ministry of Education and Research, Alexander von Humboldt Foundation, Else-Kröner-Fresenius-Foundation, Wellcome Trust, Bill & Melinda Gates Foundation, KfW, The Joint United Nations Programme on HIV/AIDS (UNAIDS), and WHO; consulting fees from KfW for consultancy on the OSCAR initiative in Vietnam; participation on a data safety monitoring board or advisory board with the NIH-funded study “Healthy Options” (PIs: Smith Fawzi, Kaaya) as Chair, Data Safety and Monitoring Board (DSMB), German National Committee on the “Future of Public Health Research and Education”, Chair of the scientific advisory board to the EDCTP Evaluation, membership of the UNAIDS Evaluation Expert Advisory Committee, National Institutes of Health Study Section Member on Population and Public Health Approaches to HIV/AIDS (PPAH), US National Academies of Sciences, Engineering, and Medicine's Committee for the “Evaluation of Human Resources for Health in the Republic of Rwanda under the President's Emergency Plan for AIDS Relief (PEPFAR)”, University of Pennsylvania (UPenn) Population Aging Research Center (PARC) as an external advisory board member; and a leadership or fiduciary role in a board, society, committee, or advocacy group, paid or unpaid, with Global Health Hub Germany (which was initiated by the German Ministry of Health) as co-Chair; all outside the submitted work. B Bikbov reports grants from Lombardy Region, paid to the Istituto di Ricerche Farmacologiche Mario Negri IRCCS; support for attending meetings or travel, or both, from the European Commission; all outside the submitted work. N Fullman reports other funding support from WHO as a consultant from June to September, 2019, and Gates Ventures since 2020, all outside the submitted work. N J Henry reports grants or contracts from the Bill & Melinda Gates Foundation, outside the submitted work. S M S Islam reports grants or contracts from the National Health and Medical Research Council of Australia via the Emerging Leadership Fellowship, outside the submitted work. K Krishan reports other non-financial support from the UGC Centre of Advanced Study, CAS II, Department of Anthropology, Panjab University, Chandigarh, India, outside the submitted work. J L Leasher reports a leadership or fiduciary role in a board, society, committee, or advocacy group, paid or unpaid with Planning Group Member for the National Eye Health Education Program, outside the submitted work. V C F Pepito reports grants or contracts from Sanofi Consumer Healthcare received as payments to his institution to do research on self-care, and from the International Initiative for Impact Evaluation received as payments to his institution to conduct evaluations on PhilHealth; all outside the submitted work. D M Pigott reports grants from the Bill & Melinda Gates Foundation, outside the submitted work. All other authors declare no competing interests.
