# Supplementary material for: Measuring the availability of human resources for health and its relationship to universal health coverage for 204 countries and territories from 1990 to 2019: a systematic analysis for the Global Burden of Disease Study 2019
Source: Lancet. 2022 Jun 4;399(10341):2129–54. doi: 10.1016/S0140-6736(22)00532-3 (PMC9168805; doi:10.1016/S0140-6736(22)00532-3)
Supplement: Supplementary appendix 1 [file mmc1.pdf]

THE LANCET

Supplementary appendix 1

This appendix formed part of the original submission and has been peer reviewed. We post it as supplied by the authors.

Supplement to: GBD 2019 Human Resources for Health Collaborators. Measuring the availability of human resources for health and its relationship to universal health coverage for 204 countries and territories from 1990 to 2019: a systematic analysis for the Global Burden of Disease Study 2019. *Lancet* 2022; published online May 23. https://doi.org/10.1016/S0140-6736(22)00532-3.

Appendix to Measuring the availability of human resources for health and its relationship to universal health coverage: estimates for 204 countries and territories from 1990 to 2019

This appendix provides further methodological detail and supplemental figures and tables.

Tables of contents

Preamble 2

List of appendix figures and tables 3

GATHER statement 4

Author contributions 5

GATHER checklist 7

Methods 9

Overview 9

Section 1. Estimating human resources for health 9

Section 2. Estimating the relationship between health worker densities and universal health coverage 29

Section 3. Online tools and glossary of terms 33

References 34

Preamble

This appendix provides methodological detail for estimating health worker densities, estimating minimum health worker density thresholds to achieve universal health coverage, and supplementary results. This study complies with the Guidelines for Accurate and Transparent Health Estimates Reporting (GATHER) recommendations.[1] It includes detailed modelling write-ups and information on data sourcing to maximise transparency in our estimation processes and provide a comprehensive account of analytical steps. Many of the methods outlined in this appendix have been described in previous GBD publications.[2,3] Portions of this appendix have been reproduced or adapted from Lozano[2] and the GBD 2019 Universal Health Coverage Collaborators.[3]

List of appendix figures and tables

Table 1: GATHER checklist
Figure 1: Flowchart of estimation process
Figure 2a: Country-years of occupation data for physicians, by country and territory, 1990-2019
Figure 2b: Country-years of occupation data for nurses and midwives, by country and territory, 1990-2019
Figure 2c: Country-years of occupation data for dentists, by country and territory, 1990-2019
Figure 2d: Country-years of occupation data for pharmacists, by country and territory, 1990-2019
Table 2: Input sources by coding system and level of granularity
Table 3. Input and output data source counts by adjustment status and data type
Table 4: Cadres and corresponding occupation codes
Figure 3: Number of sources by coding system, 1980-2019
Figure 4a: Example subsection of the ISCO 88 hierarchy
Figure 4b: Example subsection of the ISCO 08 hierarchy
Table 5: Examples of health worker cadre mapping across ISCO versions
Figure 5a: Matched pairs of IHME population-based points and WHO data points for physicians, 1990 - 2019
Figure 5b: Matched pairs of IHME population-based points and WHO data points for nurses and midwives, 1990 - 2019
Figure 6a: Matched pairs of IHME population-based points and WHO data points for physicians, 1990 – 2019
Figure 6b: Matched pairs of IHME population-based points and WHO data points for nurses and midwives, 1990 - 2019
Figure 6c: Matched pairs of IHME population-based points and WHO data points for dentists, 1990 - 2019
Figure 6d: Matched pairs of IHME population-based points and WHO data points for pharmacists, 1990 - 2019
Figure 7a: Average percent difference between IHME population-based data and WHO data for dentists
Figure 7b: Average percent difference between IHME population-based data and WHO data for dentists
Figure 7c: Average percent difference between IHME population-based data and WHO data for dentists
Figure 7d: Average percent difference between IHME population-based data and WHO data for dentists
Table 6: Average percent difference between IHME population-based data and WHO data for all cadres
Figure 8a: Adjustment type for physicians, WHO data
Figure 8b: Adjustment type for nurses and midwives, WHO data
Figure 8c: Adjustment type for dentists, WHO data
Figure 8d: Adjustment type for pharmacists, WHO data
Table 7: Lambdas and RMSE from the lasso covariate selection regressions, by cadre
Figure 9a: Average percent adjustment to the WHO data input data, physicians
Figure 9b: Average percent adjustment to the WHO data input data, nurses and midwives
Figure 9c: Average percent adjustment to the WHO data input data, pharmacists
Figure 9d: Average percent adjustment to the WHO data input data, dentists
Table 8: Covariates for ST-GPR models by cadre

GATHER statement

This study complies with the Guidelines for Accurate and Transparent Health Estimates Reporting (GATHER) recommendations.[1] We have documented the steps involved in our analytical procedures and detailed the data sources used in compliance with GATHER. For additional GATHER reporting, please refer to appendix table 1 on pages 8-9.

Author contributions

Managing the estimation process

Annie Haakenstad, Kelly Bienhoff, Kate E LeGrand, Stephen S Lim, Christopher J L Murray, and Rafael Lozano.

Writing the first draft of the manuscript

Caleb Mackay Salpeter Irvine, Megan Knight, Vin Gupta, and Rafael Lozano.

Primary responsibility for applying analytical methods to produce estimates

Annie Haakenstad, Caleb Mackay Salpeter Irvine, Megan Knight, Aleksandr Y Aravkin, Peng Zheng, Stephen S Lim, Christopher J L Murray, and Rafael Lozano.

Primary responsibility for seeking, cataloguing, extracting, or cleaning data; designing or coding figures and tables

Caleb Mackay Salpeter Irvine and Megan Knight.

Providing data or critical feedback on data sources

Annie Haakenstad, Michael R M Abrigo, Abdelrahman I Abushouk, Oladimeji M Adebayo, Fares Alahdab, Turki M Alanzi, Jacqueline Elizabeth Alcalde-Rabanal, Nelson Alvis-Guzman, Catalina Liliana Andrei, Tudorel Andrei, Carl Abelardo T Antonio, Jalal Arabloo, Olatunde Aremu, Martin Amogre Ayanore, Maciej Banach, Till Winfried Bärnighausen, Adam E Berman, Boris Bikbov, Archith Boloor, Luis Alberto Cámera, Soosanna Kumary Chattu, Vijay Kumar Chattu, Dinh-Toi Chu, Lalit Dandona, Rakhi Dandona, William James Dangel, Ahmad Daryani, Meghnath Dhimal, Hoa Thi Do, Leila Doshmangir, Elham Ehsani-Chimeh, Artem Alekseevich Fomenkov, Takeshi Fukumoto, Mohamed M Gad, Mansour Ghafourifard, Ahmad Ghashghaee, Rajat Das Gupta, Josep Maria Haro, Edris Hasanpoor, Mohamed I Hegazy, Mowafa Househ, Seyed Sina Naghibi Irvani, Sheikh Mohammed Shariful Islam, Mohammad Ali Jahani, Rohollah Kalhor, Gbenga A Kayode, Nauman Khalid, Khaled Khatab, Adnan Kisa, Sonali Kochhar, Kewal Krishan, Barthelemy Kuate Defo, Dharmesh Kumar Lal, Lee-Ling Lim, Narayan B Mahotra, Erkin M Mirrakhimov, Shafiu Mohammed, Ali H Mokdad, Mohsen Naghavi, Duduzile Edith Ndwandwe, Ionut Negoi, Ruxandra Irina Negoi, Josephine W Ngunjiri, Cuong Tat Nguyen, Obinna E Onwujekwe, Doris V Ortega-Altamirano, Mayowa O Owolabi, Veincent Christian Filipino Pepito, Hai Quang Pham, David M Pigott, Khem Narayan Pokhrel, Mohammad Rabiee, Navid Rabiee, Vafa Rahimi-Movaghar, Lal Rawal, Andre M N Renzaho, Serge Resnikoff, Nima Rezaei, Jennifer Rickard, Leonardo Roever, Abdallah M Samy, Juan Sanabria, Milena M Santric-Milicevic, Sivan Yegnanarayana Iyer Saraswathy, Subramanian Senthilkumaran, Edson Serván-Mori, Masood Ali Shaikh, Diego Augusto Santos Silva, Mariya Vladimirovna Titova, Stephanie M Topp, Marcos Roberto Tovani-Palone, Saif Ullah, Bhaskaran Unnikrishnan, Pascual R Valdez, Narayanaswamy Venketasubramanian, Vasily Vlassov, Naohiro Yonemoto, Mustafa Z Younis, Chuanhua Yu, Siddhesh Zadey, Arash Ziapour, and Rafael Lozano.

Developing methods or computational machinery

Annie Haakenstad, Aleksandr Y Aravkin, Peng Zheng, Xiaochen Dai, Ahmad Daryani, Mowafa Househ, Adnan Kisa, Ali H Mokdad, Mohsen Naghavi, Abdallah M Samy, Arash Ziapour, and Rafael Lozano.

Providing critical feedback on methods or results

Annie Haakenstad, Vin Gupta, Michael R M Abrigo, Abdelrahman I Abushouk, Oladimeji M Adebayo, Gina Agarwal, Fares Alahdab, Ziyad Al-Aly, Khurshid Alam, Turki M Alanzi, Jacqueline Elizabeth Alcalde-Rabanal, Vahid Alipour, Nelson Alvis-Guzman, Arianna Maever L Amit, Catalina Liliana Andrei, Tudorel Andrei, Jalal Arabloo, Olatunde Aremu, Martin Amogre Ayanore, Maciej Banach, Till Winfried Bärnighausen, Mohsen Bayati, Habib Benzian, Adam E Berman, Ali Bijani, Boris Bikbov, Archith Boloor, Reinhard Busse, Zahid A Butt, Luis Alberto Cámera, Ismael R Campos-Nonato, Rosario Cárdenas, Collins Chansa, Soosanna Kumary Chattu, Vijay

Kumar Chattu, Dinh-Toi Chu, Xiaochen Dai, Lalit Dandona, Rakhi Dandona, Ahmad Daryani, Jan-Walter De Neve, Meghnath Dhimal, Shirin Djalalinia, Hoa Thi Do, Leila Doshmangir, Elham Ehsani-Chimeh, Florian Fischer, Nataliya A Foigt, Artem Alekseevich Fomenkov, Masoud Foroutan, Takeshi Fukumoto, Nancy Fullman, Mohamed M Gad, Keyghobad Ghadiri, Mansour Ghafourifard, Ahmad Ghashghaee, Houman Goudarzi, Rajat Das Gupta, Randah R Hamadeh, Samer Hamidi, Josep Maria Haro, Edris Hasanpoor, Simon I Hay, Mohamed I Hegazy, Behzad Heibati, Nathaniel J Henry, Naznin Hossain, Mowafa Househ, Olayinka Stephen Ilesanmi, Mohammad-Hasan Imani-Nasab, Seyed Sina Naghibi Irvani, Sheikh Mohammed Shariful Islam, Mohammad Ali Jahani, Ankur Joshi, Rohollah Kalhor, Gbenga A Kayode, Nauman Khalid, Khaled Khatab, Adnan Kisa, Sonali Kochhar, Kewal Krishan, Barthelemy Kuate Defo, Dharmesh Kumar Lal, Faris Hasan Lami, Anders O Larsson, Janet L Leasher, Lee-Ling Lim, Azeem Majeed, Narayana Manjunatha, Benjamin Ballard Massenburg, Andreea Mirica, Erkin M Mirrakhimov, Yousef Mohammad, Shafiu Mohammed, Ali H Mokdad, Mohsen Naghavi, Ionut Negoi, Ruxandra Irina Negoi, Josephine W Ngunjiri, Cuong Tat Nguyen, Yeshambel T Nigatu, Obinna E Onwujekwe, Doris V Ortega-Altamirano, Nikita Otstavnov, Stanislav S Otstavnov, Mayowa O Owolabi, Abhijit P Pakhare, Veincent Christian Filipino Pepito, Hai Quang Pham, David M Pigott, Khem Narayan Pokhrel, Vafa Rahimi-Movaghar, David Laith Rawaf, Salman Rawaf, Lal Rawal, Andre M N Renzaho, Serge Resnikoff, Nima Rezaei, Aziz Rezapour, Jennifer Rickard, Leonardo Roever, Maitreyi Sahu, Abdallah M Samy, Juan Sanabria, Milena M Santric-Milicevic, Sivan Yegnanarayana Iyer Saraswathy, Soraya Seedat, Subramanian Senthilkumaran, Edson Serván-Mori, Masood Ali Shaikh, Aziz Sheikh, Mariya Vladimirovna Titova, Stephanie M Topp, Marcos Roberto Tovani-Palone, Saif Ullah, Bhaskaran Unnikrishnan, Pascual R Valdez, Tommi Juhani Vasankari, Narayanaswamy Venketasubramanian, Theo Vos, Jamal Akeem Yearwood, Naohiro Yonemoto, Mustafa Z Younis, Chuanhua Yu, Siddhesh Zadey, Sojib Bin Zaman, Taddese Alemu Zerfu, Zhi-Jiang Zhang, Arash Ziapour, Sanjay Zodpey, Stephen S Lim, and Rafael Lozano.

Drafting the work or revising it critically for important intellectual content

Annie Haakenstad, Caleb Mackay Salpeter Irvine, Megan Knight, Corinne Bintz, Abdelrahman I Abushouk, Oladimeji M Adebayo, Gina Agarwal, Fares Alahdab, Khurshid Alam, Jacqueline Elizabeth Alcalde-Rabanal, Nelson Alvis-Guzman, Carl Abelardo T Antonio, Jalal Arabloo, Olatunde Aremu, Martin Amogre Ayanore, Maciej Banach, Till Winfried Bärnighausen, Habib Benzian, Adam E Berman, Kelly Bienhoff, Boris Bikbov, Antonio Biondi, Ismael R Campos-Nonato, Felix Carvalho, Soosanna Kumary Chattu, Vijay Kumar Chattu, Dinh-Toi Chu, Ahmad Daryani, Jan-Walter De Neve, Isaac Oluwafemi Dipeolu, Hoa Thi Do, Chirag P Doshi, Leila Doshmangir, Elham Ehsani-Chimeh, Maha El Tantawi, Eduarda Fernandes, Florian Fischer, Nataliya A Foigt, Masoud Foroutan, Takeshi Fukumoto, Mohamed M Gad, Mansour Ghafourifard, Ahmad Ghashghaee, Thomas Glucksman, Houman Goudarzi, Rajat Das Gupta, Randah R Hamadeh, Josep Maria Haro, Simon I Hay, Michael K Hole, Naznin Hossain, Mowafa Househ, Olayinka Stephen Ilesanmi, Mohammad-Hasan Imani-Nasab, Seyed Sina Naghibi Irvani, Sheikh Mohammed Shariful Islam, Gbenga A Kayode, Khaled Khatab, Adnan Kisa, Sonali Kochhar, Kewal Krishan, Barthelemy Kuate Defo, Anders O Larsson, Janet L Leasher, Kate E LeGrand, Lee-Ling Lim, Azeem Majeed, Afshin Maleki, Narayana Manjunatha, Benjamin Ballard Massenburg, Tomislav Mestrovic, GK Mini, Yousef Mohammad, Shafiu Mohammed, Ali H Mokdad, Shane Douglas Morrison, Mohsen Naghavi, Ionut Negoi, Ruxandra Irina Negoi, Josephine W Ngunjiri, Cuong Tat Nguyen, Obinna E Onwujekwe, Doris V Ortega-Altamirano, Nikita Otstavnov, Stanislav S Otstavnov, Mayowa O Owolabi, Veincent Christian Filipino Pepito, Norberto Perico, Hai Quang Pham, David M Pigott, Khem Narayan Pokhrel, Mohammad Rabiee, Navid Rabiee, Vafa Rahimi-Movaghar, David Laith Rawaf, Salman Rawaf, Lal Rawal, Giuseppe Remuzzi, Andre M N Renzaho, Nima Rezaei, Jennifer Rickard, Leonardo Roever, Maitreyi Sahu, Abdallah M Samy, Juan Sanabria, Milena M Santric-Milicevic, Diego Augusto Santos Silva, Caroline Stein, Dan J Stein, Marcos Roberto Tovani-Palone, Bhaskaran Unnikrishnan, Marco Vacante, Tommi Juhani Vasankari, Narayanaswamy Venketasubramanian, Vasily Vlassov, Naohiro Yonemoto, Sojib Bin Zaman, Taddese Alemu Zerfu, Arash Ziapour, and Rafael Lozano.

Managing the estimation or publications process

Annie Haakenstad, Kelly Bienhoff, Celine Barthelemy, William James Dangel, Simon I Hay, Ali H Mokdad, Mohsen Naghavi, and Rafael Lozano.

Table 1. GATHER checklist

GATHER checklist of information that should be included in reports of global health estimates, with description of compliance and location of information

#	GATHER checklist item	Description of compliance	Reference
**Objectives and funding**			
1	Define the indicators, populations, and time periods for which estimates were made.	Description of indicators, definitions, relevant time periods, and populations in paper and appendix.	Main text; Appendix, Sections 1-2
2	List the funding sources for the work.	Funding sources listed in paper.	Main text Summary
**Data inputs**			
For all data inputs from multiple sources that are synthesized as part of the study:			
3	Describe how the data were identified and how the data were accessed.	Narrative description of data-seeking methodology provided.	Main text Methods; Appendix Sections 1-2
4	Specify the inclusion and exclusion criteria. Identify all ad-hoc exclusions.	Narrative about inclusion and exclusion criteria by data type provided.	Main text Methods; Appendix Sections 1-2
5	Provide information on all included data sources and their main characteristics. For each data source used, report reference information or contact name/institution, population represented, data collection method, year(s) of data collection, sex and age range, diagnostic criteria or measurement method, and sample size, as relevant.	An interactive, online data source tool that provides metadata for data sources by geography and time.	Appendix Section 3<br><br>Online data tools will be updated at time of publication and findable at this link: http://ghdx.healthdata.org/gbd-2019
6	Identify and describe any categories of input data that have potentially important biases (eg, based on characteristics listed in item 5).	Summary of some known biases included in paper.	Main text Methods and Discussion
For data inputs that contribute to the analysis but were not synthesized as part of the study:			
7	Describe and give sources for any other data inputs.	An interactive, online data source tool that provides metadata for data sources.	Appendix Section 3<br><br>Online data tools will be updated at time of publication and findable at this link: http://ghdx.healthdata.org/gbd-2019
For all data inputs:			
8	Provide all data inputs in a file format from which data can be efficiently extracted (eg, a spreadsheet as opposed to a PDF), including all relevant meta-data listed in item 5. For any data inputs that cannot be shared due to ethical or legal reasons, such as third-party ownership, provide a contact name or the name of the institution that retains the right to the data.	Downloads of input data are available through online tools, including data visualisation tools and data query tools.	Appendix Section 3<br><br>Online data tools will be updated at time of publication and findable at this link: http://ghdx.healthdata.org/gbd-2019
**Data analysis**			

9	Provide a conceptual overview of the data analysis method. A diagram may be helpful.	Flow diagrams of the overall methodological processes provided.	Appendix Section 1
10	Provide a detailed description of all steps of the analysis, including mathematical formulae. This description should cover, as relevant, data cleaning, data pre-processing, data adjustments and weighting of data sources, and mathematical or statistical model(s).	Flow diagrams and corresponding methodological write-ups and modelling processes have been provided.	Main text Methods; Appendix Sections 1-2
11	Describe how candidate models were evaluated and how the final model(s) were selected.	Provided in the methodological write-ups.	Appendix Sections 1-2
12	Provide the results of an evaluation of model performance, if done, as well as the results of any relevant sensitivity analysis.	Provided the results of an evaluation of model performance.	Appendix Section 1
13	Describe methods for calculating uncertainty of the estimates. State which sources of uncertainty were, and were not, accounted for in the uncertainty analysis.	Cited source that describes method for calculating uncertainty of estimates. We did not account for uncertainty in the frontier analysis.	Included citation in main text and Appendix for information on uncertainty with respect to ST-GPR. Method for calculating uncertainty also referenced in Appendix Section 1.
14	State how analytic or statistical source code used to generate estimates can be accessed.	Access statement provided.	Links to code will be updated at time of publication and can be found here: http://ghdx.healthdata.org/gbd-2019
**Results and discussion**			
15	Provide published estimates in a file format from which data can be efficiently extracted.	GBD 2019 results are available through online data visualisation tools, the Global Health Data Exchange, and the online data query tool.	Online data tools will be updated at time of publication and findable at this link: http://ghdx.healthdata.org/gbd-2019
16	Report a quantitative measure of the uncertainty of the estimates (eg, uncertainty intervals).	Uncertainty intervals are provided with all results, except for the frontier analysis and corresponding thresholds and shortages.	Main text results, Main text table 1, Appendix table 1
17	Interpret results in light of existing evidence. If updating a previous set of estimates, describe the reasons for changes in estimates.		Summary; Research in Context; Main text
18	Discuss limitations of the estimates. Include a discussion of any modelling assumptions or data limitations that affect interpretation of the estimates.	Discussion of limitations provided in the narrative of the main paper.	Main text

Methods

Overview

Many of the methods described below were reported in previous Global Burden of Diseases, Injuries, and Risk Factors Study (GBD) publications.[2–4] Analyses were conducted with R version 4.0.3, Python version 2.7.5, or Stata version 15.1, and figures were generated with R version 4.0.3.

Section 1. Estimating human resources for health

Figure 1. Flowchart of estimation process

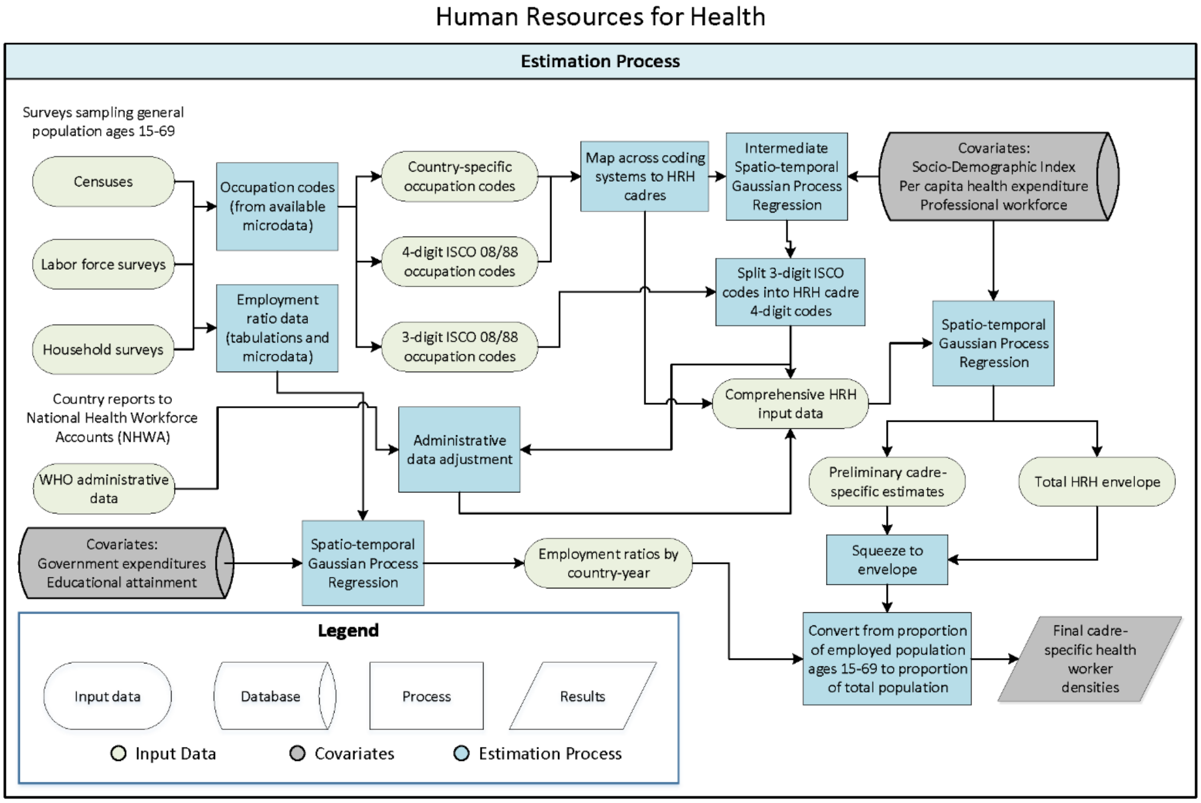

1.1 Input data and indicators

Our estimates of health worker densities leveraged nationally representative cross-sectional surveys and censuses conducted between 1990 and 2019 to identify members of the general working-age population who self-reported current active employment in a health-related occupation. All such published sources were identified and obtained either through the Global Health Data Exchange (GHDx) using occupation-related keyword searches (eg, “Occupations,” “Occupational risk factors”) or through the International Labour Organization’s (ILO) tabulations and database of employment-related surveys and censuses. Working age was defined as ages 15 to 69, since this was the standard age range for most labour force surveys. The two types of indicators extracted from these sources were employment ratios and occupation distributions.

The employment ratio indicator reflects the proportion of the working-age population that self-reports currently being employed. The corresponding dataset consisted of all downloadable data tabulations of employment-to-population ratios by age and sex available from the ILO, as well as employment levels that we extracted directly from individual-level survey microdata obtained through the GHDx.

Typical survey questions for identifying active employment asked individuals if they had worked for at least one hour in the previous seven days in any of the following capacities: for a wage, as an apprentice, in self-employment, or for a family business. Those reporting only a temporary absence from such a position in the preceding seven days were also considered to be employed.

Among employed individuals, occupation distributions were determined from a respondent's self-reported description of their main job in the previous seven days, or their typical main job if they were temporarily absent from work. The corresponding extracted indicator was the proportion of the employed population working in different occupational categories. Occupation descriptions were coded according to the source's occupational coding system, often a version of the International Standard Classification of Occupations (ISCO) or a closely related system. The distribution of our data across these coding systems is summarized in table 2. Such systems apply standard criteria for classifying distinct occupations. Details on the classification criteria for each occupation within ISCO can be found on the ILO website. Coding systems also range dramatically in granularity, typically reflected by the length of the codes themselves, and can thus classify respondents into broadly defined categories of work or provide very specific descriptions of their occupations.

Table 2. Input sources by coding system and level of granularity

Coding system	Number of country-years
Country-specific coding system	78
ISCO 08 4-digit	3038
ISCO 08 3-digit	181
ISCO 88 4-digit	322
ISCO 88 3-digit	588
Total	4207

To meet this study's inclusion criteria for occupation data, a survey needed to code respondent occupations to a level of granularity that could identify health workers directly or identify a small aggregate group from which health worker cadres could be accurately split out. This level of granularity corresponds to ISCO three-digit and four-digit codes, as well as those alternate coding systems that could be mapped to ISCO at such a level of detail based on available documentation. This requirement restricted many of the eligible sources, since most standard international survey series and censuses do not code occupations to the level of detail required to identify health workers, let alone specific cadres of health workers. Due to the large number of distinct occupations at this level of granularity, few identified sources released tabulated data that met our inclusion criteria. Consequently, included population-based sources were those for which individual-level microdata could be accessed to allow direct extraction of occupational codes (employment ratio data were directly extracted from these sources as well). In addition to microdata files already accessible through the GHDx, we searched the ILO database of sources containing occupation data coded to at least two digits of ISCO granularity to identify publicly available microdata that also met this study's inclusion criteria. All such sources were added to the GHDx and subsequently extracted. In total, 1,473 microdata sources with employment and occupation data were identified and extracted through the GHDx, 69 of which were censuses and 1,404 of which were surveys. Surveys included labour force surveys and household surveys with sufficient labour-related questions. Given the similar nature of both survey types, distinctions between the two are not made in this paper. Both are included in any reference to "labour force participation surveys" in the main text and appendix.

Table 3. Input and output data source counts by adjustment status and data type

Input data	
Administrative data	2,950
Adjusted administrative data	2,307
Location-specific adjusted administrative data	976
Super-region/Region-specific adjusted administrative data	1,331
Labour force surveys	1,404
Censuses	69
**Output data**	
Modelled Estimates	5,916

*Administrative data sourced from the WHO GHO database

In addition to the survey and census data, we included data from the World Health Organization (WHO) Global Health Observatory database[17]. The WHO Global Health Observatory database reports health worker data with sufficient granularity (four digit ISCO-08) to meet our inclusion criteria. By incorporating this data source, we added 2,950 country-years of data for the four main cadres in our study: physicians, nurses and midwives, pharmacists and pharmacist technicians, and dentists and dental assistants. Figures 2a – 2d summarize the total number of country-years comprised in all data sources employed in this study.

Figure 2a. Country-years of occupation data for physicians, by country and territory, 1990-2019

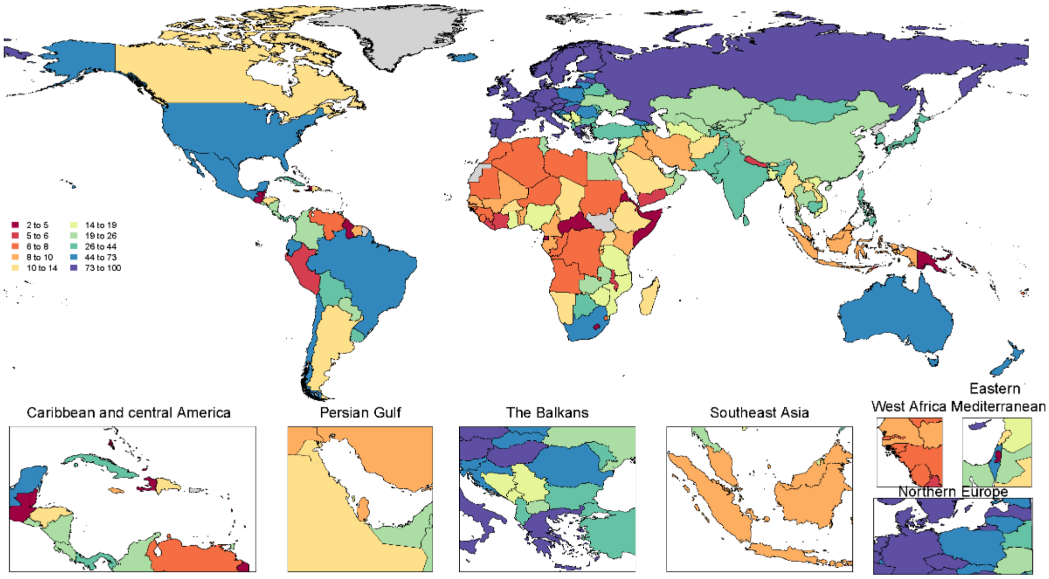

Figure 2b. Country-years of occupation data for nurses and midwives, by country and territory, 1990-2019

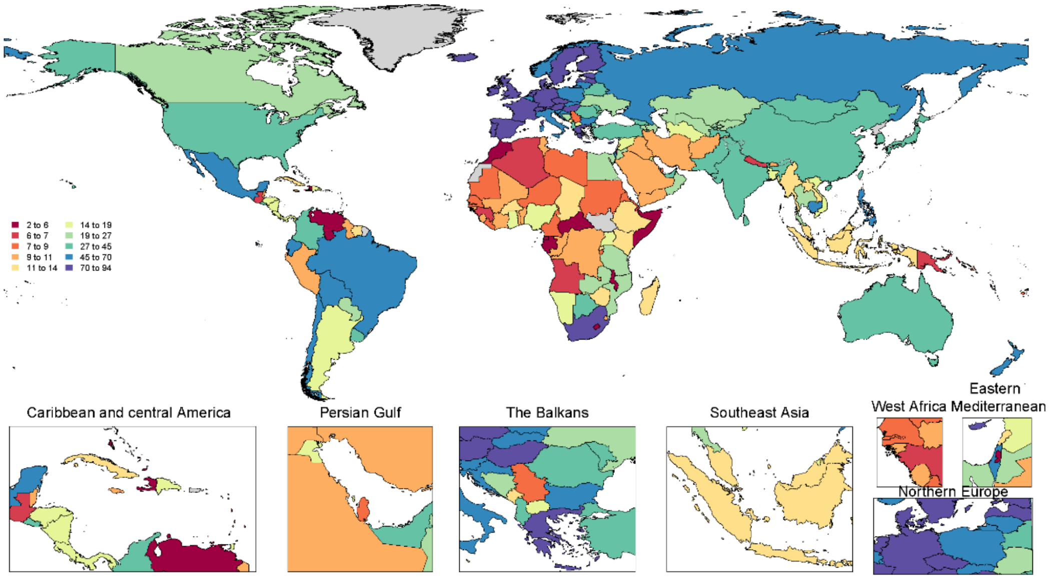

Figure 2c. Country-years of occupation data for dentists, by country and territory, 1990-2019

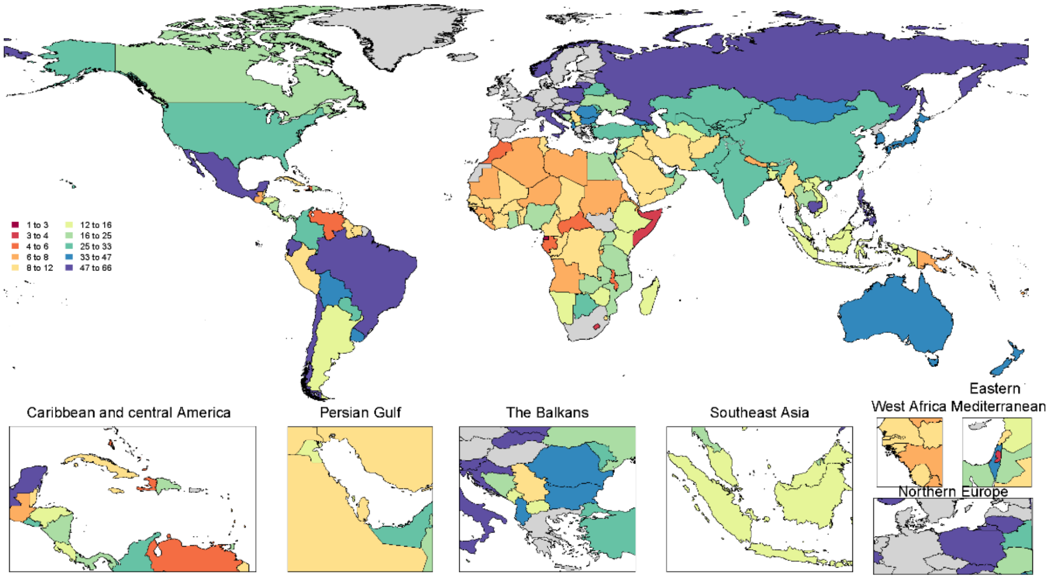

Figure 2d. Country-years of occupation data for pharmacists, by country and territory, 1990-2019

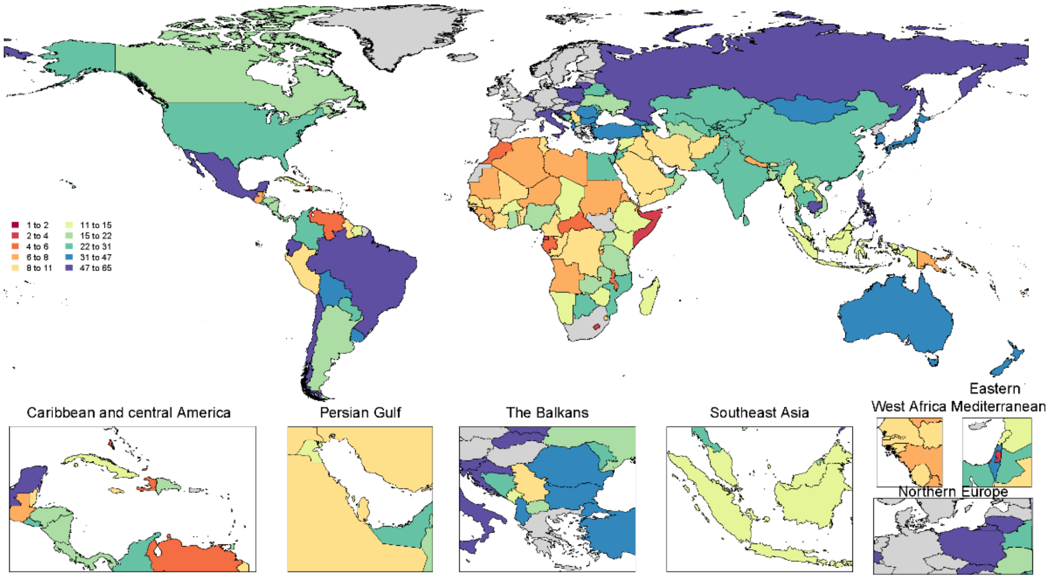

This study considers surveys and censuses to be the gold standard for measuring human resources for health because they are both population-based sources. Both sources rely on population-based sampling and tend to employ nearly identical occupational questionnaires and coding structures. While surveys and censuses do differ in their sample sizes and corresponding sampling errors, these are random errors that do not constitute systematic biases. Therefore, we refer to surveys and censuses as comparable source types, even though they may differ in their level of precision. Labor force surveys and censuses avoid many of the potential biases affecting other data sources, such as concerns related to the quality, coverage, and maintenance of administrative records, payrolls, and registries, which can result in both over-reporting and under-reporting of HRH levels as well as double-counting. For instance, WHO reports that the data in the Global Health Observatory database are reported by countries themselves through the National Health Workforce Accounts reporting portal, but limited information is publicly available about the source of data, any assumptions or modelling used to produce the data, whether data only captured the public sector, whether the data could have double-counting, and any other features. We provide a detailed discussion of the adjustment process we use for the WHO data in section 1.4 below.

1.2 Defining health worker cadres

We referred to the WHO Handbook on Monitoring and Evaluation of Human Resources for Health to create a list of relevant health worker cadres identified by four-digit ISCO 88 codes, the highest level of granularity in the coding system.[5] While some of these cadres are themselves an aggregation of multiple types of health workers that we would have wished to identify individually, we were constrained by the preponderance of data using coding systems that did not provide such levels of detail in their coding structures. The included cadres and their corresponding occupation codes are listed in table 3.

Table 4. Cadres and corresponding occupation codes

Health worker cadre	4-digit ISCO 88 code
Physicians*	2221
Nurses and midwives *	2230, 3231, 3232
Pharmacists*	2224
Pharmaceutical assistants*	3228
Dentists*	2222
Dental assistants*	3225
Physiotherapists and prosthetic technicians	3226
Medical imaging and therapeutic equipment technicians	3133

Medical laboratory technicians	3211
Clinical officers, medical assistants, and community health workers	3221
Health-care aides and ambulance workers	5132, 5139
Environmental health workers	3222
Optometrists and opticians	3224
Dieticians and nutritionists	3223
Audiologists, speech therapists, and counsellors	3229
Psychologists	2445
Home-based personal care workers	5133
Traditional and complementary practitioners	3241

* Included in the definition of SDG indicator 3.c.1 and in this study's minimum threshold analyses. Similar cadres were grouped together in this study, such that the 20 occupations listed above were consolidated into 16 cadres for the purposes of the analysis. See the threshold analysis section for additional details on cadre groupings.

The ISCO has standardised occupation codes, with documentation describing each in detail, and has organised them hierarchically. Codes for the most general category of occupations are one digit in length. Occupational sub-categories are represented by two-digit codes, and further sub-specialties are represented with three-digit and four-digit codes. ISCO also consists of multiple versions, which were developed over time to update the system to reflect modern labour markets. The two versions included in this analysis are ISCO 88 and ISCO 08, released in 1988 and 2008, respectively, which thus cover the majority of the study's time period of interest. Although the two versions of ISCO are generally similar in their distinctions between occupations, they differ in their hierarchical structuring of the coding system, with ISCO 08 consolidating health workers at higher levels of the hierarchy and facilitating the identification of more detailed occupations at its most granular level.[6] However, ISCO 88 was substantially more common among identified sources, as shown in figure 3.

Figure 3. Number of sources by coding system, 1980-2019

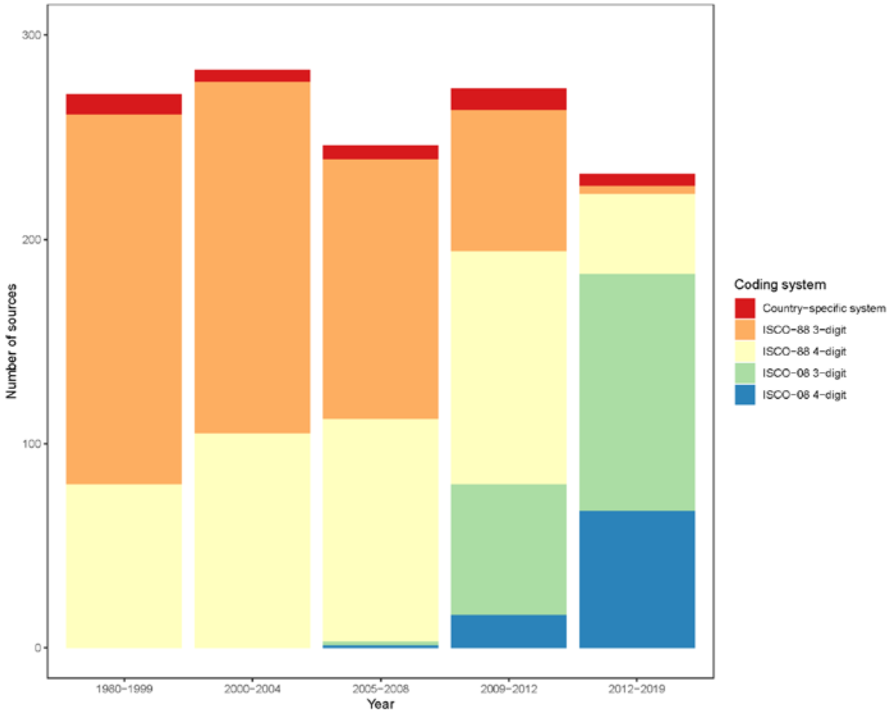

Mapping between versions inherently results in some loss of precision, and utilising the greater granularity of ISCO 08 for a few cadres would require using available ISCO 08 data to split out the less detailed ISCO 88 categories into smaller occupations. Given the paucity of ISCO 08 sources and the limited temporal overlap in the use of both versions, mapping all data to the ISCO 08 system and splitting less granular codes as necessary was not tenable for

this analysis. Consequently, we used ISCO 88 as the gold standard according to which the HRH cadres in this study are defined. At a point when a much greater proportion of surveys rely on ISCO 08, future iterations of this analysis might be able to use ISCO 08 and thus generate more granular cadre-specific estimates. Figures 2 and 3 below present examples of these two coding frameworks.

Figure 4a. Example subsection of the ISCO 88 hierarchy

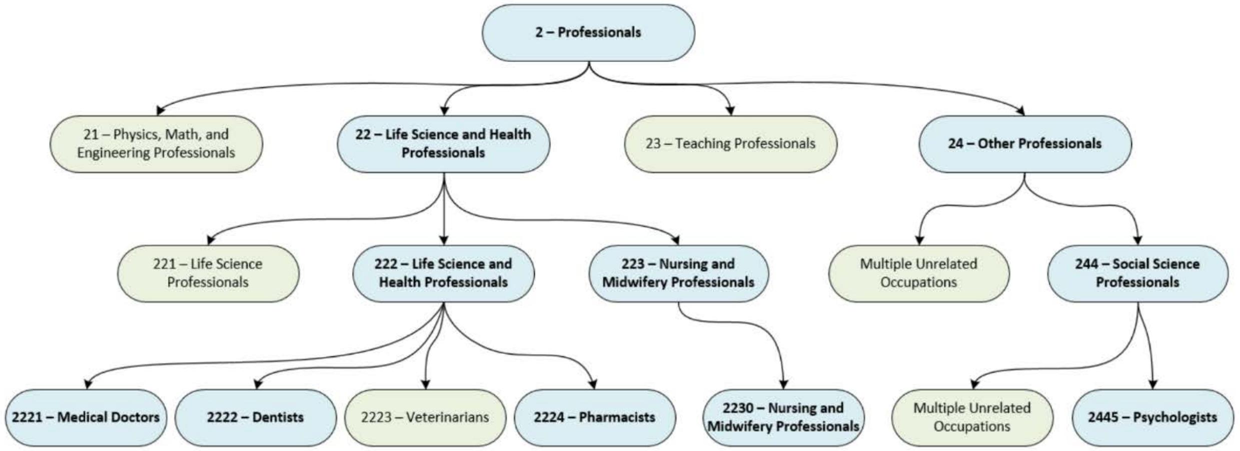

Note: only relevant occupations shown.

Figure 4b. Example subsection of the ISCO 08 hierarchy

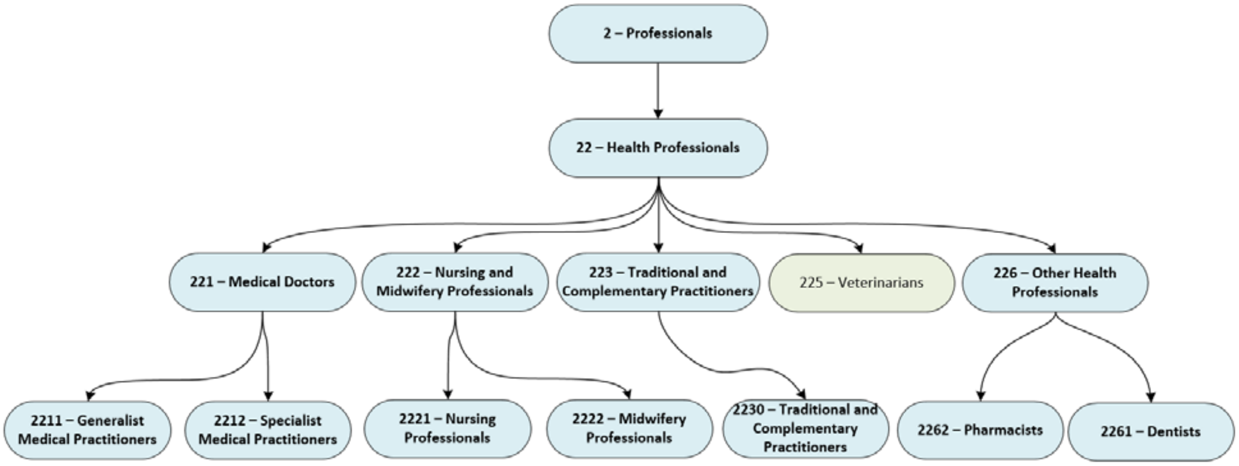

Note: only relevant occupations shown.

In figures 4a and 4b, occupational categories and their corresponding codes are shown in a tree structure, reflecting the respective hierarchies of the ISCO system. At the top, one-digit codes identify very general categories of occupation. At the bottom, longer codes use additional digits to differentiate occupations at greater levels of detail. Marked in blue are codes for the HRH cadres included in this analysis, or less-granular codes which contain an HRH cadre within them. More details on the criteria used to differentiate occupational classifications are available on the ILO's website pertaining to ISCO.[6]

For some sources that claimed to use an ISCO version, microdata sometimes exhibited a few codes that did not adhere to standard ISCO frameworks. In all such cases, the few non-ISCO codes were slight deviations from the

standard framework, with one or two of the last digits altered either to identify particular occupations not normally highlighted in ISCO, or to express ambiguity when an occupation description was not quite detailed enough for ISCO classification at the desired digit level of detail. Descriptions of such deviations were usually missing from source documentation. In the absence of explanatory documentation, we truncated individual ambiguous codes to the longest length that was valid in the corresponding ISCO system. For example, we truncated invalid four-digit codes to valid two-digit or three-digit codes. When the truncated code was known to be unrelated to the HRH cadre in question (eg, the code pertained to a non-specific type of farmer), which was usually the case, the truncation did not have an impact on the extracted data. In some cases, however, it was unclear whether the truncated code was related to an HRH cadre of interest (eg, a truncated code for social science professionals might have pertained to psychologists or to another non-HRH social scientist). If a source contained a large number of invalid codes with potential relevance to HRH, then the source's entire set of occupation codes was truncated by one digit, and the source was dropped entirely if truncation resulted in occupation codes of less than three digits. If a survey contained few invalid codes with potential relevance to HRH, then all codes were retained and any HRH-ambiguous codes were excluded from both numerator and denominator of the extracted data for only those HRH cadres for which they were ambiguous. For instance, a truncated code potentially pertaining to psychologists was excluded from both numerator and denominator for the psychologist data due to ambiguity, but was still included in the denominator for every other HRH cadre to avoid biasing their respective estimates. Consequently, some sources exhibited minor deviations in extracted sample sizes across the various cadres of interest.

1.3 Mapping and splitting

All usable data were mapped to ISCO 88 four-digit codes for this analysis, or were split to such codes if data corresponded to the less detailed three-digit level of granularity. When surveys used country-specific coding systems that were based on ISCO, mapping to ISCO codes could be accomplished easily. When surveys used coding not clearly based on ISCO, only occupations where associated descriptions sufficiently matched those of ISCO were mapped.

In order to map ISCO 08 codes to ISCO 88 codes, we referenced the ILO's ISCO concordance documentation. Frequently, precise matches between four-digit codes existed between versions, allowing exact mapping from one version to the other.[6] At other times, a four-digit ISCO 88 code corresponded to an aggregation of multiple ISCO 08 codes, which also allowed exact mapping to ISCO 88. When occupation categories between versions did not exactly match or aggregate to one another, we created an approximate mapping, whereby the most common ISCO 88 code corresponding to an ISCO 08 code in the concordance documentation was considered to be the sole match and was mapped accordingly. Table 4 provides examples of health worker cadre mapping across ISCO versions.

Table 5. Examples of health worker cadre mapping across ISCO versions

Occupation titles	ISCO 08 code	ISCO 88 code	ISCO 88-defined health worker cadre	Type of mapping
Dentists	2261	2222	Dentists	Exact match
Clinical officers	2240	3221	Clinical officers, medical assistants, and community health workers	Exact aggregation
Medical assistants	3256	3221	Clinical officers, medical assistants, and community health workers	Exact aggregation
Community health workers	3253	3221	Clinical officers, medical assistants, and community health workers	Exact aggregation
Nursing Professionals	2221	2230	Nurses and midwives	Exact aggregation
Midwifery Professionals	2222	2230	Nurses and midwives	Exact aggregation
Nursing Associate Professionals	3221	2230	Nurses and midwives	Exact aggregation
Midwifery Associate Professionals	3222	2230	Nurses and midwives	Exact aggregation

Environmental health workers	2263	3222	Environmental health workers	Approximate match
Food inspectors	3257	3222	Not HRH (ISCO 08) / Environmental health workers (ISCO 88)	Approximate match
Occupational hygienists	2263	3152	Environmental health workers (ISCO 08) / Not HRH (ISCO 88)	Approximate match
Quality controllers, Electrical product inspector, etc.	3257	3152	Not HRH	Approximate match

Our objective in mapping across ISCO versions was to create consistency and retain as much information as possible, but some inconsistencies and information loss were unavoidable. The table above draws on ISCO concordance documentation to illustrate how we mapped four-digit health worker codes from ISCO 08 to ISCO 88.[6] For example, the table shows that for dentists there is an exact match between ISCO 08 and ISCO 88, which made it possible to map dentists across versions with no information loss. For other occupation titles, matching to comparable occupation categories was possible, but it resulted in a loss of granularity. For example, while ISCO 08 placed clinical officers, medical assistants, and community health workers into separate occupational categories, each with a different code, ISCO 88 grouped these into one category with a single code. Similar aggregations of seemingly divergent occupations are seen in other ISCO-88 HRH codes, such as audiologists, speech therapists, and counsellors (for which counsellors encompass a broad range of specialties, including family planning and HIV).

In other cases, the grouping of occupations between versions could not be made entirely consistent without including large numbers of non-health-related occupations, as was the case with environmental health workers. ISCO 88 groups environmental health workers with food inspectors, whereas ISCO 08 groups them with occupational hygienists. Finding an exact match between versions would require including codes that also pertain to quality controllers, electrical product inspectors, and a wide range of other non-health occupations. Instead of including a large number of unrelated professions, we settled on an approximate mapping between versions, such that the environmental health workers cadre identified from ISCO 88 sources would also include food inspectors, whereas the cadre identified from ISCO 08 sources would include occupational hygienists. This resulted in some inconsistency but was preferable to dropping the cadre entirely or diluting it with many unrelated occupations.

Three-digit ISCO occupation codes were common, particularly in the censuses included in this study. While three-digit ISCO codes generally did not allow us to identify specific HRH cadres, information from three-digit occupation codes was useful in creating envelopes from which individual health worker cadres could be split. To do this, we first used data extracted from surveys with four-digit codes to inform preliminary models of each health worker cadre. We used these preliminary models to split three-digit code data into cadre-specific estimates for each GBD location and year. This required modelling not only each HRH cadre, but also the residual categories made up of four-digit codes associated with each three-digit code of interest that did not correspond to any health worker cadre. The advantage of using four-digit data to split three-digit codes rather than enacting one global split is that the proportional makeup of health worker cadre estimates exhibited variation across space and time. Yet, because splits were informed exclusively by four-digit inputs, data prepared from three-digit surveys were dependent upon the quality and coverage of four-digit data.

For country-specific coding systems not derived from ISCO, there was considerable variation in the level of detail available for different health worker cadres, and the digit length of an occupation code was generally not a good predictor of granularity. A two-digit country-specific occupation coding system, for instance, might distinguish between multiple categories of physicians, while using only one code for all nurses and midwives. When it was possible to match or aggregate some country-specific codes to the four-digit ISCO 88 framework, we mapped those codes accordingly. Where codes could be split into multiple estimated cadres without residual groups, we used the method described above for three-digit ISCO codes. Other types of country-specific codes were not usable, as we did not have enough information to map them to ISCO 88. Consequently, sources using country-specific coding systems typically only provided data for a subset of the HRH cadres included in this analysis.

We also generated a dataset for estimating the aggregate of all health workers combined. Data for this group comprised the sum of density data for all cadres – after mapping and splitting – that were obtained from sources

whose coding system allowed identification of every cadre in this study (including, for example, all sources that used ISCO 88 or 08 coding).

1.4 Input data adjustment strategy

We consider the survey and census data to be the “gold standard” for measuring human resources for health because they are population-based and nationally representative. In an effort to use all available sources of data, we also included the WHO Global Health Observatory database in our analysis. Besides their origin with countries, there is little information about how these data came about, including any potential biases. Thus, we needed to evaluate the WHO data and determine whether the data were systematically biased from the population-based data. To perform this analysis, we matched the WHO data with survey or census data by country and year, resulting in 2636 matched pairs. Where we had both a survey and a census data point, we took the average to compare to the WHO data point. We depict scatter plots of the matched pairs of WHO data and population-based data for the cadres with more than 300 data points from the WHO, namely physicians, nurses and midwives, dentists and pharmacists (figure 5a – figure 5d).

Figure 5a. Matched pairs of IHME population-based points and WHO data points for physicians, 1990-2019

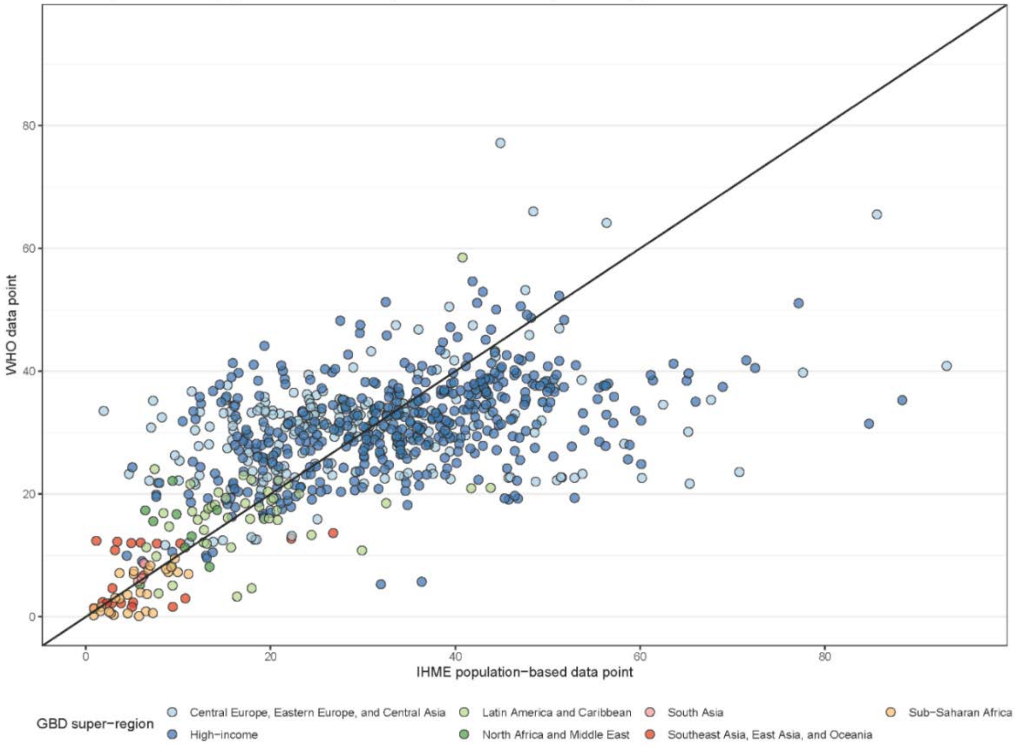

Figure 5b. Matched pairs of IHME population-based points and WHO data points for nurses and midwives

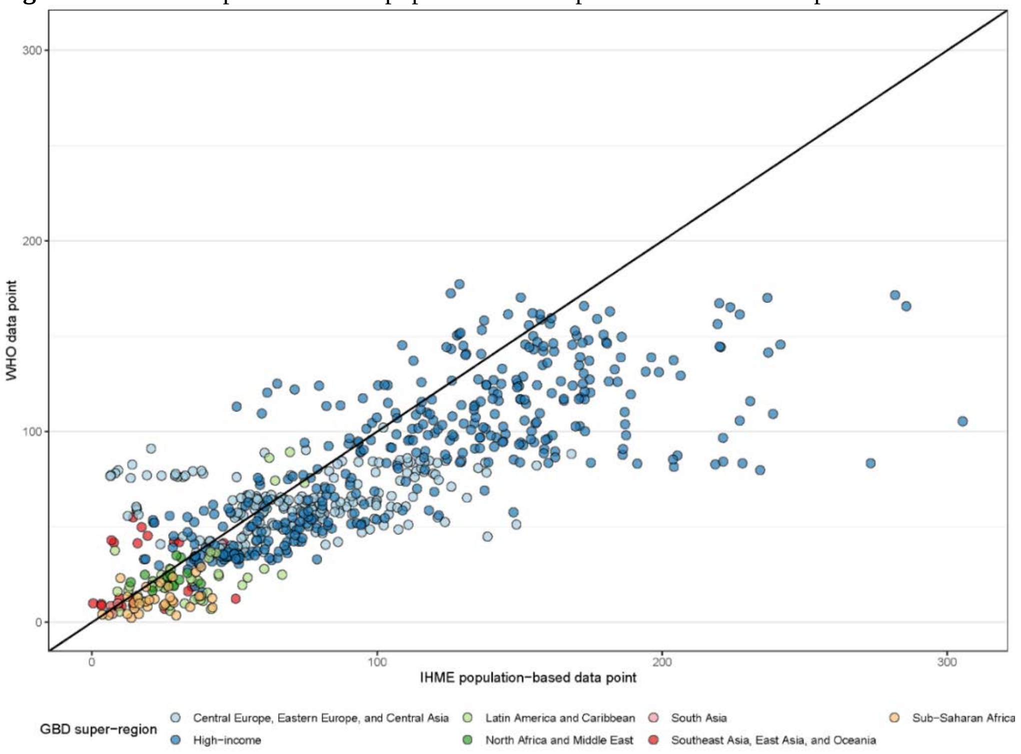

Figure 5c. Matched pairs of IHME population-based points and WHO data points for dentists

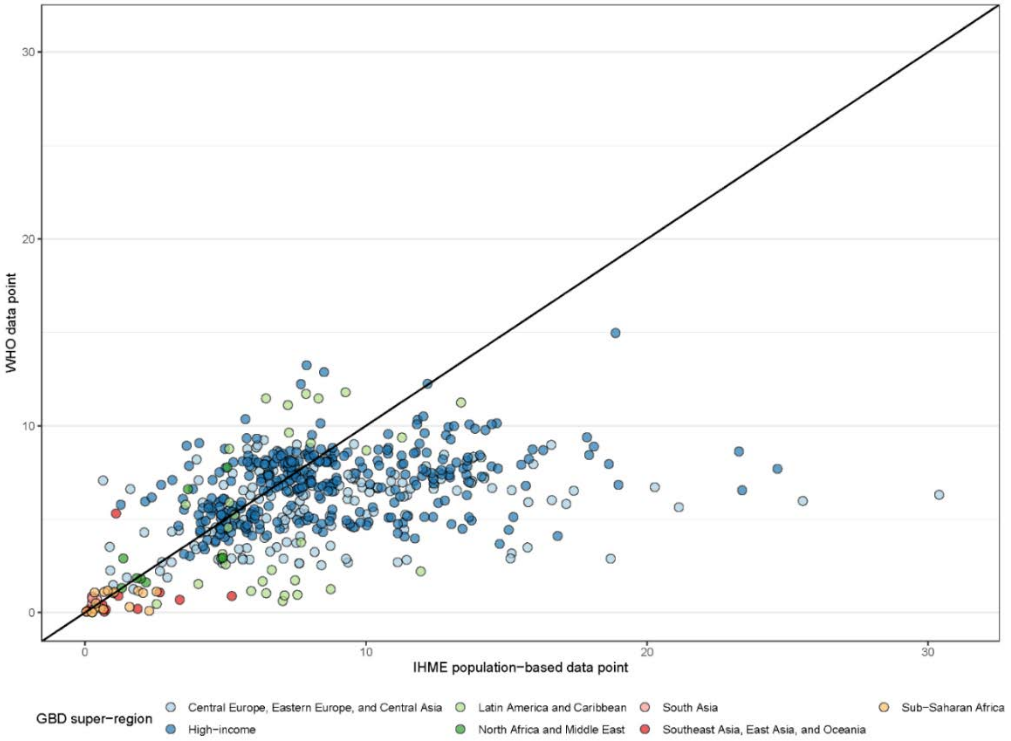

Figure 5d. Matched pairs of IHME population-based points and WHO data points for pharmacists, 1990 - 2019

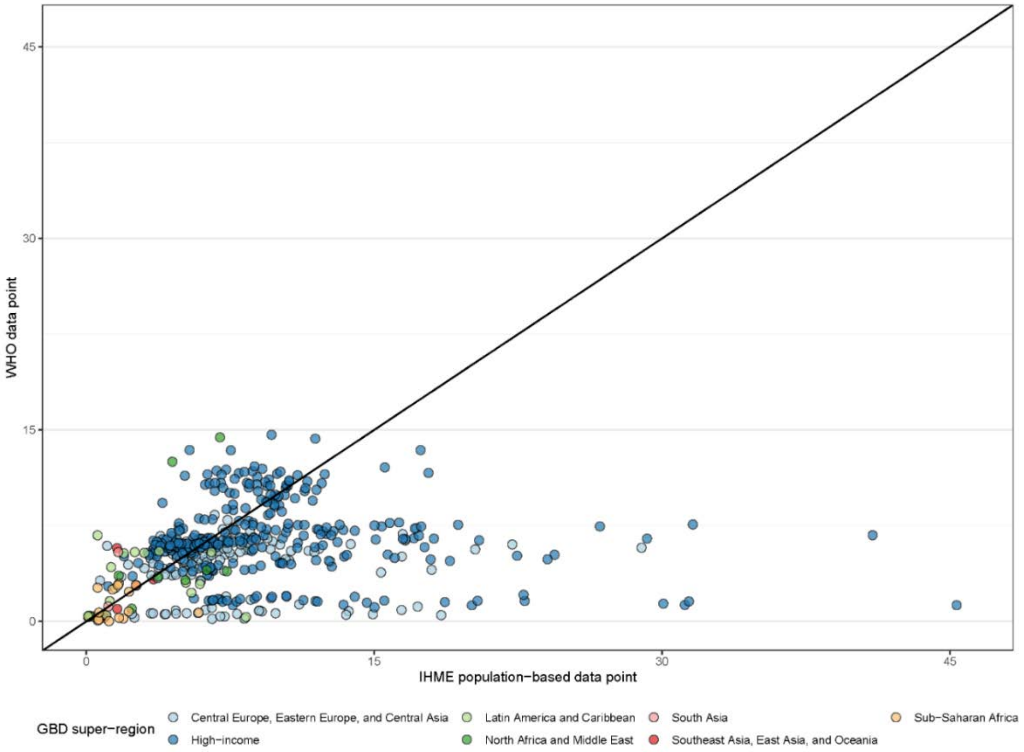

In figures 6a-6d, we provide maps of the average percent difference between the WHO points and the population-based data by country and territory for each of these cadres.

Figure 6a. Average percent difference between IHME population-based data and WHO data for physicians

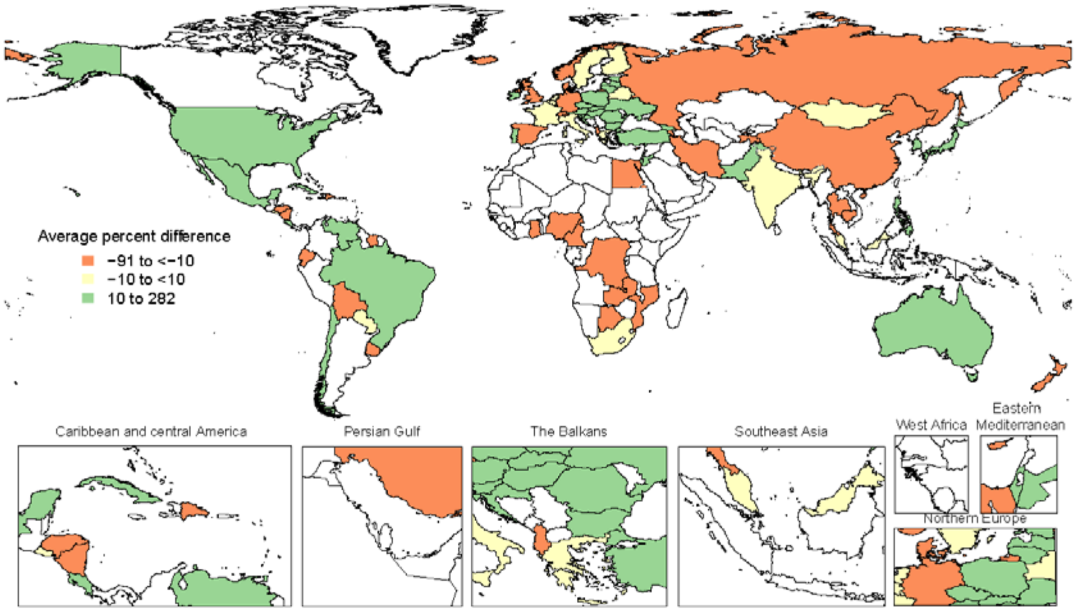

Figure 6b. Average percent difference between IHME population-based data and WHO data for nurses and midwives

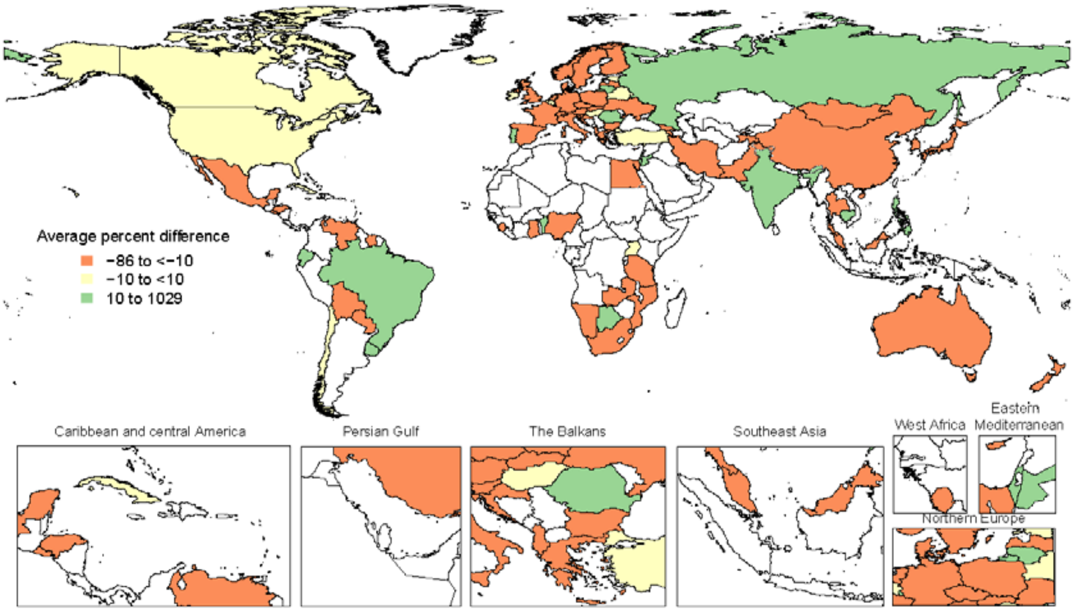

Figure 6c. Average percent difference between IHME population-based data and WHO data for dentists

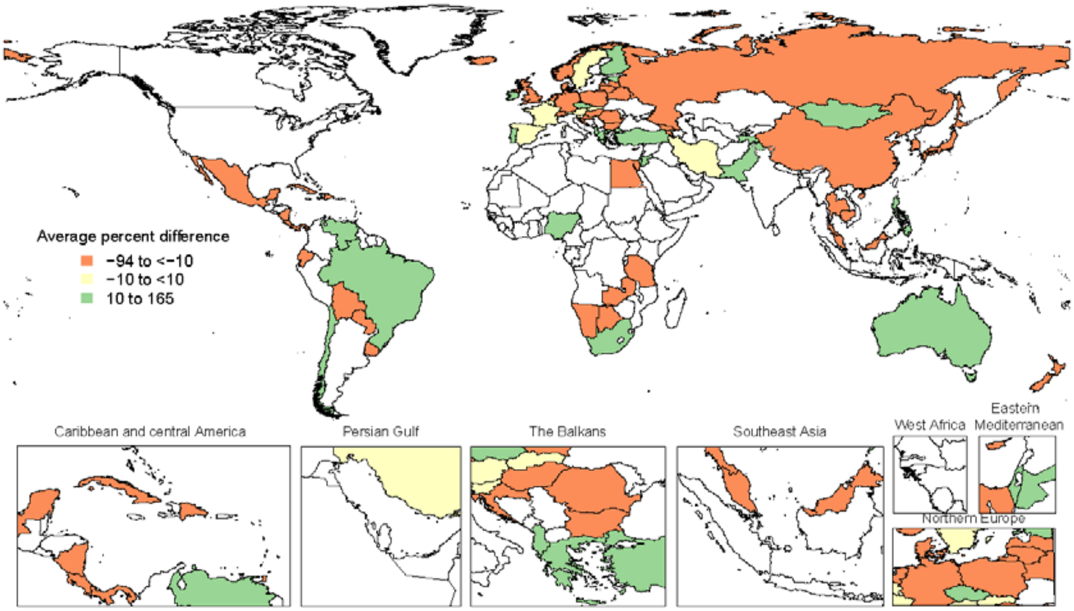

Figure 6d. Average percent difference between IHME population-based data and WHO data for pharmacists

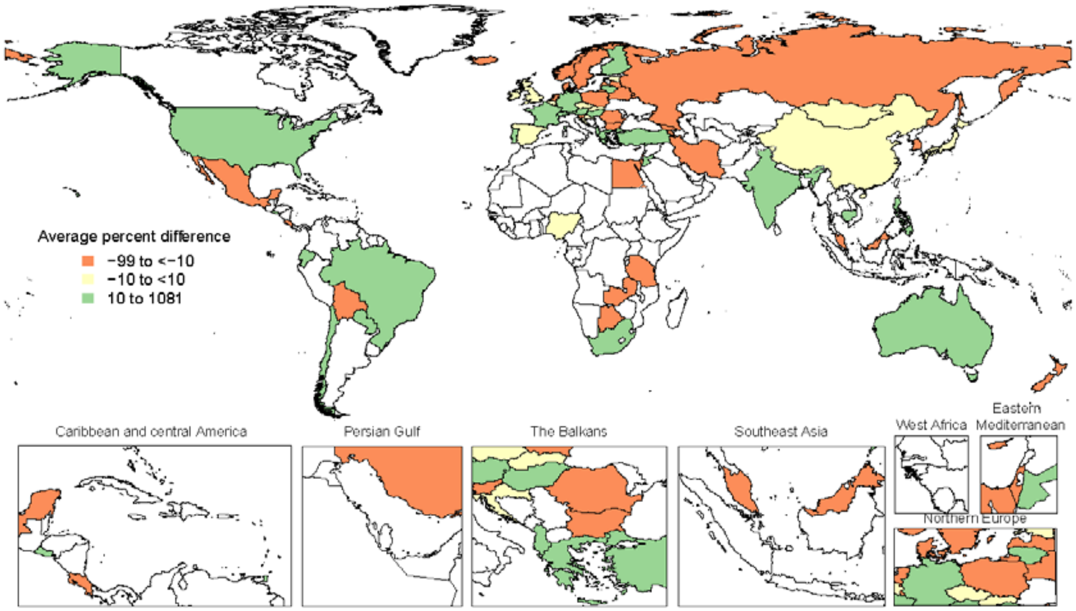

Table 6. Average percent difference between IHME population-based data and WHO data for all cadres

Super-region/Region	Physicians	Nursing and midwifery personnel	Dentists	Pharmacists
Central Europe, Eastern Europe, and Central Asia	9.83	-0.91	-14.16	-19.80
High-income	0.56	-4.64	-3.89	-3.23
Latin America and Caribbean	4.05	-5.04	-7.44	85.90
North Africa and Middle East	11.57	-12.13	4.55	15.38
South Asia	1.46	-0.39	31.71	5.71
Southeast Asia, East Asia, and Oceania	8.14	24.08	-22.78	2.60
Sub-Saharan Africa	-10.88	-18.82	-17.26	-27.73
Location				
Central Europe, Eastern Europe, and Central Asia	11.04	4.44	-11.58	-24.13
High-income	-0.11	-10.40	-4.67	-8.88
Latin America and Caribbean	0.70	-12.93	-22.23	86.59
North Africa and Middle East	21.68	-16.41	15.50	37.69
South Asia	8.18	-2.90	128.09	104.43
Southeast Asia, East Asia, and Oceania	0.85	15.93	-38.53	41.82
Sub-Saharan Africa	-12.96	-26.02	-27.11	-16.16

Figures 6a-6d highlight that the WHO data were not consistently biased in one direction or another, although regional patterns are apparent. For example, WHO data tends to be lower than population-based data in sub-Saharan Africa across all cadres, suggesting the WHO data for the region potentially only captures the public sector. Another regional trend depicted figure 5a is that the WHO physician data appears to be systematically higher than population-based sources in eastern Europe. Based on these types of regional patterns, we performed bias adjustments based on GBD region and GBD super-region, where no matched pairs existed for a given location. We computed location-specific adjustments when matched pairs were available for a given location.

For each geographical unit, we tested whether there was sufficient evidence to implement an adjustment first. There were some locations where the WHO data were comparable to the population-based data we did not want to make

an adjustment. We initially tested whether a location needed adjustment using a lasso regression, with cadre per population regressed on indicators for region and super-region, or alternatively, location. Where the indicators were zero in the lasso regression, we did not conduct an adjustment. The penalty (lambda) in the lasso regressions was based on the 1st standard deviation from the mean minimum lambda selected from minimum root mean square error from cross-validation, iterated 1000 times to ensure fold selection did make our results unstable. Table 5 shows the lambdas selected and the RMSE from each lasso model. Figures 7a-7d show the countries in which a location adjustment was used, a super-region or region adjustment was used or no adjustment was applied.

Table 7. Lambdas and RMSE from the lasso covariate selection regressions, by cadre

	Location adjustment		Super-region/region adjustments	
Cadre	*Lambda*	*RSME*	*Lambda*	*RSME*
Physicians	0.010507	0.494645	0.011254	0.554383
Nurses and midwives	0.004176	0.409446	0.007937	0.484956
Dentists	0.009028	0.572764	0.014393	0.602905
Pharmacists	0.009017	0.589479	0.046735	0.989457

Figure 7a. Adjustment type for physicians

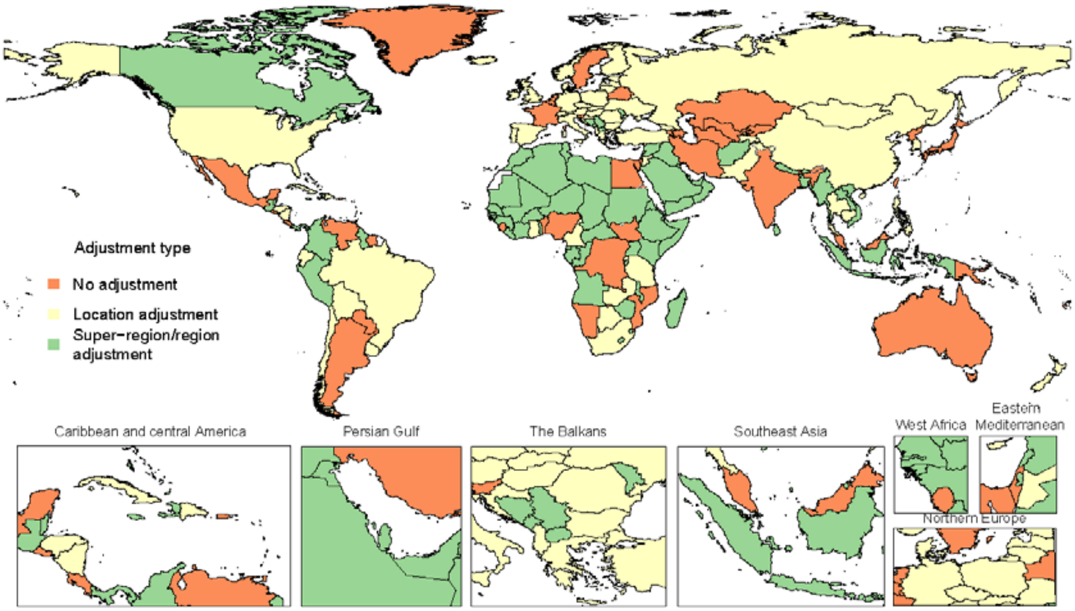

Figure 7b. Adjustment type for nurses and midwives

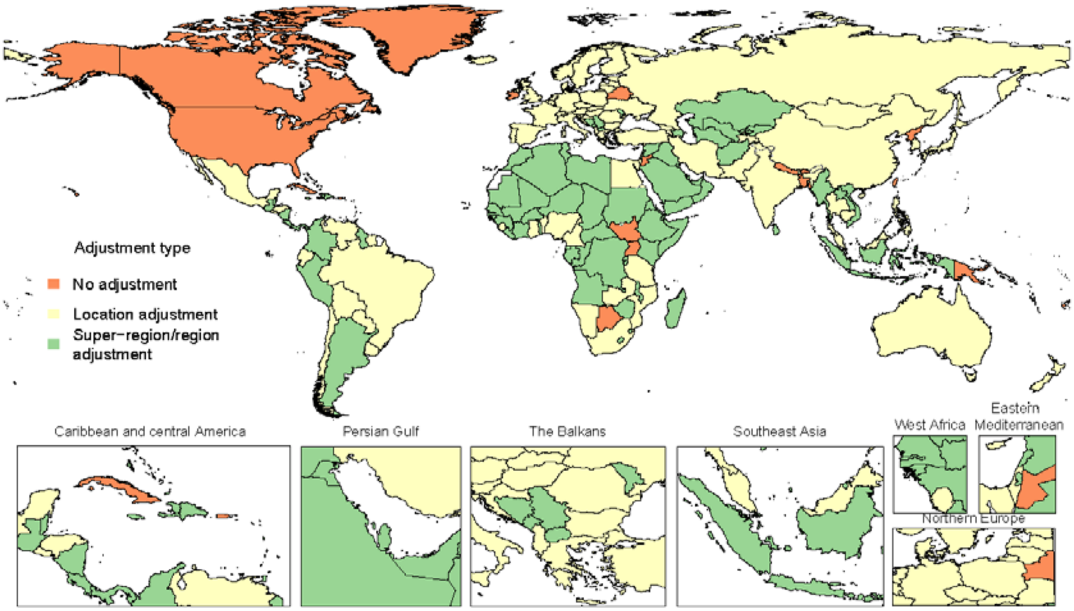

Figure 7c. Adjustment type for dentists

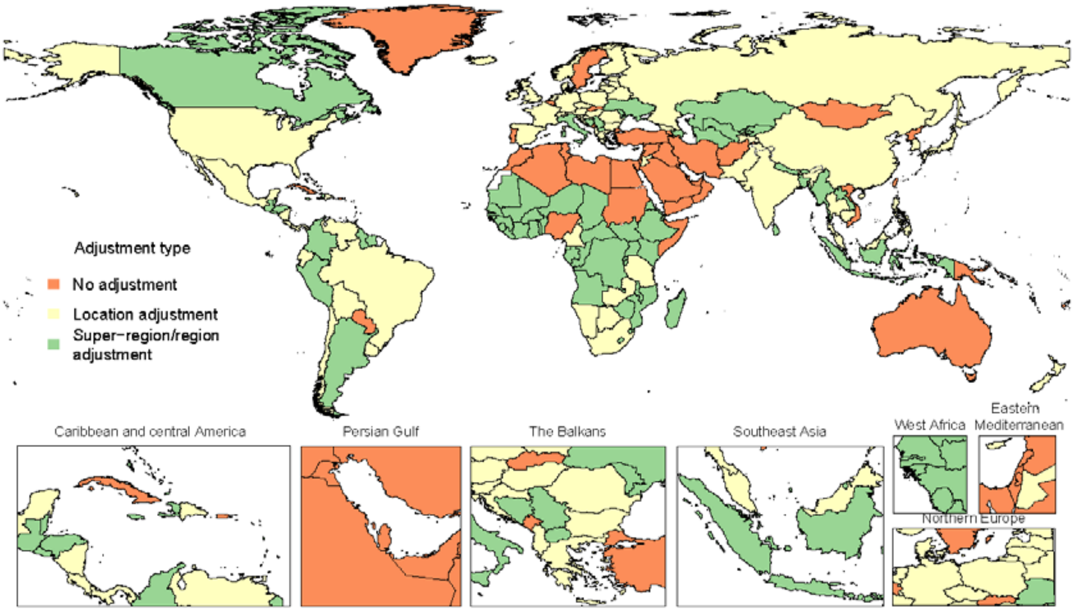

Figure 7d. Adjustment type for pharmacists

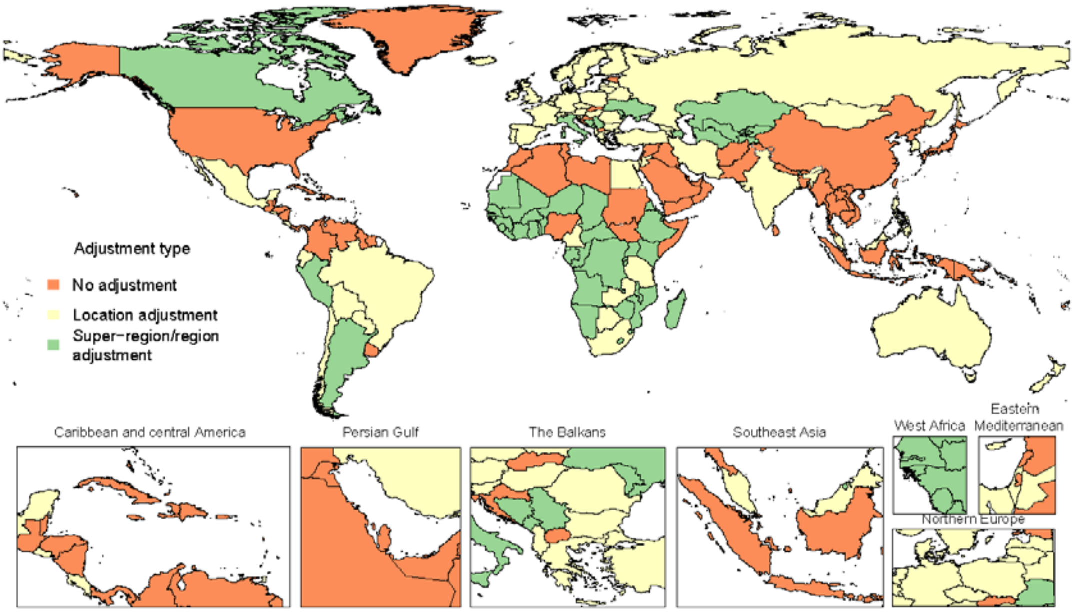

To implement the adjustment, we performed two crosswalks using the MR-BRT tool developed for GBD, estimating adjustments based on the ratio of population-based sources to WHO data.[18, 19] MR-BRT propagates model uncertainty and data uncertainty through to the adjusted estimates (of the WHO data), important for subsequent modeling steps. The WHO data do not have reported variance since they are sourced from reports from countries. We imputed variance of the WHO data based on the mean regional variance of the labor force surveys and censuses where population-based sources where available; we imputed the mean variance of these sources by super-region in locations without population-based sources. Because in some locations, regions and super-regions, data were sparse and we were concerned about over-fitting the adjustment model, we used a Gaussian prior in the crosswalk. The first crosswalk was for region-super-region adjustments, based on the indicators selected in the lasso regression with geographic indicators for region and super-region. The second crosswalk was for location-specific adjustments, with adjustments estimated only for locations selected in the lasso regression. We opted to perform a single super-region adjustment in sub-Saharan Africa instead of individual regional adjustments due to a limited number of matched pairs in the super-region (only 27 matched pairs for physicians for the super-region and just 1 matched pair for central sub-Saharan Africa in particular). Furthermore, we declined to implement super-region-region adjustments where the direction of the adjustment was inconsistent with population-based sources – if the adjustment would lower the WHO point but a population-based source was higher than the WHO point, we concluded we did not have sufficient evidence to implement the adjustment.

$$\ln\left(\frac{HRH\ cadre_i}{10{,}000\ population}\right) = \beta * I(location) + \varepsilon_i$$

$$\ln\left(\frac{HRH\ cadre_i}{10{,}000\ population}\right) = \beta * I(region) + \beta * I(super\ region) + \varepsilon_i$$

Where:

i denotes a given matched pair of population-based sources to WHO data
$I(location)$ denotes an indicator for each location selected in the lasso regression
$I(region)$ denotes an indicator for each region selected in the lasso regression
$I(super\ region)$ denotes an indicator for each super-region selected in the lasso regression
β is the adjustment factor for locations, regions or super-regions selected in the lasso regression, modeled with a Gaussian prior in MR-BRT

ε_i is the residual

Maps of the percentage adjustment relative of the WHO data are found in figures 8a-8d The MR-BRT crosswalk package computes uncertainty for each point, based on both data and model uncertainty.

Figure 8a. Average percent adjustment to the WHO data input data, physicians

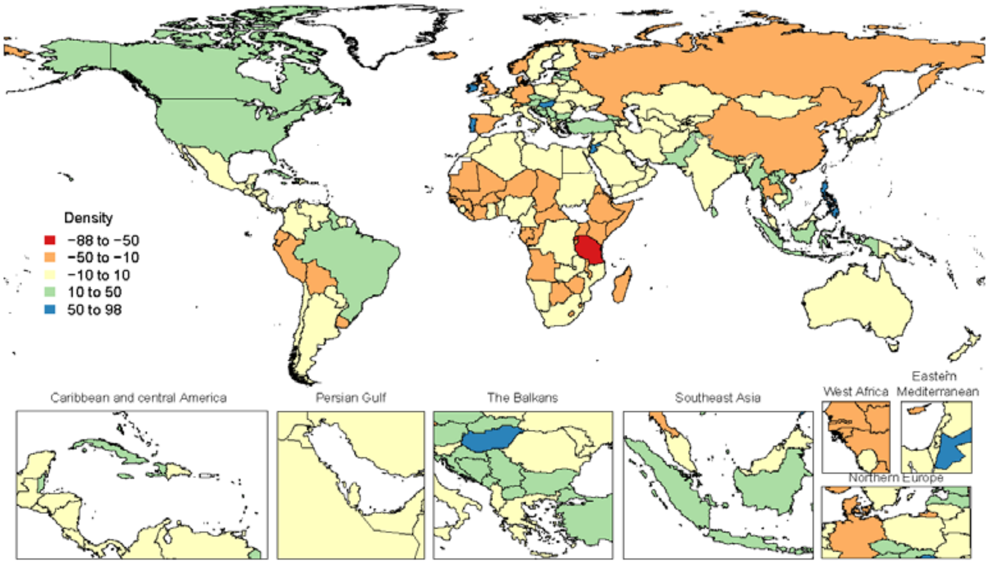

Figure 8b. Average percent adjustment to the WHO data input data, nurses and midwives

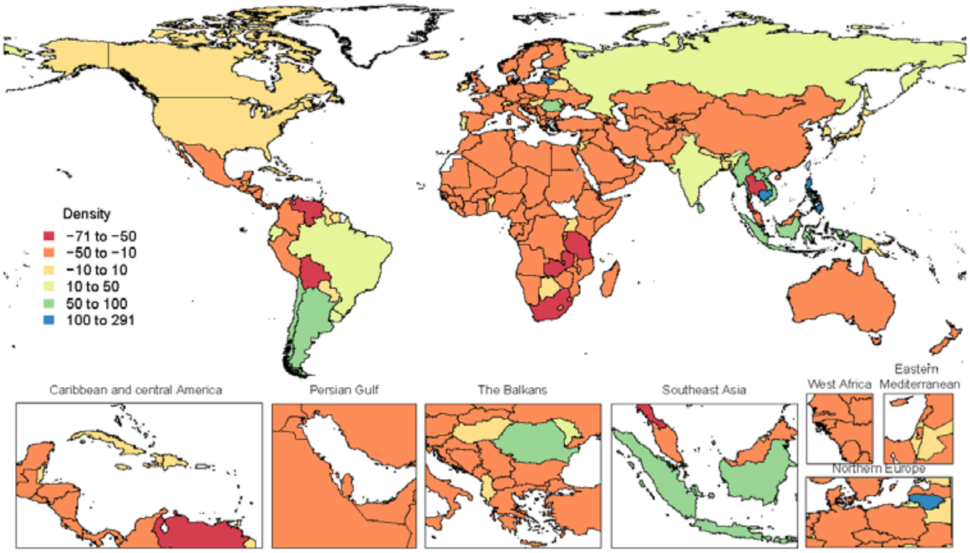

Figure 8c. Average percent adjustment to the WHO data input data, dentists

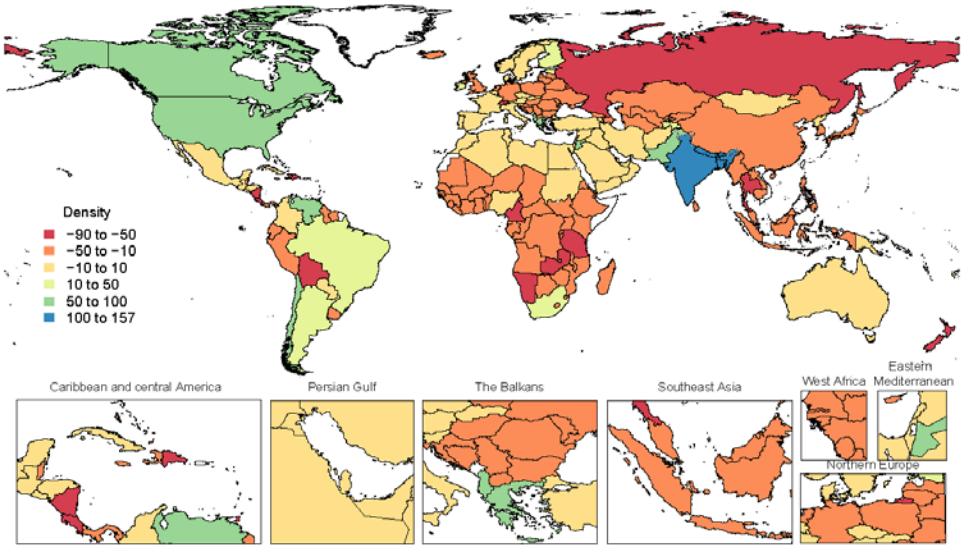

Figure 8d. Average percent adjustment to the WHO data input data, pharmacists

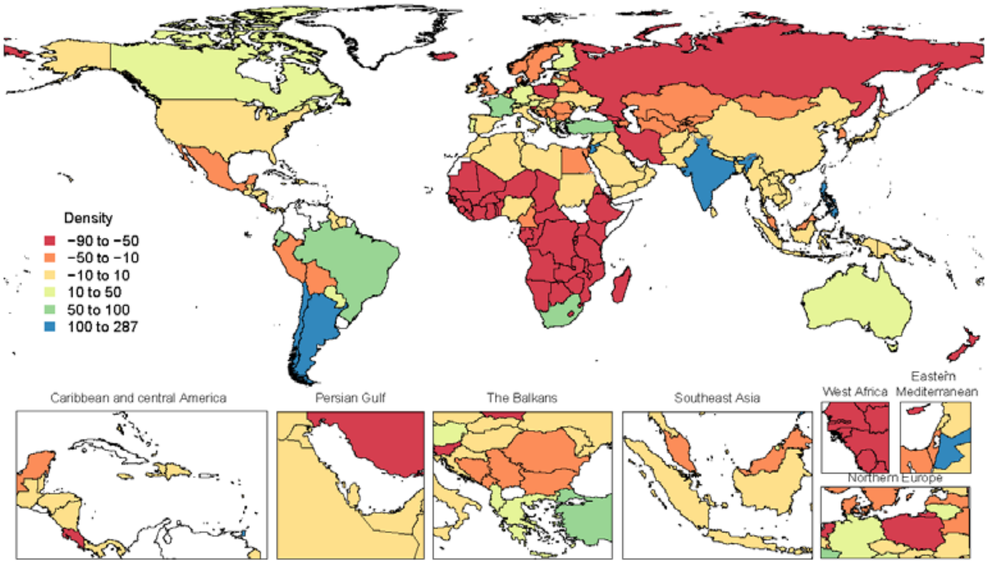

1.5 Modelling strategy

To estimate the prevalence of health workers around the world over time, this analysis used a three-stage spatiotemporal Gaussian process regression (ST-GPR). ST-GPR is a flexible modelling strategy that synthesises noisy data by incorporating covariates and borrowing strength across both geography and time to produce comprehensive time series estimates of an indicator with corresponding uncertainty. ST-GPR has been used widely in GBD research and has been described in detail in other recent publications.[7] As stated in the main text, the first stage of the model fits a linear regression to the data with random effects on specified covariates. The second stage smooths the residuals between the regression fit and the data across time and geography to generate a non-linear trend that better follows available data in each national location, as well as in the surrounding region and super-region. The third stage uses that trend as a mean function in a Gaussian process regression to account for input data variance and to generate uncertainty in the final estimates. In this study, ST-GPR was used to model 1000 draws of densities for each health worker cadre separately, all health worker cadres combined, and employment ratios, for every location from 1990 to 2019. Health workers were not modelled separately by age or sex.

Uncertainty was propagated through all analytical steps, such that final UIs reflect uncertainty from survey sampling as well as from the models themselves.

In addition to handling noisy data and providing estimates of uncertainty, the space-time smoothing component of ST-GPR made it an ideal tool for modelling HRH. Since available input data on this topic are heavily skewed toward high-income geographies like Europe, it was important to select a model that would not extrapolate findings from such data-rich areas to unduly influence estimates for locations lacking in data, which tend to have very different health worker densities and distributions. By incorporating data from surrounding regions and super-regions in the model fit, ST-GPR ensures that estimates for locations lacking in data better reflect patterns observed in inputs from nearby geographies, rather than patterns from the most data-rich locations. ST-GPR therefore makes the default assumption (unless the data indicates otherwise) that locations nearby geographically will follow similar patterns in HRH levels, though this assumption is partially mitigated by the use of additional covariates to inform location-specific differences in anticipated workforce densities. Geographical proximity in ST-GPR is determined from the GBD 2019 location hierarchy, which divides the world into seven super-regions, 21 regions, and 204 countries and territories. GBD regions and super-regions were generated to group countries that are similar in physical geography as well as in epidemiological profiles (for example, patterns in causes of death). Given the wide range of health topics estimated within the GBD framework, these regional distinctions do not always align perfectly with patterns in the disease or indicator being estimated. Nevertheless, these groupings were constructed to capture many potentially unmeasured contextual similarities (related to culture, climate, economics, health burdens, etc.) that produce similar trends across a wide range of health-related indicators. We assume that a country's regional grouping is no less informative to the modelling of health worker densities than it is to the modelling of other prevalent health issues (eg, heart disease, cancer, exposure to lead, etc.). While health workers are certainly needed everywhere, the local disease burden, training capacity, workforce demand (in terms of monetary and workplace incentives for workers), and general health system infrastructure are all incredibly relevant factors to a country's health worker densities that do follow distinct regional patterns.

In order to model health worker cadres – both separately and in the aggregate – in ST-GPR, we first used linear models with fixed effects on combinations of the following GBD 2019 estimated covariates: Socio-demographic Index (SDI), log-transformed total national per capita health expenditure, and estimates of the size of the professional workforce. Estimates of SDI and total national per capita health expenditure were generated by affiliated research groups using methodology described elsewhere.[8,9] The professional workforce size was calculated as the proportion of the employed population working in ISCO-defined professional occupations. Data for the professional workforce covariate came from the same types of censuses and surveys used in extracting health worker cadre data. However, many more censuses and surveys were available for this covariate due to the fact that professional occupations can be identified from even those sources that only code occupations to the ISCO one-digit level of detail. ST-GPR was used to model the professional workforce across all GBD locations and years. Additional details on the modelling process for professional occupations are available in the Global Burden of Diseases, Injuries, and Risk Factors Study 2017 comparative risk assessment appendix.[10] To model each health worker cadre, we used the same model settings for intermediate and final estimates. Intermediate estimates, which were exclusively used in splitting, were run using only four-digit mapped censuses and surveys, while final estimates were run on all available data after three-digit codes had been split into the underlying four-digit cadres. Covariates were primarily selected based on predictive power rather than any causal interpretation of their impact on HRH densities. However, each covariate does have some an expected relationship with HRH. First, we included the Socio-demographic Index (SDI), an index of lag-distributed income per capita, total fertility rates, and education, which represents social and economic development and has a strong relationship with health outcomes. Where SDI is higher, we would expect there to be a more educated workforce and better ability to pay for health care, both of which would increase HRH densities. We would also expect there to be better health outcomes, which could plausibly be connected to the size of the health workforce. Second, we selected total per capita health expenditure, a proxy for the expenditure on HRH. As total health expenditure grows, we would expect HRH densities to also increase – part of the higher spending is likely driven by more spending on health workers. Finally, to capture trends in the broader workforce that correspond with the health workforce specifically, we used the share of the employed population represented by the professional workforce. The professional workforce is defined as major group 2 in the ISCO-88 coding framework. In models that exclude one of the aforementioned covariates, the covariate was excluded because it did not prove to have strong predictive power for a given health worker cadre. For example, the total per capita health expenditure covariate was omitted from every cadre's modelable entity except the "all health workers" cadre because the covariate had low predictive power for those cadres. Because total health expenditure

captures variation in spending on all health workers rather than variation specific to a cadre, this covariate was not informative for the cadre-specific HRH models. Table 8 depicts the covariates that are employed in each of the models.

We generated employment ratio estimates in ST-GPR by age and sex, using a linear model with fixed effects on total government expenditure levels, average educational attainment in years, the proportion of the population that is Muslim (a proxy used only in the female model fit to reflect the notably lower levels of employment recorded among women, primarily in North Africa and the Middle East), and five-year age group, and with random effects on GBD location, region, and super-region. We then aggregated results within every location-year to calculate the employed population ages 15-69 as a proportion of the total population, using GBD 2019 estimates of age-specific populations.

Due to the small size of specific health worker cadres relative to the total employed population – and the instability that consequently resulted from data transformations in the modelling process – we removed cadre-specific values of zero and modelled the remaining proportion data in log space as the number of workers per 10 000 employed population. This greatly increased the stability of these models. Since all health workers combined constituted a larger proportion of the total employed population, it was not necessary to adjust these data in any way, and it was modelled directly as a proportion in logit space. To control for unrealistic trends due to stochastic variation in smaller cadre models and to ensure consistency across modelled results, estimates for all health workers in the aggregate were used as a more trustworthy envelope to which cadre-specific estimates were raked at the draw level. Raking here refers to the application of a rescale factor to all cadre-specific estimates to ensure that they summed to the envelope category of all health workers. Finally, we converted raked estimates of health worker cadres from proportions of employed populations ages 15-69 to proportions of total populations, using the output draws from the employment ratio model. 1000 draws of final estimates were summarised using the mean and the 2.5th and 97.5th percentile as the 95% uncertainty interval.

Table 8. Covariates for ST-GPR models by cadre

Model	Covariates
All health workers	Socio-demographic Index (SDI)<br>Professional workforce share<br>Total Health Expenditure (THE) per capita
Physicians	Socio-demographic Index (SDI)<br>Professional workforce share
Nursing personnel	Socio-demographic Index (SDI)<br>Professional workforce share
Dentists	Socio-demographic Index (SDI)<br>Professional workforce share
Dental assistants	Socio-demographic Index (SDI)
Pharmacists	Socio-demographic Index (SDI)<br>Professional workforce share
Pharmaceutical technicians and assistants	Socio-demographic Index (SDI)<br>Professional workforce share

Section 2. Estimating the relationship between health worker densities and universal health coverage

2.1 Previous thresholds

In the 2006 World Health Report, WHO identified 22.8 physicians, nurses, or midwives per 10 000 population as a minimum health workforce threshold.[11] This figure was based on the average number of health workers observed in countries achieving skilled birth attendance above 80%.[12] In 2016, WHO revised this number, suggesting that 44.5 physicians, nurses, or midwives per 10 000 population were needed.[13] This second threshold was based on skilled health worker densities associated with global median achievement on an index composed of 12 SDG indicators. Although these threshold estimates have received widespread attention, particularly in relation to the Millennium

Development Goals and later the Sustainable Development Goals, they have several shortcomings. First, these thresholds are based on datasets that were not produced using standardised methods, but rather compiled predominantly from government reports that used varying data-collection methods and cadre definitions. WHO researchers engage in triangulation efforts to standardise these heterogeneous inputs to available censuses and other gold-standards, though WHO HRH datasets still exhibit discrepancies between sources of different types for the same location in similar years. Second, WHO thresholds identify minimum health worker levels in the aggregate, rather than providing cadre-specific health worker thresholds. In other words, the WHO thresholds do not identify needs for specific cadres and imply a 1:1 substitutability between cadres within their aggregate threshold. Third, WHO thresholds rely on a limited measure of health-care performance. The 12 SDG indicators used to fit the WHO threshold all reflect crude coverage rather than effective coverage, and therefore do not incorporate the quality or effectiveness of the services provided. They are also heavily skewed toward interventions pertaining to maternal, neonatal, and communicable conditions rather than non-communicable conditions, for which disease burdens are on the rise in most parts of the world.[7] Two of the indicators also pertain to risk factors, such as the prevalence of tobacco smoking, which largely fall outside of the direct influence of health system interventions. For these reasons, the WHO thresholds may not accurately capture the broad range of health-care services on which HRH levels are likely to have an impact.

2.2 Universal health coverage (UHC) effective coverage index

We measured health system performance using a universal health coverage (UHC) effective coverage index developed during the GBD 2019 estimation cycle. The index leverages a variety of estimates related to disease burden and intervention coverage from GBD 2019. It was intended to improve upon the UHC service coverage index proposed in the GBD 2017 round,[2] and followed measurement guidance from consultations with the WHO General Programme of Work 13 Expert Reference Group. Further details on the construction of the GBD 2019 effective coverage index are available elsewhere.[3]

The UHC effective coverage index quantifies on a scale of 0 to 100 the availability, use, and quality of essential health services for a number of service areas (promotion, prevention, and treatment) applicable to a range of ages across the lifespan. The index is composed of 23 tracer indicators, four of which directly measure intervention coverage and 19 of which, in the absence of reliable effective coverage data, are indirect proxies for access to quality care based on disease outcomes. The four coverage indicators encompass met need for modern contraception, DTP3 immunisations, MCV1 immunisations, and ART coverage. The 19 outcome-based indicators are related to antenatal, peripartum, and postnatal care for mothers and neonates and treatment for the following conditions: lower respiratory infections, diarrhoea, tuberculosis, leukaemia, asthma, epilepsy, appendicitis, paralytic ileus and intestinal obstruction, diabetes, ischaemic heart disease, stroke, chronic kidney disease, chronic obstructive pulmonary disease, cervical cancer, breast cancer, uterine cancer, and colon/rectum cancer. These indicators were each associated with one or more age groups to which existing health system interventions are applicable. The following age groups were used: reproductive and newborn age spans, children under age 5 years, children and adolescents ages 5-19 years, adults ages 20-64 years, and older adults at least 65 years of age. For every location, year, and age group, each indicator was scored using estimates pertaining to the corresponding population (eg, one score for chronic kidney disease treatment among adults ages 20-64 in Thailand in 2005).

Scores for the four health service coverage indicators came directly from estimates of coverage percentages. For the 19 outcome-based proxy measures, scores came from mortality-to-incidence ratios, mortality-to-prevalence ratios (for longer-term conditions), risk-standardised deaths, or other mortality measures like maternal mortality ratios, all of which strive to capture the degree to which health system activities successfully prevent adverse outcomes. These proxy measures were scaled from 0 to 100 within each age group using the 2.5th and 97.5th percentiles estimated across all locations and years.

A weighted average of scores was used to estimate a single index value for every location and year from a variety of indicators and age groups. Weights for these indicators varied across contexts based on the intervention's importance in contributing to health gains. The rationale for weighting indicators is that the meaning of health-service coverage will vary by location due to variations in local disease burdens. Health gain weights were calculated using estimates of what the disease burden would have been if there had been full health-service coverage and what it would have been if there had been no health-service coverage. Disease burdens were measured in disability-adjusted life-years (DALYs).

DALY burdens for the two counterfactual scenarios of zero and full intervention coverage were calculated using estimates of intervention coverage, observed DALYs associated with a disease or condition, and external assessments of intervention effectiveness. Interventions were assigned to ordinal categories with associated effectiveness percentages based on the findings of published literature reviews in order to facilitate these calculations. The difference in DALYs between the zero and full service coverage scenarios constituted the potential health gain of the intervention for a given location, year, and age group. Dividing a potential health gain by the sum of all potential health gains for a given location and year resulted in a fraction representing the health gain weight for that combination of indicator, age group, location, and year. To prevent any specific indicator from dominating the index, health gain weights were grouped into terciles for every location-year, and only the tercile's average health gain weight was used for all associated combinations of intervention and age group. For a given location-year, the weighted average of all indicators and age groups using these health gain weights yielded the overall index value for UHC effective coverage.

Uncertainty was propagated throughout the UHC index estimation using 1000 draws of each measure. Contributions to uncertainty in final index values included uncertainty in modelled estimates of coverage and mortality-based proxies as well as uncertainty in estimated health gain weights. The mean and variance of the draws of the overall UHC effective coverage index for all 204 locations from 1990 to 2019 were then used in this study's SFM threshold analysis.

For this study's estimation of minimum health worker density thresholds, we aimed to generate cadre-specific targets rather than one aggregate value. To this end, we used the four health worker cadre groups identified in the Sustainable Development Goals (SDGs) indicator 3.c.1: physicians; nurses and midwives; dentists and dental assistants (dentistry personnel); and pharmacists and pharmaceutical assistants (pharmaceutical personnel). Since some of the modelled cadres in this study are more detailed than these four groups, there are actually six cadre-specific models that contribute to estimates for the groups: one for physicians, one for nurses and midwives, two for dentistry personnel, and two for pharmaceutical personnel.

While aggregating estimates into these groups precludes the identification of distinct thresholds for sub-categories of each type of health worker, it also controls for potential substitution effects between highly related occupations and accounts for variation in the way that different locations and sources may have operationalised ISCO guidelines for very similar positions. Beyond these aggregations, this analysis does not account for substitution effects between cadres. This means that if a location driving the frontier in physicians, for instance, can only maintain its level of UHC by compensating with very high levels of nurses and midwives, then the physician threshold estimated from such a frontier would not account for that, and thus might be too low for countries with fewer nurses and midwives. The substitution phenomenon is particularly relevant in the case of task-shifting cadres, such as nurse practitioners, where there is notable overlap in roles between cadres. Because this study was unable to estimate task-shifting cadres in isolation, we are unable to comment on the extent to which the proposed thresholds are predicated on the presence of task-shifting in a health system.

Other cadres, though perhaps less similar in their titles than those that were aggregated, likely have the potential for substitution effects with the primary four as well. However, expanding the threshold analysis to include such cadres could have led to substantial misinterpretation of our results. For example, it might be possible for some high-income countries that invest a lot in health system quality and accessibility to achieve high levels of UHC with very few community health workers. If so, the global minimum threshold for community health workers would be very small. Because the thresholds represent minimum necessary levels, this would not be an inaccurate finding. But if we briefly presented such a low threshold without having space to properly discuss its implications, readers might misinterpret this as a statement that community health workers are not valuable or do not serve a critical function in many health systems. That would be an erroneous implication.

Space constraints in the main text also militated against a broader analysis that included more cadres. We could not do justice within such a short article to the range of health workers, their distribution globally, and their corresponding thresholds. Consequently, we have chosen to focus on a more parsimonious set of high-priority cadres highlighted in the SDGs, but do not mean to imply that these are the only HRH cadres with meaningful contributions to national health-care performance.

2.3 Stochastic frontier meta-analysis (SFM)

In order to establish global evidence-based minimum thresholds for health worker densities, we developed a meta-analytic extension for stochastic frontier analysis (SFA). The classic SFA builds a non-deterministic production frontier expressing the maximum output value obtainable from a given input value, based on all observed inputs and outputs provided in a dataset. In this study, datapoints for the stochastic frontier come from modelled results, and therefore have associated estimates of variance (uncertainty). A meta-analytic extension is required to properly account for these variances. We call the modified approach stochastic frontier meta-analysis (SFM). Just as with standard random-effects SFA, the SFM tool models inefficiencies as random effects that must be negative, using a half-normal model. Unlike SFA, SFM accounts for the variances of individual datapoints. In addition, SFM is equipped with three features that make the analysis of the data robust and straightforward. The first is a robust extension that simultaneously detects and removes outliers from the analysis, based on generalised trimming methodology.[14,15] The second is a spline model for the frontier, which does not assume any parametric function and allows the SFM to be applied directly to the data without pre-analysis or post-analysis (no transformations are needed). The third feature is a Bayesian module that permits placing shape constraints and priors on the frontier. Technical details of the SFM approach are described in detail in the attached technical supplement.

We applied the SFM tool using an established universal health coverage (UHC) effective coverage index (based on GBD 2019 results)[3] and its associated variance as the output, and estimates of health worker cadre groups as the input. The four cadre groups included in the frontier analyses were those specified in SDG indicator 3.c.1: physicians, nursing and midwifery personnel, dentistry personnel, and pharmaceutical personnel.[16] We therefore generated four distinct production frontiers, each using all estimates for the cadre group being analysed, for all locations and years. Using all locations and years increased the representativeness and stability of our frontier, as it was fit to a much larger dataset reflecting locations from all parts of the world, and also expanded the range of inputs and outputs for which the frontier could provide estimates. We suspected a priori that frontiers would monotonically increase (the addition of more health workers would not decrease maximum potential UHC achievement) and would exhibit diminishing returns (the contribution of each additional health worker would be more significant where workforce levels are lower). Having observed this suspected relationship in preliminary analyses, we incorporated both as constraints in the frontier fits. SFM analyses were thus run using monotonically increasing concave splines with six knots. These constraints ensured that where data were sparse (for example, at very high levels of HRH), stochastic variations and inefficiencies did not drive the flexible frontier toward unrealistic trends (insinuating, for example, that at certain health workforce densities, additional workers would be detrimental to maximum potential UHC attainment). The right-most segment of the spline was also constrained to be flat in order to preclude extrapolation beyond observed levels of UHC attainment. We used 7.5% trimming, so that the most extreme 7/5% of observations were detected as outliers and excluded as the frontier was constructed. This reduced any subjectivity inherent to ad-hoc outlier identification.

For any cadre-specific health worker density, the corresponding production frontier value provides an estimate of the maximum UHC attainment that is theoretically possible if countries or territories are optimising the efficiency with which they translate human resources into UHC. By comparing actual UHC performance to the estimated maximum potential UHC attainment, our frontier analysis identifies the productive efficiencies with which countries and regions are attaining UHC with their existing health worker densities. To be clear, productive efficiency estimates highlight country capacities related to health system components and contexts *beyond* health workforce densities, such as workforce distribution, the quality of workforce training, or non-workforce factors related to health-care access and quality, such as health-care technology, population distributions, and transportation infrastructure. Accordingly, countries and territories with the same health workforce densities can exhibit a range of productive efficiency scores.

Across the spectrum of health worker densities, countries and territories may be identified as highly efficient, and thus driving the UHC frontier at their level of HRH, or less efficient given what would be expected from the size of their health workforce. For example, a country with a relatively small nurse workforce density may have a UHC performance close to the theoretical maximum, and may therefore have a higher efficiency score than a country that has a larger nurse workforce density and a higher level of UHC yet does not come as close to its theoretical maximum. Countries with low frontier efficiencies may look to more efficient performers with similar health workforce densities for insight into potential improvements in other health system factors that would facilitate better UHC attainment. Therefore, reaching a minimum threshold for health workforce density is by no means sufficient for achieving a target level of UHC effective coverage, and many countries with or without health worker shortages

also have low productive efficiencies, indicating a need for investments beyond just expanding the health workforce size. Since reaching 100% efficiency in the translation of human resources into UHC effective coverage performance is not possible for every location, many countries and territories may require workforce densities beyond the minimum thresholds proposed in this study. However, it is important to note that increasing workforce sizes cannot always compensate for issues underlying low productive efficiency, and certainly may not be a cost-effective or appropriate means of advancing UHC when HRH levels are already sufficient.

In this analysis, we use the fitted production frontiers to identify minimum cadre-specific health worker densities needed to achieve specific UHC attainment targets, assuming countries and territories are operating at maximum productive efficiency. Minimum density thresholds for each health worker cadre can be determined as the x-values of each corresponding frontier as it crosses a given target value of UHC performance. We determined minimum density thresholds for each health worker cadre to achieve performance targets of 80 and 90 out of 100 on the UHC effective coverage index. Since UHC is a measure of essential health services for all populations, every country should strive for the highest attainable score on the corresponding index. However, because SFM is fit to historical data, these production frontiers cannot be used to extrapolate HRH requirements for UHC effective coverage beyond what was achieved between 1990 and 2019. This is one reason why the fitted frontiers never reach the x-axis, because no locations in this analysis achieved UHC effective coverage scores so close to zero. During this study period, the highest levels of estimated UHC effective coverage (at or above 90 out of 100) almost entirely occurred in high-income countries and territories. Given limitations in the number and diversity of locations reaching this UHC effective coverage score, the frontier results for such a target are less likely to be accurate or representative at a global scale. For that reason, we chose to focus on a UHC effective coverage of 80 out of 100 for this study, since it was the highest approximate score that had been reached by a wide range of locations. In future analyses as more countries achieve higher levels of UHC, it may be possible to establish stable and representative thresholds for even higher targets of effective coverage.

It is important to note that choosing a high UHC attainment target should not conflict with the interpretation of thresholds as minimum health workforce densities. Our research aimed to determine the minimum human resources required to achieve a high level of health service coverage and quality. In other words, the findings of this frontier analysis are predicated on a high level of UHC effective coverage (an appropriate health system goal) but provide estimates of the minimum HRH densities needed to meet that target (minimising costs and inefficiency).

It is also important to note that the thresholds proposed in this analysis do not necessarily represent an ideal HRH skill mix for any given location. Health systems around the world differ dramatically in their distribution of HRH cadres for a variety of reasons, and this study does not intend to prescribe exact levels or ratios in the makeup of a health workforce. Instead, this study aims to use existing estimates of HRH to identify a globally applicable common denominator of minimum workforce requirements necessary to meet a specified health system performance goal. This study would suggest, for instance, that health systems more heavily reliant on nurses and midwives should still require the minimum threshold densities for physicians, dentistry personnel, and pharmaceutical personnel in order to reach a UHC level of 80 out of 100. However, in maintaining its own preferred skill mix, that health system would likely find itself surpassing the threshold for nurses and midwives in the process. This example is presented to emphasise that these thresholds only reflect minimum HRH densities, not exact targets. They do not imply that all health systems must employ the same strategies or HRH skill mix in order to achieve high levels of UHC. Future work may explore frontier analyses on subsets of locations that exhibit similar skill mixes to better characterise additional contextual workforce requirements beyond this study's minimum levels. Such tailored thresholds might better characterise what the health workforce would look like in countries achieving high levels of UHC while maintaining a particular skill mix, which could be useful for health system planning purposes.

Section 3. Online tools and glossary of terms

Online tools

GBD 2019 data sources and additional results are presented in a series of tools and dynamic visualisations, available at http://ghdx.healthdata.org/gbd-2019.

Analytic source code for estimates are available at: http://ghdx.healthdata.org/gbd-2019 under the GBD 2019 code for the paper "*Measuring the availability of human resources for health and its relationship to universal health coverage: estimates for 204 countries and territories from 1990 to 2019.*"

List of abbreviations

DAH	development assistance for health
GATHER	Guidelines for Accurate and Transparent Health Estimates Reporting
GBD	Global Burden of Diseases, Injuries, and Risk Factors Study
GHDx	Global Health Data Exchange
GPR	Gaussian process regression
HAQ	Healthcare Access and Quality Index
HRH	human resources for health
ILO	International Labour Organization
ISCO	International Standard Classification of Occupations
MDGs	Millennium Development Goals
MIRs	mortality-to-incidence ratios
NCDs	non-communicable diseases
OECD	Organisation for Economic Co-operation and Development
PAF	population attributable fraction
PCA	principal components analysis
SDGs	Sustainable Development Goals
SDI	Socio-demographic Index
SFA	stochastic frontier analysis
SFM	stochastic frontier meta-analysis
ST-GPR	spatiotemporal Gaussian process regression
UHC	universal health coverage
UI	uncertainty interval
VR	vital registration
WHO	World Health Organization

References

1 Stevens GA, Alkema L, Black RE, *et al.* Guidelines for Accurate and Transparent Health Estimates Reporting: the GATHER statement. *The Lancet* 2016; **388**: e19–23.

2 Lozano R, Fullman N, Abate D, *et al.* Measuring progress from 1990 to 2017 and projecting attainment to 2030 of the health-related Sustainable Development Goals for 195 countries and territories: a systematic analysis for the Global Burden of Disease Study 2017. *The Lancet* 2018; **392**: 2091–138.

3 GBD 2019 Universal Health Coverage Collaborators. Measuring universal health coverage based on an index of effective coverage of health services in 204 countries and territories, 1990–2019: a systematic analysis for the Global Burden of Disease Study 2019. *The Lancet* 2020.

4 Fullman N, Yearwood J, Abay SM, *et al.* Measuring performance on the Healthcare Access and Quality Index for 195 countries and territories and selected subnational locations: a systematic analysis from the Global Burden of Disease Study 2016. *The Lancet* 2018; **391**: 2236–71.

5 World Health Organization. Handbook on monitoring and evaluation of human resources for health. WHO. 2019. https://www.who.int/hrh/resources/handbook/en/ (accessed June 20, 2019).

6 International Labour Organization. International Standard Classification of Occupations: ISCO-08 structure, index correspondence with ISCO-88. 2016. http://www.ilo.org/public/english/bureau/stat/isco/isco08/index.htm (accessed June 20, 2019).

7 Vos T, Lim SS, Abbafati C, et al. Global burden of 369 diseases and injuries in 204 countries and territories, 1990–2019: a systematic analysis for the Global Burden of Disease Study 2019. *The Lancet* 2020; 396: 1204–22.

8 Chang AY, Cowling K, Micah AE, *et al.* Past, present, and future of global health financing: a review of development assistance, government, out-of-pocket, and other private spending on health for 195 countries, 1995–2050. *The Lancet* 2019; **0**. DOI:10.1016/S0140-6736(19)30841-4.

9 GBD 2019 Demographics Collaborators. Global age-sex-specific fertility, mortality, healthy life expectancy (HALE), and population estimates in 204 countries and territories, 1950–2019: a comprehensive demographic analysis for the Global Burden of Disease Study 2019. *The Lancet* 2020.

10 Stanaway JD, Afshin A, Gakidou E, *et al.* Global, regional, and national comparative risk assessment of 84 behavioural, environmental and occupational, and metabolic risks or clusters of risks for 195 countries and territories, 1990–2017: a systematic analysis for the Global Burden of Disease Study 2017. *The Lancet* 2018; **392**: 1923–94.

11 World Health Organization. The World Health Report 2006 - working together for health. WHO. https://www.who.int/whr/2006/en/ (accessed June 20, 2019).

12 WHO. The world health report 2006 - working together for health. Geneva: World Health Organization, 2006.

13 WHO. Health workforce requirements for universal health coverage and the Sustainable Development Goals. Background paper No. 1 to the Global Strategy on Human Resources for Health. *Hum Resour Health Obs* 2016; **17**. http://www.who.int/hrh/resources/health-observer17/en/ (accessed Feb 27, 2019).

14 Yang E, Lozano A, Aravkin A. A general family of trimmed estimators for robust high-dimensional data analysis. *Electron J Stat* 2018; **12**: 3519–3553.

15 Aravkin A, Davis D. Trimmed statistical estimation via variance reduction. *Math Oper Res Publ Online Ahead Print* 2019. DOI:https://doi.org/10.1287/moor.2019.0992.

16 UN Statistics Division. Indicator 3.c.1 - E-handbook on SDG indicators. https://unstats.un.org/wiki/display/SDGeHandbook/Indicator+3.c.1 (accessed Sept 25, 2019).

17 WHO. WHO global health workforce statistics: 2018 update. Geneva: World Health Organization, 2018 http://www.who.int/hrh/statistics/hwfstats/en/ (accessed June 3, 2020).

18 Zheng P, Barber R., Sorensen R. et al. Trimmed constrained mixed effects models: formulations and algorithms. Journal of Computational and Graphical Statistics 2021; 0(00): 1-13.

19 Murray CJL, Aravkin AY, Zheng P, et al. Global burden of 87 risk factors in 204 countries and territories, 1990–2019: a systematic analysis for the Global Burden of Disease Study 2019. The Lancet 2020; 396: 1223–49.

Stochastic Frontier Meta-Analysis

1. Stochastic Frontier Meta-Analysis (SFM)

Stochastic frontier analysis (SFA) [1] is a stochastic analysis of the frontier production function, which expresses the maximum amount of output obtainable from a linear combination of variables of interest. The SFA model we start with is given by

$$y_i = \langle \boldsymbol{x}_i, \boldsymbol{\beta} \rangle - v_i, \tag{1}$$

where y_i are observations, $\langle \boldsymbol{x}_i, \boldsymbol{\beta} \rangle$ is the linear model (linear combination of variables in $\boldsymbol{x}_i$ with weights $\boldsymbol{\beta}$), while v_i is the deviation from the *maximum output*, and so is modeled as a *non-negative* random effect. In the context of Human Resources for Health, the observations y_i are measures of universal health coverage (UHC), and the model $\langle \boldsymbol{x}, \boldsymbol{\beta} \rangle$ is a spline that relates a measure of coverage (e.g. average number of personnel per 10 K population) to UHC.

We develop a meta-analytic extension of SFA, which we call Stochastic Frontier Meta-analysis (SFM). Every observation y_i is subject to random error (computed from aggregated data). We consider the modified model

$$\boldsymbol{y} = \boldsymbol{X}\boldsymbol{\beta}^* - \boldsymbol{v} + \boldsymbol{\epsilon}, \tag{2}$$

with each entry v_i of $\boldsymbol{v}$ a half-normal non-negative random effect with unknown variance η, while each entry ϵ_i of $\boldsymbol{\epsilon}$ is Gaussian $\mathcal{N}(0, \sigma_i^2)$, and represents the reported study-specific error sources with known variances σ_i^2. We introduce three innovations in this method.

- We formulate the explicit likelihood problem for the SFM model, assuming a half-normal model for the non-negative random effects v_i.
- Outliers are a big problem for SFA [1]. We apply the trimmed robust approach [2] in order to automatically identify and remove outliers from each dataset.
- We allow priors and constraints for the SFM model. In particular this lets us incorporate shape constraints on the spline, similar to what was proposed by [8].

The resulting approach lets us model inherently nonlinear relationships through the linear model (2) using splines, remove outliers, and incorporate reported errors across geographic regions. Each of the pieces listed above is now described in detail.

2. SFM: modeling non-negative random effects.

In this section we derive all likelihood formulations for the Stochastic Frontier Meta-analysis (SFM) approach. We use the half-normal model for the random effects v_i:

$$f(v_i|\eta) = \begin{cases} \frac{\sqrt{2}}{\sqrt{\pi\eta}} \exp\left(-\frac{v_i^2}{2\eta}\right) & v_i \geq 0 \\ 0 & v_i < 0. \end{cases}$$

The goal is to estimate $\boldsymbol{\beta}^*$ and η^* from observations. The mixed effects framework provides a natural statistical model which can be used for this inference. The joint distribution of fixed and random effects is then given by

$$\begin{aligned} p(\boldsymbol{\beta}, \eta, \boldsymbol{v}|\boldsymbol{y}) &= p(\boldsymbol{\beta}, \eta, |\boldsymbol{v}, \boldsymbol{y})p(\boldsymbol{v}|\boldsymbol{y}) \\ &\propto \prod_{i=1}^{m} \frac{1}{\sqrt{\sigma_i^2 \eta}} \exp\left(-\frac{(y_i - \langle \boldsymbol{x}_i, \boldsymbol{\beta} \rangle + v_i)^2}{2\sigma_i^2}\right) \exp\left(-\frac{v_i^2}{2\eta}\right) \mathbb{1}_{\mathbb{R}_+}(v_i) \end{aligned} \tag{3}$$

Integrating out the random effects, and taking the negative log of the resulting distribution, we arrive at equivalent maximum likelihood formulation that does not depend on the random effects $\boldsymbol{v}$, but only depends on $\boldsymbol{\beta}$ and η. Define $\widetilde{\Phi}$ to be the complementary error function

$$\widetilde{\Phi}(z) = 1 - \frac{2}{\sqrt{\pi}} \int_0^z \exp(-t^2) dt.$$

Then we have the following closed form likelihood.

$$\begin{aligned} \mathcal{M}(\boldsymbol{\beta}, \eta | \boldsymbol{y}) &= -\ln\left(\int_{\mathbb{R}_+^m} p(\boldsymbol{\beta}, \eta, \boldsymbol{v} | \boldsymbol{y})\, d\boldsymbol{v} \right) \\ &= \sum_{i=1}^m \frac{(y_i - \langle \boldsymbol{x}_i, \boldsymbol{\beta} \rangle)^2}{2(\eta + \sigma_i^2)} + \frac{1}{2} \ln\left(\eta + \sigma_i^2\right) - \ln\left(\widetilde{\Phi}\left(\frac{\sqrt{\eta}(y_i - \langle \boldsymbol{x}_i, \boldsymbol{\beta} \rangle)}{\sqrt{2(\eta + \sigma_i^2)(\sigma_i^2)}} \right) \right) \end{aligned} \tag{4}$$

The SFM approach optimizes these likelihoods to estimate $(\boldsymbol{\beta}, \eta)$.

3. Priors, Constraints, and Splines.

In this section we describe how to set up Bayesian priors, constraints for parameters of interest, and spline models for nonlinear relationships in the SFM setup.

3.1. Priors

The likelihood $\mathcal{M}$ can be updated using prior information. Imposing priors is equivalent to adding penalties to the likelihood function. For the SFM analysis, the only priors we use are those related to the final section of the frontier.

Given a Gaussian prior on $\boldsymbol{\beta} \sim N(\overline{\boldsymbol{\beta}})$, we find the *a posteriori* estimate by solving the problem

$$\min_{\boldsymbol{\beta}, \eta} \mathcal{M}(\boldsymbol{\beta}, \eta) + \frac{1}{2}(\boldsymbol{\beta} - \overline{\boldsymbol{\beta}})^T \boldsymbol{\Sigma}_{\boldsymbol{\beta}}^{-1} (\boldsymbol{\beta} - \overline{\boldsymbol{\beta}}). \tag{5}$$

3.2. Constraints

We allow box constraints and general linear inequality constraints on $(\boldsymbol{\beta}, \eta)$. Taking (5) as a running example, we can impose constraints of the form

$$\begin{aligned} \min_{\boldsymbol{\beta}, \eta} \quad & \mathcal{M}(\boldsymbol{\beta}, \eta) + \frac{1}{2}(\boldsymbol{\beta} - \overline{\boldsymbol{\beta}})^T \boldsymbol{\Sigma}_{\boldsymbol{\beta}}^{-1} (\boldsymbol{\beta} - \overline{\boldsymbol{\beta}}) + \frac{1}{2}(\eta - \overline{\eta})^T \boldsymbol{\Sigma}_{\eta}^{-1} (\eta - \overline{\eta}) \\ \text{such that} \quad & \boldsymbol{l}_f \le \begin{bmatrix} \boldsymbol{\beta} \\ \eta \end{bmatrix} \le \boldsymbol{u}_f, \quad \boldsymbol{C} \begin{bmatrix} \boldsymbol{\beta} \\ \eta \end{bmatrix} \le \boldsymbol{c}, \end{aligned} \tag{6}$$

where $(\boldsymbol{l}_f, \boldsymbol{u}_f)$ are lower and upper bounds on the variables, while $\boldsymbol{C}$ is any matrix. This functionality can be used to impose shape constraints on spline models, including increasing/decreasing, convex/concave, and combinations of these designs.

3.3. Splines

In this section we discuss spline models for dose-response relationships. For general background on splines and spline regression see [5] and [6].

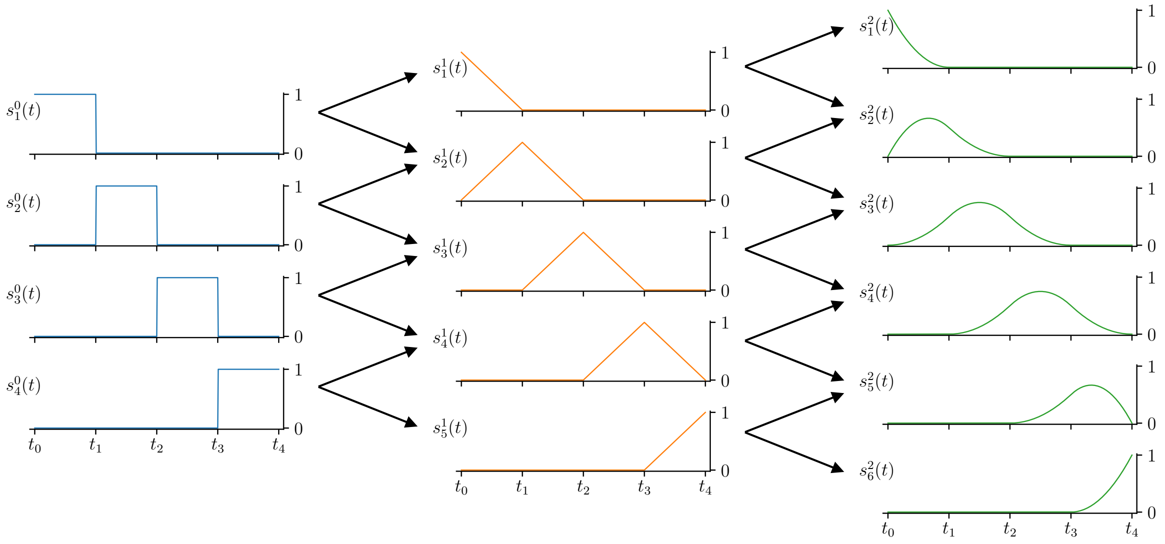

Figure 1. Recursive generation of bspline basis elements (orders 0, 1, 2).

B-splines and bases. A spline basis is a set of piecewise polynomial functions with designated degree and domain. If we denote polynomial order by p, and the number of knots by k, we need $p+k$ basis elements s_j^p, which can be generated recursively as illustrated in Figure 1.

Given such a basis, we can represent any dose-response relationship as the linear combination of the spline basis elements, with coefficients $\boldsymbol{\beta} \in \mathbb{R}^{p+k}$:

$$f(t) = \sum_{j=1}^{p+k} \beta_j^p s_j^p(t). \tag{7}$$

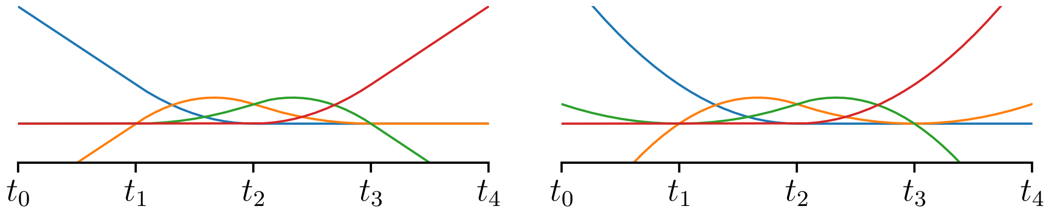

Figure 2. Spline extrapolation. Left: linear extrapolation. Right: nonlinear extrapolation.

An explicit representation of (7) is obtained by building a design matrix $\mathbf{X}$. Given a set of t values at which we have data, the jth column of $\mathbf{X}$ is given by the expression

$$\mathbf{X}_{\cdot,j} = \begin{bmatrix} s_j^p(t_0) \\ \vdots \\ s_j^p(t_k) \end{bmatrix}.$$

The model for direct observations data coming from the spline (7) can now be written compactly as

$$\boldsymbol{y} = \mathbf{X}\boldsymbol{\beta} + \boldsymbol{v} + \boldsymbol{\epsilon}_i,$$

and has the same form as (1).

Enforcing linear tails. For the frontier analysis, we need to ensure that the last segment of the spline does not go above a theoretical limit, typically set at 1. To do this, we allow an option to make the last segment linear. The prior capabilities can then be used to set a prior for the slope of this segment to be 0 (i.e. flat). The estimated spline is then a best fit to the data, subject to this specification.

In general, using linear head and/or tail pieces to extrapolate outside the original domain or interpolate in the data sparse region is far more stable that using higher order polynomials, see Figure 2. The figure shows symmetric linear tail modifications, but for the analyses in the paper we only impose a right linear tail shape constraint.

Shape constraints. We can use constraints to enforce monotonicity, convexity, and concavity. Monotonicity across the domain of interest follows from monotonicity of the spline coefficients. This relationship is derived for particular basis constructions by [5], and has been used in the literature to enforce shape constraints [8]. Current approaches work around the natural inequality constraints by using additional 'exponentiated' variables. Instead we impose these constraints directly as described below.

Focusing just on α, the relationship $\alpha_1 \leq \alpha_2$ can be written as $\alpha_1 - \alpha_2 \leq 0$. Stacking these inequality constraints for each pair (α_i, α_{i+1}) we can write all constraints simultaneously as

$$\underbrace{\begin{bmatrix} 1 & -1 & 0 & \dots & 0 \\ 0 & 1 & -1 & \dots & 0 \\ \ddots & \ddots & \ddots & \ddots & \vdots \\ 0 & \dots & \dots & 1 & -1 \end{bmatrix}}_{\boldsymbol{C}} \begin{bmatrix} \alpha_1 \\ \alpha_2 \\ \alpha_3 \\ \vdots \\ \alpha_n \end{bmatrix} \leq \begin{bmatrix} 0 \\ 0 \\ \vdots \\ 0 \end{bmatrix}.$$

These constraints are directly imposed through the IPOPT interface, along with any lower- and upper-limit constraints on α.

Convexity and Concavity. For any $\mathcal{C}^2$ (twice continuously differentiable) function $f : \mathbb{R} \to \mathbb{R}$, convexity and concavity are captured by the signs of the second derivative. Specifically, f is convex if $f''(t) \geq 0$ is everywhere, an concave if $f''(t) \leq 0$ everywhere. We impose linear inequality constraints on the expressions for $f''(t)$ over each interval. We can therefore easily pick any of the eight shape combinations given in [8, Table 1], as well as imposing any other constraints on α (including bounds).

4. Robust Extension via Trimming

Trimming estimators is a general methodology for robust estimation [10, 2]. For convenience, define

$$\boldsymbol{\theta} = \begin{bmatrix} \boldsymbol{\beta} \\ \eta \end{bmatrix}.$$

Given any likelihood problem of form

$$\min_{\boldsymbol{\theta}} \sum_{i=1}^{m} f_i(\boldsymbol{\theta}) + R(\boldsymbol{\theta}),$$

with f_i is the contribution from the ith datapoint, while $R(\boldsymbol{\theta})$ collects all terms that do not depend on the data, including priors in Section 3.1 and constraints in Section 3.2 [1].

Then the trimmed estimator is formulated as

$$\min_{\boldsymbol{\theta}, \boldsymbol{w}} \sum_{i=1}^{m} w_i f_i(\boldsymbol{\theta}) + R(\boldsymbol{\theta}), \quad 0 \leq w_i \leq 1, \quad \mathbf{1}^T \boldsymbol{w} = h \tag{8}$$

[1] In particular, since R includes constraints, it is infinite-valued off of the feasible region.

where $h \leq m$ is the estimate of inlier datapoints. The set

$$\Delta_h := \left\{\boldsymbol{w} : 0 \leq w_i \leq 1, \mathbf{1}^T\boldsymbol{w} = h\right\}$$

is known as the *capped simplex*, since it is the intersection of the simplex with the unit box [2]. The estimator (8) is compactly written as

$$\min_{\boldsymbol{\theta}, \boldsymbol{w} \in \Delta_h} \sum_{i=1}^{m} w_i f_i(\boldsymbol{\theta}) + R(\boldsymbol{\theta}). \tag{9}$$

5. Optimization

The SFM model is fit using an algorithm based on variable projection [4, 7, 3], which allows us to leverage a third-party solver, IPOPT [9] to optimize over $\boldsymbol{\theta}$, significantly reducing complexity. Consider the joint likelihood (9) and define the value function $v(\boldsymbol{w})$ and values $(\boldsymbol{\theta}(\boldsymbol{w}), \boldsymbol{\gamma}(\boldsymbol{w}))$ by

$$\begin{aligned} v(\boldsymbol{w}) &= \min_{\boldsymbol{\theta}} \sum_{i=1}^{m} w_i f_i(\boldsymbol{\theta}) + R(\boldsymbol{\theta}) \\ \boldsymbol{\theta}(\boldsymbol{w}) &= \arg\min_{\boldsymbol{\theta}} \sum_{i=1}^{m} w_i f_i(\boldsymbol{\theta}) + R(\boldsymbol{\theta}). \end{aligned} \tag{10}$$

We use IPOPT to solve this problem for each $\boldsymbol{w}$, reducing the problem to

$$\min_{\boldsymbol{w} \in \Delta_h} v(\boldsymbol{w}).$$

where $v(\boldsymbol{w})$ is differentiable with derivative given by

$$\nabla v(\boldsymbol{w}) = \begin{bmatrix} f_1(\boldsymbol{\theta}) \\ \vdots \\ f_m(\boldsymbol{\theta}) \end{bmatrix} \tag{11}$$

The top level algorithm is simply a projected gradient method

$$\boldsymbol{w}^+ = \operatorname{proj}_{\Delta_h}(\boldsymbol{w} - \alpha \nabla v(\boldsymbol{w}))$$

for an appropriately chosen stepsize. Each evaluation of ∇v requires a full minimization step over the constrained weighted likelihood with respect to $\boldsymbol{\theta}$ using IPOPT, see (11) and (10). The capped simplex Δ_h is a closed convex set with a simple projection [2]; a simple proximal gradient with line search converges in this case.

6. Estimating Random Effects (Inefficiencies).

Once fixed effects $\boldsymbol{\theta}$ have been estimated, we want to obtain estimates of inefficiency from the joint likelihood (3). We optimize

$$\min_{v_i \geq 0} \frac{(y_i - \langle \boldsymbol{x}_i, \boldsymbol{\beta}\rangle + v_i)^2}{2\sigma_i^2} + \frac{v_i^2}{2\eta} \tag{12}$$

We get the closed form solution

$$\hat{v}_i = \max\left(0, \frac{\frac{1}{\sigma_i^2}(\boldsymbol{x}_i^T\boldsymbol{\beta} - y_i)}{\frac{1}{\sigma_i^2} + \frac{1}{\eta}}\right) = \max\left(0, \frac{\eta(\boldsymbol{x}_i^T\boldsymbol{\beta} - y_i)}{\sigma_i^2 + \eta}\right). \tag{13}$$

References

[1] D. Aigner, C. K. Lovell, and P. Schmidt. Formulation and estimation of stochastic frontier production function models. *Journal of econometrics*, 6(1):21–37, 1977.

[2] A. Aravkin and D. Davis. Trimmed statistical estimation via variance reduction. *Mathematics of Operations Research*, 2019.

[3] A. Y. Aravkin and T. Van Leeuwen. Estimating nuisance parameters in inverse problems. *Inverse Problems*, 28(11):115016, 2012.

[4] B. M. Bell, J. V. Burke, and A. Schumitzky. A relative weighting method for estimating parameters and variances in multiple data sets. *Computational statistics & data analysis*, 22(2):119–135, 1996.

[5] C. De Boor, C. De Boor, E.-U. Mathématicien, C. De Boor, and C. De Boor. *A practical guide to splines*, volume 27. springer-verlag New York, 1978.

[6] J. H. Friedman et al. Multivariate adaptive regression splines. *The annals of statistics*, 19(1):1–67, 1991.

[7] G. Golub and V. Pereyra. Separable nonlinear least squares: the variable projection method and its applications. *Inverse problems*, 19(2):R1, 2003.

[8] N. Pya and S. N. Wood. Shape constrained additive models. *Statistics and Computing*, 25(3):543–559, 2015.

[9] A. Wächter and L. T. Biegler. On the implementation of an interior-point filter line-search algorithm for large-scale nonlinear programming. *Mathematical programming*, 106(1):25–57, 2006.

[10] E. Yang, A. C. Lozano, and A. Aravkin. A general family of trimmed estimators for robust high-dimensional data analysis. *Electronic Journal of Statistics*, 12(2):3519–3553, 2018.
