# Supplementary material for: Measuring the availability of human resources for health and its relationship to universal health coverage for 204 countries and territories from 1990 to 2019: a systematic analysis for the Global Burden of Disease Study 2019
Source: Lancet. 2022 Jun 4;399(10341):2129–54. doi: 10.1016/S0140-6736(22)00532-3 (PMC9168805; doi:10.1016/S0140-6736(22)00532-3)
Supplement: Supplementary appendix 2 [file mmc2.pdf]

THE LANCET

Supplementary appendix 2

This appendix formed part of the original submission and has been peer reviewed. We post it as supplied by the authors.

Supplement to: GBD 2019 Human Resources for Health Collaborators. Measuring the availability of human resources for health and its relationship to universal health coverage for 204 countries and territories from 1990 to 2019: a systematic analysis for the Global Burden of Disease Study 2019. *Lancet* 2022; published online May 23. https://doi.org/10.1016/S0140-6736(22)00532-3.

Supplementary results to Measuring the availability of human resources for health and its relationship to universal health coverage: estimates for 204 countries and territories from 1990 to 2019

These supplementary results contain additional figures and tables.

Table of Contents

List of appendix figures and tables **2**

Appendix Figure 1. Dentistry personnel by SDI quintile and year **3**

Appendix Figure 1. Pharmaceutical personnel by SDI quintile and year **4**

Appendix Figure 2a. Dentistry personnel per 10,000 population, 2019 .... **5**

Appendix Figure 2b. Pharmaceutical personnel per 10,000 population, 2019 .... **6**

Appendix Figure 3a. Physicians per 10,000, 1990 .... **7**

Appendix Figure 3b. Nurses and midwives per 10,000, 1990 .... **8**

Appendix Figure 3c. Dentistry personnel per 10,000, 1990 .... **9**

Appendix Figure 3d. Pharmaceutical personnel per 10,000, 1990 .... **10**

Appendix Table 1. Health worker counts for 16 cadres by GBD super-region, 2019 .... **11**

Appendix Table 2. Health worker shortages for four cadre groups at UHC service coverage of 80 for 204 locations, 2019 .... **12-14**

Appendix Table 3. SDI quintile and UHC effective coverage index by country and territory in 1990, 2000, 2005, 2010, 2015, and 2019 .... **15-18**

List of appendix figures and tables

Appendix Figure 1a. Dentistry personnel by SDI quintile and year
Appendix Figure 1b. Pharmaceutical personnel by SDI quintile and year
Appendix Figure 2a. Dentistry personnel per 10,000 population, 2019
Appendix Figure 2b. Pharmaceutical personnel per 10,000 population, 2019
Appendix Figure 3a. Physicians per 10,000 population, 1990
Appendix Figure 3b. Nurses and midwives per 10 000 population, 1990
Appendix Figure 3c. Dentistry personnel per 10,000 population, 1990
Appendix Figure 3d. Pharmaceutical personnel per 10,000 population, 1990
Appendix Table 1. Health worker counts for 16 cadres by GBD super-region, 2019
Appendix Table 2. Health worker shortages for four cadre groups at UHC service coverage for 80 for 204 locations, 2019
Appendix Table 3. SDI quintile and UHC service coverage index by country and territory in 1990, 2000, 2005, 2010, 2015, and 2019

Appendix Figure 1a. Dentistry personnel by SDI quintile and year

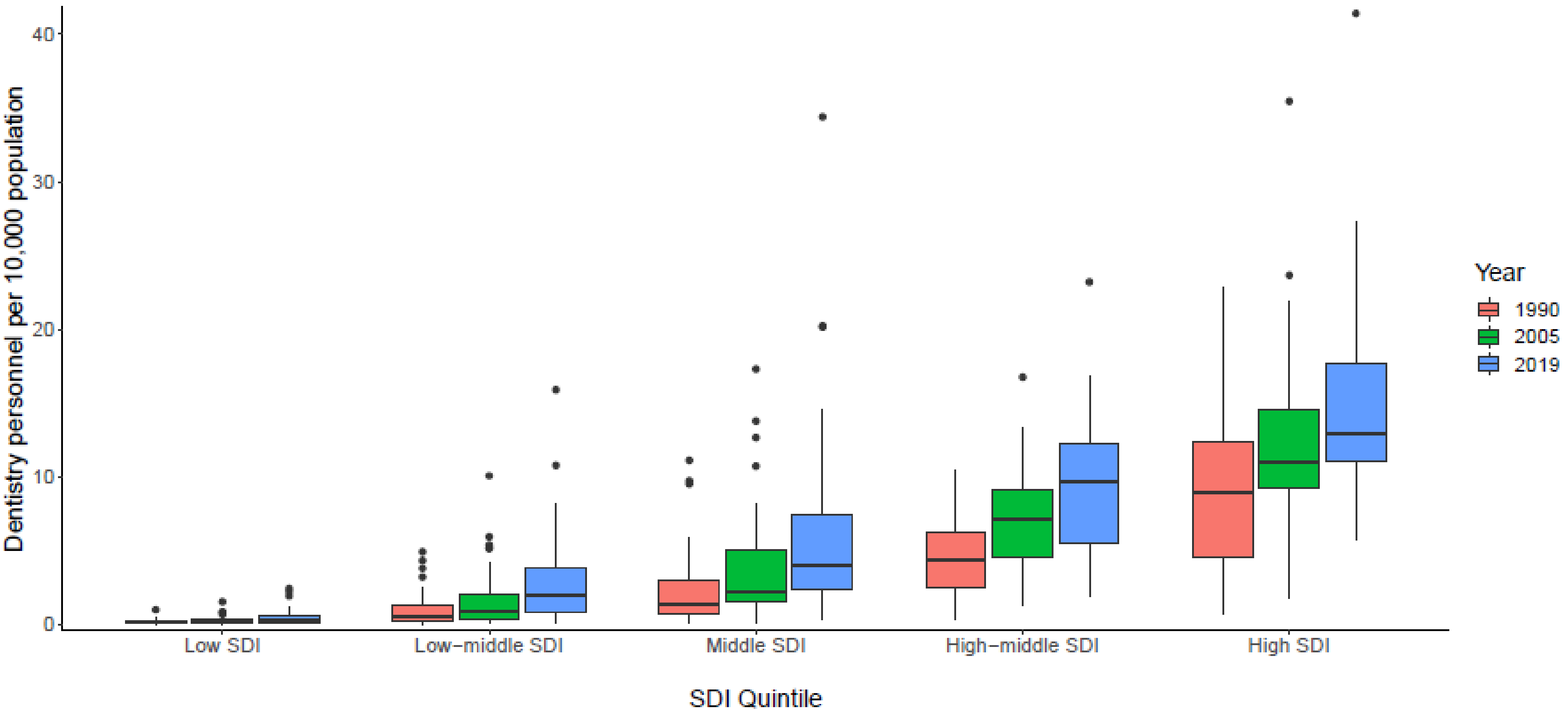

Appendix Figure 1b. Pharmaceutical personnel by SDI quintile and year

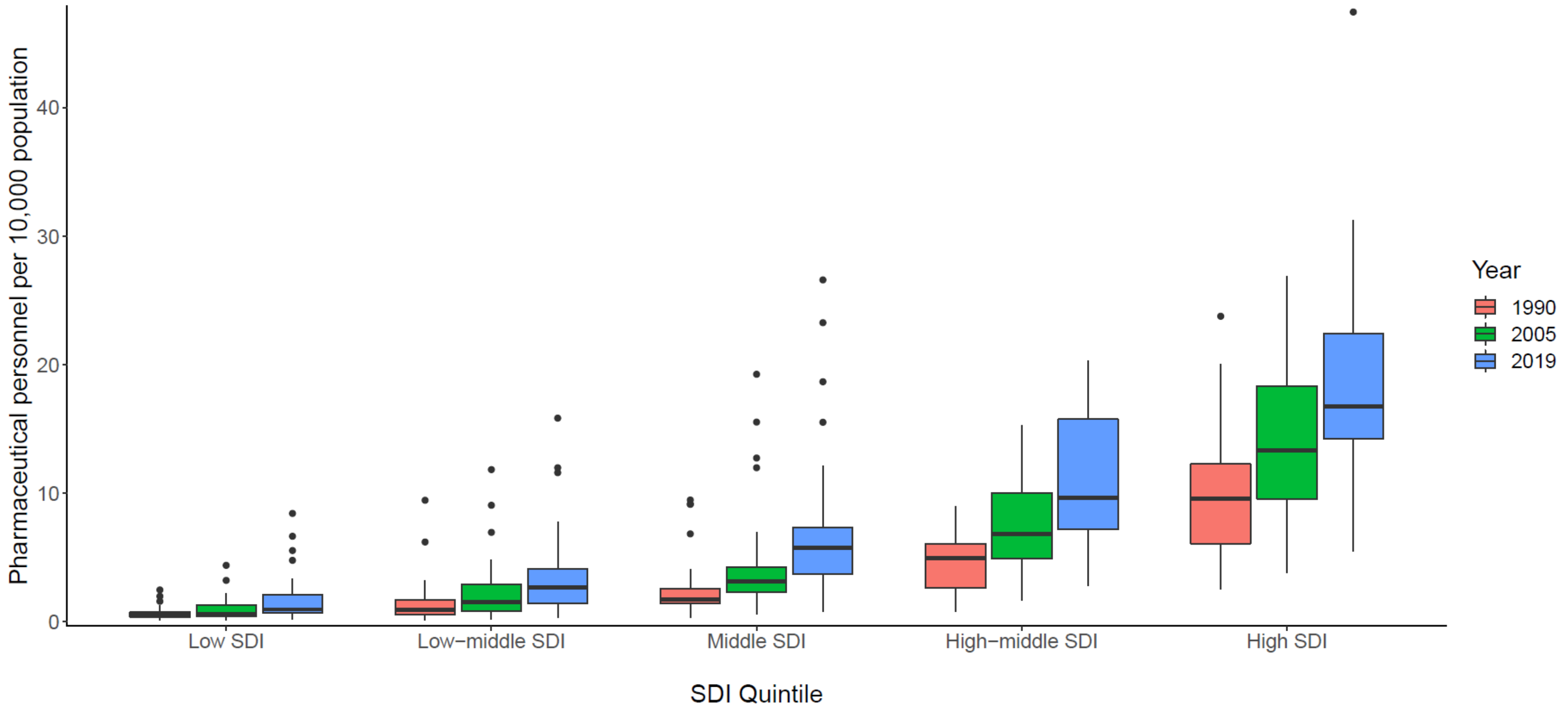

Appendix Figure 2a. Dentistry personnel per 10,000 population, 2019

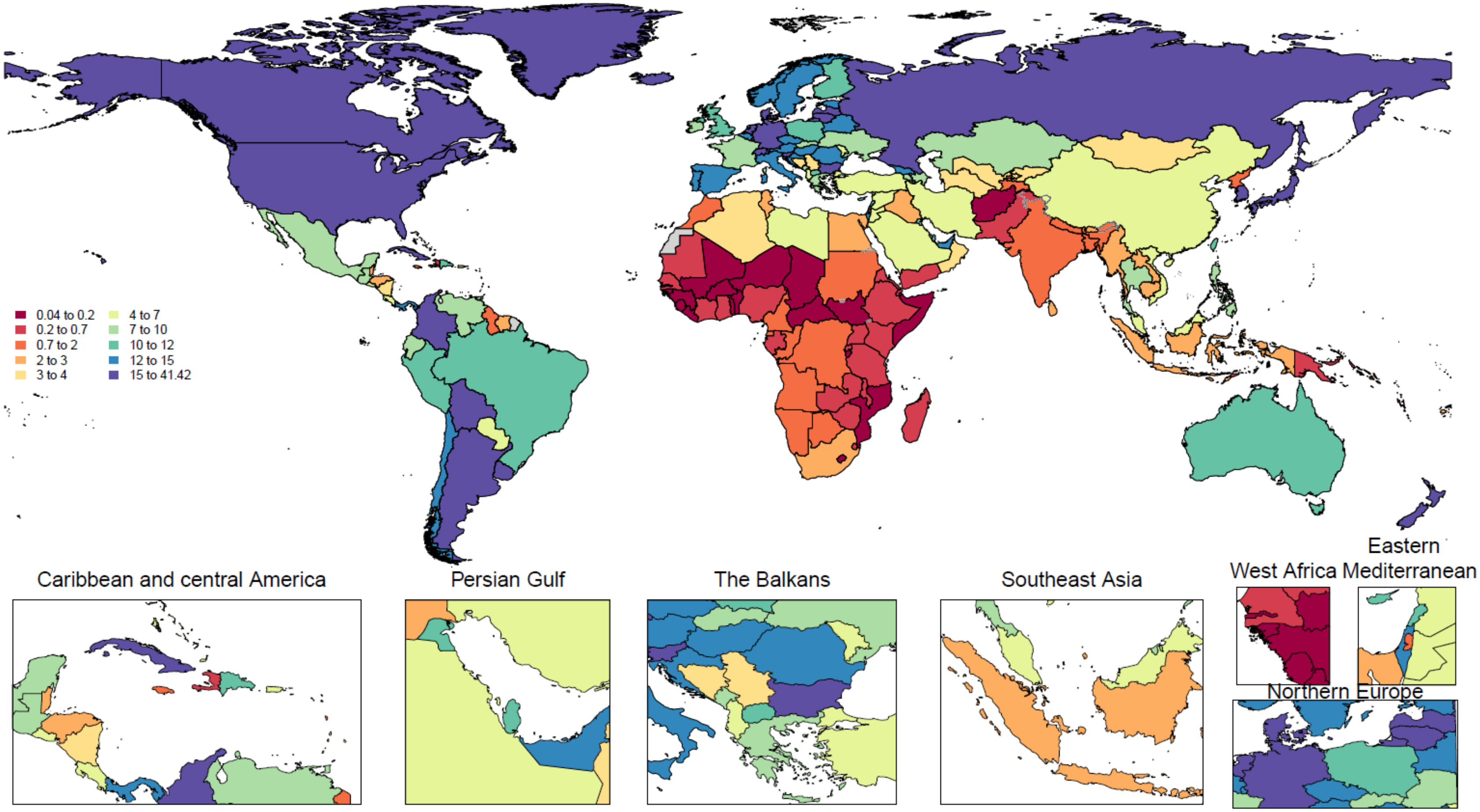

Appendix Figure 2b. Pharmaceutical personnel per 10,000 population, 2019

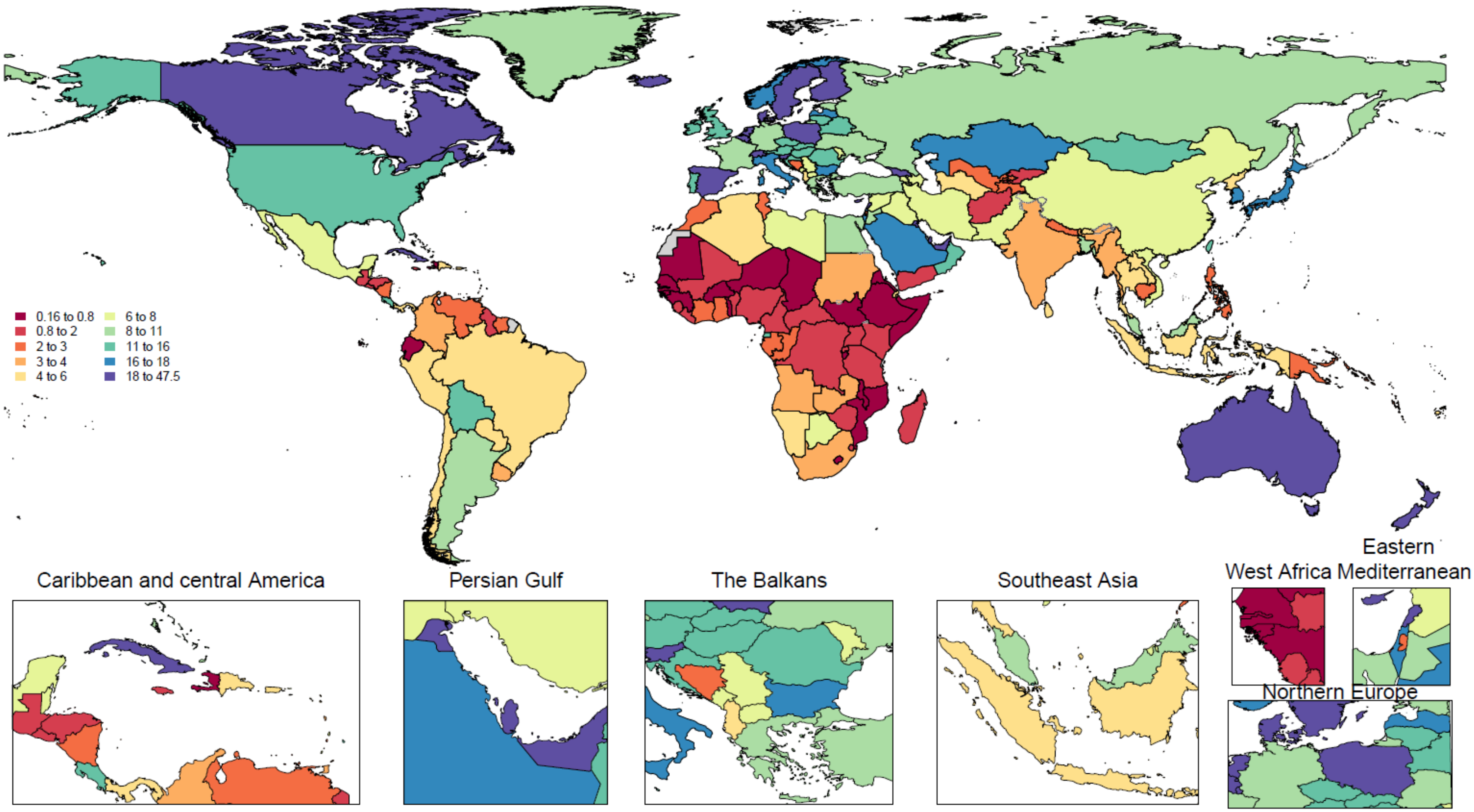

Appendix Figure 3a. Physicians per 10,000 population, 1990

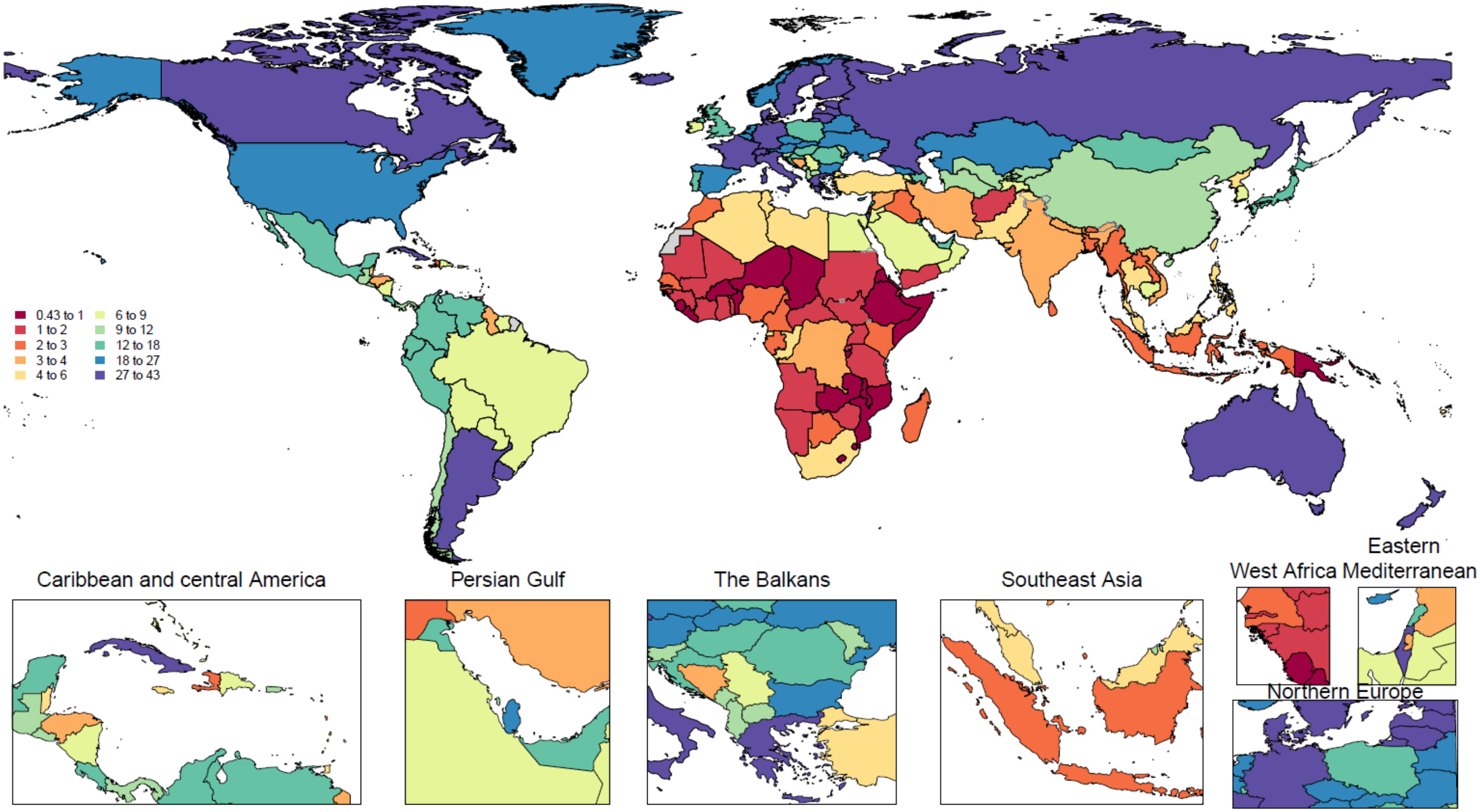

Appendix Figure 3b. Nurses and midwives per 10 000 population, 1990

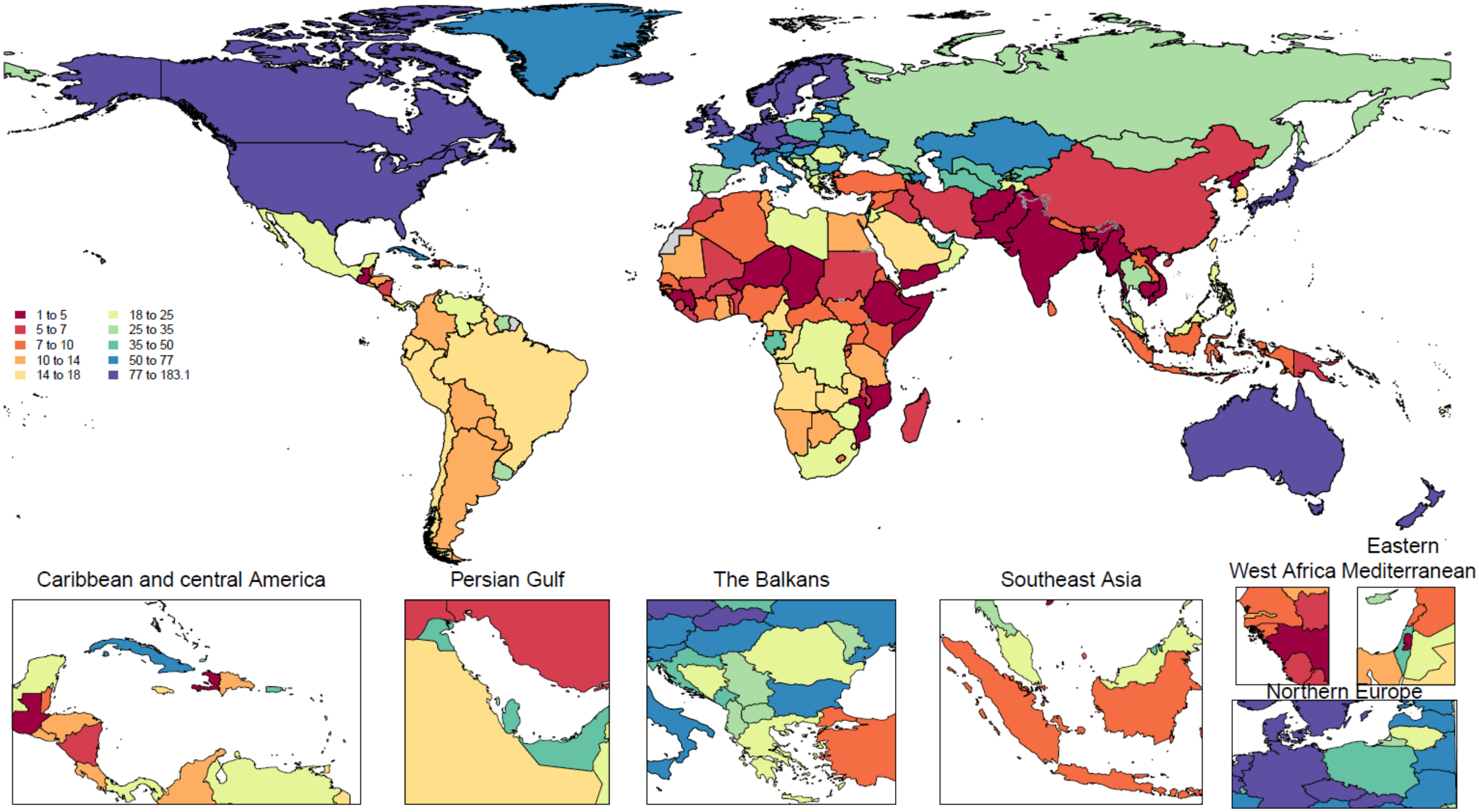

Appendix Figure 3c. Dentistry personnel per 10,000 population, 1990

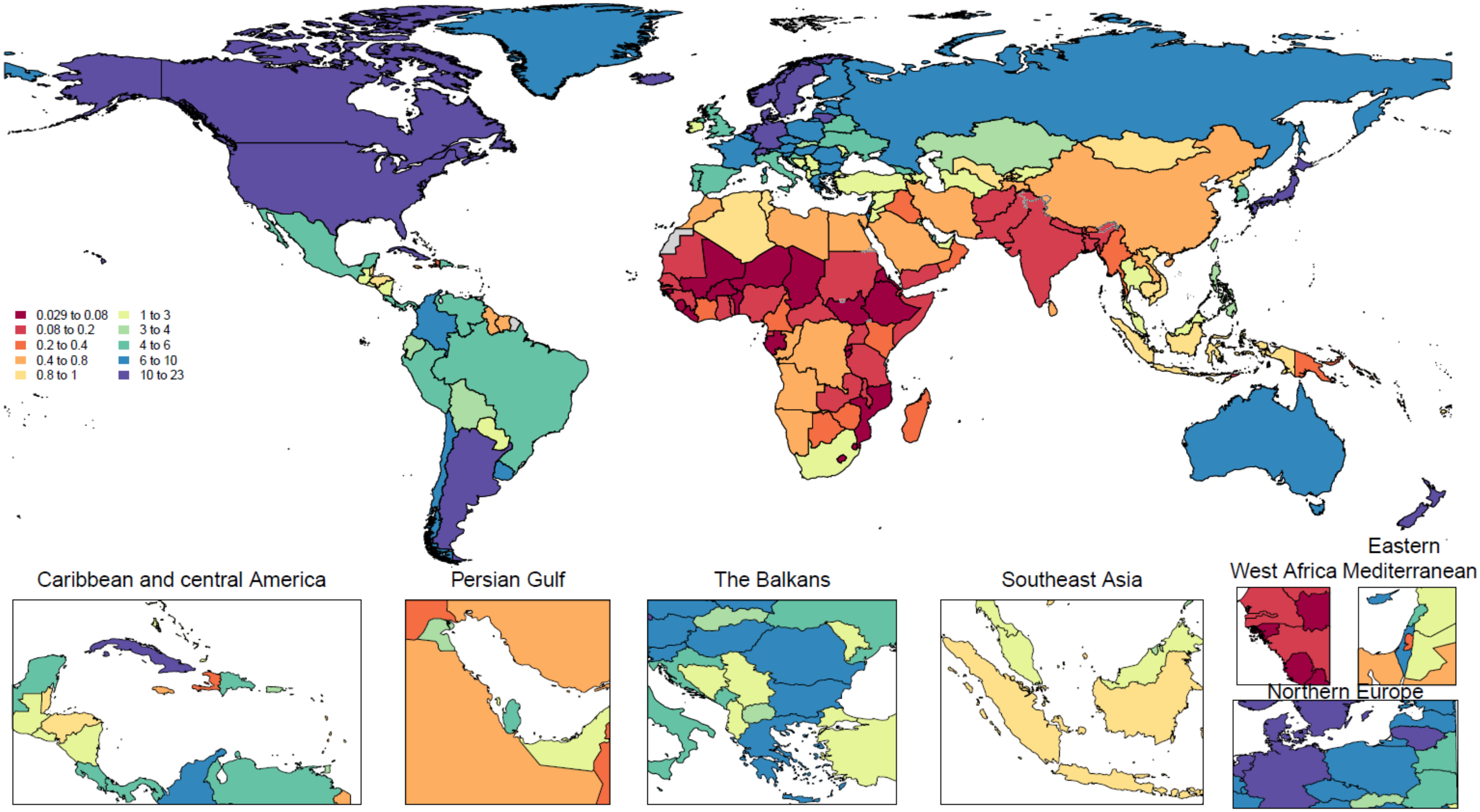

Appendix Figure 3d. Pharmaceutical personnel per 10,000 population, 1990

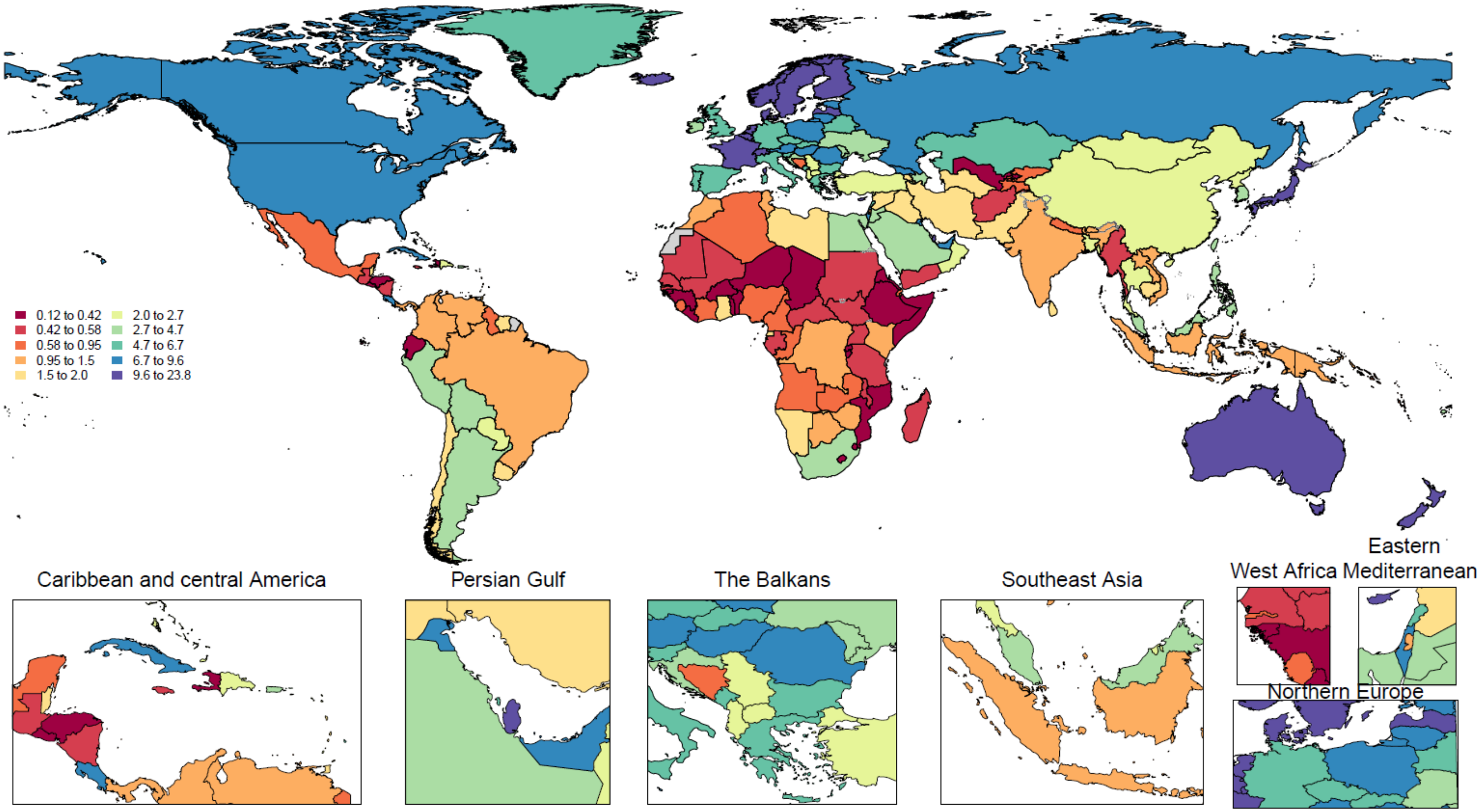

Appendix Table 1. Health worker counts for 16 cadres by GBD super-region, 2019

Appendix table 1. Health worker counts for 16 cadres by GBD super-region, 2019

	Global	Central Europe, Eastern Europe, and Central Asia	High-income	Latin America and Caribbean	North Africa and Middle East	South Asia	Southeast Asia, East Asia, and Oceania	Sub-Saharan Africa
Total population	7,740,000,000	418,000,000	1,080,000,000	584,000,000	609,000,000	1,810,000,000	2,160,000,000	1,080,000,000
Physicians	12,800,00<br>(9,730,000 - 16,600,000)	1,610,000<br>(1,220,000 - 2,070,000)	23,600,000<br>(2,910,000 - 4,440,000)	1,140,000<br>(851,000 - 1,480,000)	664,000<br>(494,000 - 876,000)	1,170,000<br>(865,000 - 1,540,000)	4,340,000<br>(3,160,000 - 5,790,000)	314,000<br>(224,000 - 427,000)
Nurses and midwives	29,800,000<br>23,200,000 - 37,700,000	3,100,000<br>2,350,000 - 4,000,000	12,500,000<br>10,300,000 - 14,900,000	2,580,000<br>2,010,000 - 3,260,000	1,590,000<br>1,200,000 - 2,060,000	1,750,000<br>1,310,000 - 2,300,000	6,410,000<br>4,690,000 - 8,600,000	1,960,000<br>1,460,000 - 2,570,000
Dentistry personnel	4,620,000<br>3,550,000 - 5,970,000	476,000<br>363,000 - 618,000	2,110,000<br>1,710,000 - 2,570,000	607,000<br>454,000 - 792,000	207,000<br>154,000 - 275,000	144,000<br>107,000 - 193,000	1,020,000<br>711,000 - 1,440,000	59,100<br>43,200 - 78,900
Pharmaceutical personnel	5,240,000<br>4,000,000 - 6,700,000	450,000<br>345,000 - 579,000	1,650,000<br>1,340,000 - 2,000,000	305,000<br>230,000 - 396,000	478,000<br>362,000 - 617,000	726,000<br>552,000 - 936,000	1,470,000<br>1,060,000 - 1,950,000	157,000<br>115,000 - 209,000
Healthcare aides, paramedics, and ambulance workers	14,800,000<br>11,300,000 - 19,100,000	1,180,000<br>865,000 - 1,590,000	9,290,000<br>7,370,000 - 11,500,000	1,240,000<br>909,000 - 1,660,000	250,000<br>173,000 - 350,000	692,000<br>500,000 - 935,000	1,680,000<br>1,150,000 - 2,390,000	503,000<br>360,000 - 684,000
Audiologists, speech therapists, and counsellors	3,010,000<br>2,150,000 - 4,100,000	514,000<br>378,000 - 691,000	849,000<br>636,000 - 1,110,000	163,000<br>116,000 - 221,000	20,300<br>14,300 - 28,100	320,000<br>233,000 - 436,000	1,020,000<br>693,000 - 1,440,000	121,000<br>82,700 - 171,000
Clinical officers, medical assistants, and community health workers	4,210,000<br>3,100,000 - 5,590,000	1,010,000<br>754,000 - 1,310,000	1,630,000<br>1,240,000 - 2,100,000	369,000<br>256,000 - 510,000	226,000<br>161,000 - 308,000	443,000<br>325,000 - 600,000	306,000<br>206,000 - 439,000	228,000<br>155,000 - 321,000
Dieticians and nutritionists	1,240,000<br>896,000 - 1,660,000	147,000<br>107,000 - 195,000	595,000<br>442,000 - 780,000	151,000<br>109,000 - 203,000	96,100<br>68,700 - 131,000	18,000<br>12,900 - 24,800	207,000<br>139,000 - 296,000	24,900<br>17,000 - 35,000
Environmental health workers	1,930,000<br>1,390,000 - 2,600,000	209,000<br>151,000 - 280,000	508,000<br>388,000 - 660,000	466,000<br>347,000 - 610,000	147,000<br>103,000 - 205,000	65,200<br>46,800 - 89,900	464,000<br>311,000 - 659,000	69,000<br>47,300 - 97,200
Medical laboratory technicians	4,000,000<br>2,900,000 - 5,410,000	258,000<br>184,000 - 347,000	1,690,000<br>1,280,000 - 2,190,000	324,000<br>231,000 - 441,000	243,000<br>171,000 - 336,000	167,000<br>120,000 - 227,000	1,240,000<br>851,000 - 1,750,000	81,300<br>55,600 - 115,000
Optometrists and opticians	944,000<br>681,000 - 1,280,000	115,000<br>82,500 - 156,000	534,000<br>397,000 - 702,000	74,400<br>50,900 - 106,000	20,900<br>14,100 - 29,500	54,400<br>38,300 - 74,600	113,000<br>74,600 - 160,000	33,600<br>22,800 - 47,800
Home-based personal care workers	11,200,000<br>8,210,000 - 14,900,000	307,000<br>224,000 - 410,000	3,800,000<br>2,940,000 - 4,840,000	206,000<br>145,000 - 286,000	48,800<br>33,700 - 68,200	1,670,000<br>1,240,000 - 2,240,000	4,840,000<br>3,410,000 - 6,660,000	296,000<br>218,000 - 396,000
Psychologists	2,520,000<br>1,840,000 - 3,360,000	301,000<br>223,000 - 399,000	1,170,000<br>888,000 - 1,500,000	348,000<br>253,000 - 464,000	91,500<br>63,700 - 129,000	22,700<br>16,200 - 30,800	523,000<br>354,000 - 743,000	65,400<br>45,700 - 91,100
Medical imaging and therapeutic equipment technicians	3,110,000<br>2,240,000 - 4,220,000	100,000<br>71,900 - 135,000	947,000<br>714,000 - 1,230,000	111,000<br>77,700 - 152,000	102,000<br>72,000 - 140,000	603,000<br>436,000 - 816,000	1,190,000<br>826,000 - 1,660,000	60,900<br>41,900 - 85,500
Physiotherapists and prosthetic technicians	3,390,000<br>2,500,000 - 4,500,000	329,000<br>239,000 - 444,000	2,030,000<br>1,530,000 - 2,630,000	421,000<br>305,000 - 566,000	54,500<br>38,100 - 76,200	63,000<br>45,100 - 85,600	429,000<br>294,000 - 601,000	71,600<br>50,600 - 99,000
Traditional and complementary medicine practitioners	2,230,000<br>1,580,000 - 3,070,000	39,800<br>27,600 - 55,600	485,000<br>359,000 - 637,000	97,200<br>67,600 - 133,000	31,300<br>21,400 - 44,000	226,000<br>168,000 - 298,000	944,000<br>650,000 - 1,350,000	402,000<br>284,000 - 554,000
All health workers	104,000,000<br>(83,500,000 - 128,000,000)	9,990,000<br>(7,990,000 - 12,400,000)	42,500,000<br>(36,000,000 - 49,800,000)	8,470,000<br>(6,800,000 - 10,500,000)	4,130,000<br>(3,210,000 - 5,220,000)	8,370,000<br>(6,580,000 - 10,600,000)	25,900,000<br>(19,600,000 - 33,800,000)	4,330,000<br>(3,340,000 - 5,520,000)

Parentheses reflect 95% uncertainty interval

Appendix Table 2. Health worker shortages for four cadre groups at UHC service coverage of 80 for 204 locations, 2019

Location	Total population	Physicians			Nursing and midwifery personnel			Dentistry personnel			Pharmaceutical Personnel		
		Density per 10,000 population	Shortage per 10,000 population	Absolute shortage (number of workers)	Density per 10,000 population	Shortage per 10,000 population	Absolute shortage (number of workers)	Density per 10,000 population	Shortage per 10,000 population	Absolute shortage (number of workers)	Density per 10,000 population	Shortage per 10,000 population	Absolute shortage (number of workers)
Afghanistan	38,277,536	3.8	20.1	76,900	4.5	64.3	246,263	0.1	7.3	27,865	1.2	13.0	49,602
Angola	30,138,521	4.6	19.3	58,177	16.3	52.5	158,219	1.9	5.5	16,497	3.3	10.9	32,723
Albania	2,720,353	19.0	4.9	1,346	56.2	12.6	3,429	6.0	1.4	379	5.7	8.5	2,308
Andorra	83,064	40.9	-17.0	0	64.3	4.5	37	18.5	-11.1	0	18.9	-4.7	0
United Arab Emirates	9,241,704	30.4	-6.5	0	88.2	-19.4	0	13.3	-5.9	0	47.5	-33.3	0
Argentina	45,115,284	37.5	-13.6	0	23.2	45.6	205,726	16.8	-9.4	0	9.6	4.6	20,613
Armenia	3,019,674	43.6	-19.7	0	83.7	-14.9	0	7.8	-0.4	0	7.3	6.9	2,087
American Samoa	55,505	7.0	16.9	94	50.3	18.5	103	5.5	1.9	10	7.2	7.0	39
Antigua and Barbuda	88,489	22.2	1.7	15	58.5	10.3	91	2.0	5.4	48	4.4	9.8	87
Australia	24,568,113	41.9	-18.0	0	151.6	-82.8	0	11.5	-4.1	0	18.4	-4.2	0
Austria	8,916,185	45.6	-21.7	0	109.8	-41.0	0	13.8	-6.4	0	12.0	2.2	1,923
Azerbaijan	10,278,674	44.6	-20.7	0	117.4	-48.6	0	7.2	0.2	159	7.6	6.6	6,786
Burundi	11,934,361	1.1	22.8	27,199	14.3	54.5	65,022	0.1	7.3	8,766	0.2	14.0	16,701
Belgium	11,419,166	32.7	-8.8	0	134.7	-65.9	0	13.6	-6.2	0	18.9	-4.7	0
Benin	12,665,751	1.3	22.6	28,635	7.8	61.0	77,200	0.1	7.3	9,234	0.8	13.4	16,925
Burkina Faso	22,691,773	0.9	23.0	52,192	11.2	57.6	130,730	0.1	7.3	16,573	0.5	13.7	31,164
Bangladesh	159,259,850	6.5	17.4	276,430	5.0	63.8	1,015,977	0.8	6.6	104,795	8.4	5.8	91,990
Bulgaria	6,934,625	36.8	-12.9	0	70.3	-1.5	0	23.2	-15.8	0	17.3	-3.1	0
Bahrain	1,442,691	17.6	6.3	907	58.4	10.4	1,495	4.3	3.1	447	18.7	-4.5	0
Bahamas	376,940	22.7	1.2	44	57.7	11.1	419	6.1	1.3	50	9.1	5.1	192
Bosnia and Herzegovina	3,299,982	12.0	11.9	3,937	57.7	11.1	3,672	3.9	3.5	1,152	2.8	11.4	3,756
Belarus	9,500,785	43.7	-19.8	0	106.1	-37.3	0	12.3	-4.9	0	11.3	2.9	2,755
Belize	410,094	10.1	13.8	565	23.3	45.5	1,867	2.4	5.0	203	7.7	6.5	268
Bermuda	64,030	30.8	-6.9	0	96.0	-27.2	0	11.5	-4.1	0	11.7	2.5	16
Bolivia (Plurinational State of)	12,011,750	21.3	2.6	3,103	39.5	29.3	35,217	15.9	-8.5	0	11.6	2.6	3,127
Brazil	216,664,814	12.7	11.2	242,900	53.3	15.5	334,930	10.3	-2.9	0	4.8	9.4	203,803
Barbados	297,771	23.1	0.8	23	41.3	27.5	818	5.7	1.7	51	14.1	0.1	3
Brunei Darussalam	437,119	17.2	6.7	293	76.2	-7.4	0	7.9	-0.5	0	6.1	8.1	356
Bhutan	754,250	6.1	17.8	1,344	28.4	40.4	3,044	2.2	5.2	390	4.8	9.4	711
Botswana	2,338,721	5.4	18.5	4,327	46.5	22.3	5,205	1.3	6.1	1,422	6.5	7.7	1,808
Central African Republic	5,299,863	2.5	21.4	11,328	9.4	59.4	31,459	0.2	7.2	3,816	0.9	13.3	7,062
Canada	36,519,840	52.0	-28.1	0	141.5	-72.7	0	41.4	-34.0	0	23.1	-8.9	0
Switzerland	8,775,204	64.2	-40.3	0	163.0	-94.2	0	17.8	-10.4	0	23.5	-9.3	0
Chile	18,198,359	17.5	6.4	11,728	66.2	2.6	4,821	14.8	-7.4	0	5.0	9.2	16,807
China	1,422,350,422	27.2	-3.3	0	31.6	37.2	5,292,332	5.0	2.4	337,745	7.6	6.6	943,509
Cote d'ivoire	26,171,532	3.1	20.8	54,309	13.0	55.8	146,013	0.4	7.0	18,416	2.4	11.8	30,773
Cameroon	29,101,868	3.0	20.9	60,811	26.6	42.2	122,885	0.8	6.6	19,245	1.3	12.9	37,593
Democratic Republic of the Congo	87,670,444	4.1	19.8	173,286	43.8	25.0	219,089	0.9	6.5	57,328	1.7	12.5	109,250
Congo	5,265,846	5.7	18.2	9,574	35.9	32.9	17,349	1.9	5.5	2,880	2.7	11.5	6,062
Cook Islands	17,987	15.0	8.9	16	69.6	-0.8	0	11.1	-3.7	0	6.3	7.9	14
Colombia	47,776,679	25.9	-2.0	0	26.4	42.4	202,765	20.2	-12.8	0	3.5	10.7	51,275
Comoros	714,351	4.6	19.3	1,379	18.3	50.5	3,606	0.8	6.6	469	2.0	12.2	871
Cabo Verde	563,563	9.8	14.1	794	22.9	45.9	2,588	0.3	7.1	397	0.3	13.9	781
Costa Rica	4,716,744	18.7	5.2	2,455	35.8	33.0	15,588	4.4	3.0	1,406	15.5	-1.3	0
Cuba	11,358,510	84.4	-60.5	0	104.6	-35.8	0	34.4	-27.0	0	23.3	-9.1	0
Cyprus	1,313,477	32.1	-8.2	0	64.3	4.5	585	11.7	-4.3	0	20.4	-6.2	0
Czechia	10,643,487	37.3	-13.4	0	116.5	-47.7	0	12.9	-5.5	0	14.9	-0.7	0
Germany	84,914,056	46.9	-23.0	0	176.1	-107.3	0	23.2	-15.8	0	10.1	4.1	34,714
Djibouti	1,202,797	2.8	21.1	2,536	8.7	60.1	7,232	0.4	7.0	847	5.5	8.7	1,043
Dominica	68,681	7.7	16.2	111	56.5	12.3	85	1.9	5.5	38	3.0	11.2	77
Denmark	5,802,733	40.8	-16.9	0	127.0	-58.2	0	20.5	-13.1	0	25.8	-11.6	0
Dominican Republic	10,881,855	22.1	1.8	1,949	19.3	49.5	53,823	10.8	-3.4	0	6.0	8.2	8,934
Algeria	41,847,290	13.8	10.1	42,104	21.8	47.0	196,662	3.1	4.3	17,932	4.0	10.2	42,520
Ecuador	17,588,392	19.6	4.3	7,519	24.0	44.8	78,852	8.1	-0.7	0	0.8	13.4	23,625
Egypt	99,069,551	10.8	13.1	129,915	25.8	43.0	425,572	2.3	5.1	50,264	9.4	4.8	47,872
Eritrea	6,711,213	0.8	23.1	15,517	14.8	54.0	36,243	0.2	7.2	4,831	0.7	13.5	9,061
Spain	46,021,218	46.0	-22.1	0	86.0	-17.2	0	12.1	-4.7	0	18.8	-4.6	0
Estonia	1,312,361	27.9	-4.0	0	66.7	2.1	272	12.1	-4.7	0	9.9	4.3	567
Ethiopia	107,591,164	0.8	23.1	248,859	6.9	61.9	666,523	0.3	7.1	76,601	0.7	13.5	145,141
Finland	5,534,095	32.4	-8.5	0	150.8	-82.0	0	10.1	-2.7	0	18.7	-4.5	0
Fiji	911,248	8.4	15.5	1,411	34.4	34.4	3,139	2.3	5.1	460	5.9	8.3	757
France	66,204,315	24.8	-0.9	0	87.7	-18.9	0	9.0	-1.6	0	10.6	3.6	23,907
Micronesia (Federated States of)	102,116	6.2	17.7	181	18.0	50.8	519	3.7	3.7	38	3.6	10.6	108
Gabon	1,750,038	8.7	15.2	2,659	67.7	1.1	185	0.3	7.1	1,235	2.1	12.1	2,117
United Kingdom	67,220,447	35.1	-11.2	0	130.5	-61.7	0	11.0	-3.6	0	15.6	-1.4	0
Georgia	3,664,752	49.8	-25.9	0	56.9	11.9	4,370	14.5	-7.1	0	18.7	-4.5	0
Ghana	31,536,232	1.7	22.2	70,057	30.8	38.0	119,942	0.4	7.0	22,131	2.9	11.3	35,733
Guinea	12,643,149	1.5	22.4	28,310	5.4	63.4	80,117	0.2	7.2	9,138	0.5	13.7	17,317
Gambia	2,245,866	1.4	22.5	5,063	19.4	49.4	11,088	0.2	7.2	1,620	0.5	13.7	3,076
Guinea-Bissau	1,901,191	2.2	21.7	4,127	13.5	55.3	10,523	0.1	7.3	1,385	0.5	13.7	2,610
Equatorial Guinea	1,419,839	12.7	11.2	1,587	22.8	46.0	6,532	1.2	6.2	874	12.1	2.1	296
Greece	10,337,172	47.8	-23.9	0	49.1	19.7	20,339	9.6	-2.2	0	8.6	5.6	5,761
Grenada	103,215	9.6	14.3	148	53.0	15.8	163	3.0	4.4	46	7.6	6.6	68
Greenland	56,188	26.5	-2.6	0	89.1	-20.3	0	15.4	-8.0	0	9.5	4.7	26
Guatemala	17,776,490	20.6	3.3	5,816	3.5	65.3	116,148	7.1	0.3	604	1.2	13.0	23,196
Guam	170,628	9.9	14.0	239	80.2	-11.4	0	11.2	-3.8	0	14.3	-0.1	0
Guyana	770,705	8.2	15.7	1,212	14.7	54.1	4,172	1.2	6.2	475	1.9	12.3	947
Honduras	9,814,396	8.4	15.5	15,250	18.4	50.4	49,462	2.4	5.0	4,946	1.2	13.0	12,805
Croatia	4,247,902	28.8	-4.9	0	93.1	-24.3	0	14.3	-6.9	0	13.0	1.2	514
Haiti	12,402,099	2.1	21.8	27,094	9.3	59.5	73,748	0.3	7.1	8,845	0.8	13.4	16,637
Hungary	9,674,413	17.0	6.9	6,692	55.7	13.1	12,666	13.8	-6.4	0	11.6	2.6	2,535
Indonesia	259,465,835	7.3	16.6	429,583	24.0	44.8	1,163,097	2.5	4.9	127,816	5.8	8.4	217,195
India	1,390,706,968	6.2	17.7	2,456,438	10.1	58.7	8,164,148	0.8	6.6	916,385	3.1	11.1	1,540,117
Ireland	4,910,357	25.3	-1.4	0	147.7	-78.9	0	9.9	-2.5	0	13.7	0.5	232
Iran (Islamic Republic of)	84,297,882	12.2	11.7	98,470	27.1	41.7	351,394	4.1	3.3	27,573	7.5	6.7	56,300
Iraq	42,119,490	6.3	17.6	74,022	19.1	49.7	209,229	2.3	5.1	21,686	6.3	7.9	33,463
Iceland	344,876	42.2	-18.3	0	173.0	-104.2	0	16.1	-8.7	0	26.5	-12.3	0
Israel	9,309,583	42.3	-18.4	0	62.8	6.0	5,618	14.1	-6.7	0	16.6	-2.4	0
Italy	60,313,170	53.0	-29.1	0	93.0	-24.2	0	13.0	-5.6	0	17.5	-3.3	0
Jamaica	2,810,754	7.9	16.0	4,486	23.7	45.1	12,677	1.4	6.0	1,689	1.2	13.0	3,656
Jordan	11,636,717	13.0	10.9	12,652	38.6	30.2	35,099	5.3	2.1	2,466	11.0	3.2	3,757
Japan	127,788,411	23.5	0.4	5,071	119.2	-50.4	0	22.3	-14.9	0	18.0	-3.8	0
Kazakhstan	18,392,068	42.6	-18.7	0	108.1	-39.3	0	8.0	-0.6	0	16.9	-2.7	0
Kenya	50,227,709	3.3	20.6	103,294	25.4	43.4	217,935	0.5	6.9	34,549	2.0	12.2	61,438

Kyrgyzstan	6,535,459	17.1	6.8	4,450	56.4	12.4	8,115	3.3	4.1	2,710	1.4	12.8	8,343
Cambodia	16,603,118	8.6	15.3	25,482	19.5	49.3	81,789	2.4	5.0	8,231	3.0	11.2	18,650
Kiribati	118,621	2.3	21.6	256	34.9	33.9	402	1.2	6.2	73	1.5	12.7	150
Saint Kitts and Nevis	59,508	21.0	2.9	17	57.0	11.8	70	8.0	-0.6	0	5.2	9.0	54
Republic of Korea	53,398,252	16.2	7.7	41,050	52.6	16.2	86,283	18.1	-10.7	0	17.0	-2.8	0
Kuwait	4,426,561	31.4	-7.5	0	105.5	-36.7	0	11.5	-4.1	0	23.3	-9.1	0
Lao People's Democratic Republic	7,158,250	5.8	18.1	12,953	11.4	57.4	41,071	2.2	5.2	3,709	5.6	8.6	6,190
Lebanon	5,177,069	22.4	1.5	798	22.6	46.2	23,941	11.9	-4.5	0	18.6	-4.4	0
Liberia	4,789,907	1.2	22.7	10,852	13.2	55.6	26,610	0.1	7.3	3,476	1.2	13.0	6,213
Libya	6,735,543	11.1	12.8	8,646	43.6	25.2	16,992	5.0	2.4	1,595	6.6	7.6	5,125
Saint Lucia	174,626	13.1	10.8	189	37.3	31.5	550	4.2	3.2	55	7.0	7.2	126
Sri Lanka	21,854,452	9.3	14.6	31,837	16.1	52.7	115,245	2.0	5.4	11,741	4.2	10.0	21,930
Lesotho	2,091,588	1.0	22.9	4,791	32.8	36.0	7,536	0.2	7.2	1,514	0.6	13.6	2,835
Lithuania	2,794,223	42.6	-18.7	0	26.8	42.0	11,745	27.3	-19.9	0	15.8	-1.6	0
Luxembourg	618,550	34.8	-10.9	0	85.1	-16.3	0	12.7	-5.3	0	21.3	-7.1	0
Latvia	1,915,292	32.4	-8.5	0	66.5	2.3	450	17.7	-10.3	0	16.7	-2.5	0
Morocco	35,952,186	6.6	17.3	62,371	13.5	55.3	198,784	1.5	5.9	21,338	2.6	11.6	41,564
Monaco	37,572	51.2	-27.3	0	165.2	-96.4	0	12.7	-5.3	0	22.4	-8.2	0
Republic of Moldova	3,688,191	24.0	-0.1	0	38.2	30.6	11,282	6.8	0.6	217	6.1	8.1	2,977
Madagascar	26,690,344	4.6	19.3	51,485	8.4	60.4	161,238	0.4	7.0	18,574	1.0	13.2	35,364
Maldives	498,414	34.4	-10.5	0	46.5	22.3	1,112	4.8	2.6	132	15.8	-1.6	0
Mexico	124,940,175	26.6	-2.7	0	57.7	11.1	139,101	9.0	-1.6	0	7.1	7.1	89,219
Marshall Islands	56,842	5.7	18.2	103	33.7	35.1	199	3.0	4.4	25	1.5	12.7	72
North Macedonia	2,152,731	19.3	4.6	993	49.4	19.4	4,167	12.0	-4.6	0	7.9	6.3	1,362
Mali	21,917,467	2.1	21.8	47,752	8.0	60.8	133,181	0.1	7.3	15,967	0.9	13.3	29,173
Malta	439,221	28.2	-4.3	0	105.3	-36.5	0	8.4	-1.0	0	15.8	-1.6	0
Myanmar	54,676,901	9.2	14.7	80,571	11.0	57.8	316,207	2.2	5.2	28,332	3.8	10.4	56,940
Montenegro	620,340	18.6	5.3	326	71.1	-2.3	0	8.3	-0.9	0	6.6	7.6	471
Mongolia	3,387,589	34.4	-10.5	0	47.1	21.7	7,368	3.9	3.5	1,176	12.0	2.2	753
Northern Mariana Islands	42,494	10.7	13.2	56	82.3	-13.5	0	10.7	-3.3	0	12.4	1.8	8
Mozambique	29,528,037	0.8	23.1	68,239	9.4	59.4	175,393	0.2	7.2	21,389	0.7	13.5	39,944
Mauritania	4,014,273	2.4	21.5	8,640	17.3	51.5	20,660	0.3	7.1	2,841	0.6	13.6	5,464
Mauritius	1,276,663	16.5	7.4	939	19.8	49.0	6,259	6.8	0.6	81	26.6	-12.4	0
Malawi	18,442,238	0.9	23.0	42,336	13.0	55.8	102,857	0.3	7.1	13,181	0.8	13.4	24,738
Malaysia	31,301,402	12.7	11.2	34,936	59.0	9.8	30,670	4.8	2.6	8,236	8.3	5.9	18,422
Namibia	2,403,124	5.1	18.8	4,522	24.3	44.5	10,706	1.3	6.1	1,455	5.5	8.7	2,088
Niger	23,295,353	0.9	23.0	53,551	6.3	62.5	145,667	0.0	7.4	17,130	0.2	14.0	32,703
Nigeria	214,823,786	3.3	20.6	442,451	14.6	54.2	1,164,852	0.4	7.0	149,308	1.2	13.0	278,416
Nicaragua	6,510,365	10.2	13.7	8,892	23.5	45.3	29,470	3.4	4.0	2,609	2.0	12.2	7,926
Niue	1,672	13.5	10.4	2	64.5	4.3	1	11.1	-3.7	0	7.7	6.5	1
Netherlands	17,156,788	44.8	-20.9	0	193.8	-125.0	0	17.2	-9.8	0	24.2	-10.0	0
Norway	5,348,847	36.7	-12.8	0	211.0	-142.2	0	12.9	-5.5	0	16.2	-2.0	0
Nepal	30,416,382	6.8	17.1	51,954	27.8	41.0	124,727	1.0	6.4	19,566	2.7	11.5	35,045
Nauru	10,551	8.3	15.6	16	45.8	23.0	24	3.9	3.5	4	7.7	6.5	7
New Zealand	4,495,667	39.6	-15.7	0	155.9	-87.1	0	27.4	-20.0	0	31.2	-17.0	0
Oman	4,583,999	22.2	1.7	759	60.9	7.9	3,640	3.8	3.6	1,658	16.0	-1.8	0
Pakistan	224,062,847	7.8	16.1	360,507	8.3	60.5	1,356,180	0.7	6.7	150,177	6.6	7.6	169,244
Panama	4,160,457	19.5	4.4	1,842	54.0	14.8	6,152	12.8	-5.4	0	4.3	9.9	4,134
Peru	33,995,397	20.5	3.4	11,521	35.3	33.5	114,012	11.1	-3.7	0	5.7	8.5	28,922
Philippines	112,142,764	3.8	20.1	225,797	19.8	49.0	548,959	8.1	-0.7	0	2.9	11.3	126,288
Palau	18,007	15.8	8.1	15	71.5	-2.7	0	9.0	-1.6	0	10.7	3.5	6
Papua New Guinea	9,866,614	1.5	22.4	22,084	6.2	62.6	61,788	0.6	6.8	6,750	2.1	12.1	11,954
Poland	38,434,445	20.2	3.7	14,112	55.8	13.0	50,125	10.7	-3.3	0	20.1	-5.9	0
Puerto Rico	3,521,431	15.3	8.6	3,022	47.3	21.5	7,576	5.8	1.6	579	5.5	8.7	3,069
Democratic People's Republic of I	26,232,861	4.9	19.0	49,787	10.1	58.7	154,095	1.6	5.8	15,281	4.1	10.1	26,488
Portugal	10,651,263	36.3	-12.4	0	74.9	-6.1	0	13.7	-6.3	0	11.6	2.6	2,746
Paraguay	6,930,455	16.8	7.1	4,942	16.3	52.5	36,401	6.9	0.5	325	4.8	9.4	6,484
Palestine	4,956,597	13.5	10.4	5,154	13.1	55.7	27,612	0.8	6.6	3,271	2.4	11.8	5,857
Qatar	2,864,548	33.8	-9.9	0	106.8	-38.0	0	11.0	-3.6	0	29.6	-15.4	0
Romania	19,237,066	17.4	6.5	12,471	44.0	24.8	47,624	14.0	-6.6	0	14.0	0.2	417
Russian Federation	146,717,427	58.4	-34.5	0	52.3	16.5	242,576	15.2	-7.8	0	10.6	3.6	52,894
Rwanda	12,688,117	3.1	20.8	26,374	16.7	52.1	66,042	0.2	7.2	9,076	1.2	13.0	16,485
Saudi Arabia	35,731,972	25.4	-1.5	0	68.8	0.0	0	6.2	1.2	4,346	16.6	-2.4	0
Sudan	40,808,425	3.6	20.3	82,913	14.0	54.8	223,772	1.8	5.6	22,979	3.2	11.0	44,840
Senegal	15,134,067	2.1	21.8	33,044	12.3	56.5	85,572	0.2	7.2	10,894	0.6	13.6	20,571
Singapore	5,667,451	24.3	-0.4	0	77.9	-9.1	0	8.6	-1.2	0	16.0	-1.8	0
Solomon Islands	655,632	2.4	21.5	1,410	23.5	45.3	2,968	1.2	6.2	408	2.6	11.6	758
Sierra Leone	8,284,755	1.9	22.0	18,223	10.4	58.4	48,365	0.1	7.3	6,027	1.3	12.9	10,705
El Salvador	6,256,143	15.7	8.2	5,112	23.3	45.5	28,459	5.1	2.3	1,426	1.0	13.2	8,231
San Marino	33,100	56.0	-32.1	0	132.9	-64.1	0	14.8	-7.4	0	15.3	-1.1	0
Somalia	20,343,112	0.9	23.0	46,839	3.3	65.5	133,167	0.1	7.3	14,832	0.7	13.5	27,502
Serbia	8,746,785	12.6	11.3	9,904	58.1	10.7	9,325	3.9	3.5	3,018	7.5	6.7	5,897
South Sudan	9,282,963	1.6	22.3	20,660	11.4	57.4	53,286	0.0	7.4	6,823	0.8	13.4	12,458
Sao Tome and Principe	205,385	1.2	22.7	467	22.9	45.9	942	0.3	7.1	146	1.1	13.1	270
Suriname	575,888	20.6	3.3	188	55.6	13.2	762	2.6	4.8	275	2.8	11.4	658
Slovakia	5,437,223	32.0	-8.1	0	116.3	-47.5	0	9.1	-1.7	0	11.5	2.7	1,446
Slovenia	2,074,271	25.3	-1.4	0	118.0	-49.2	0	16.7	-9.3	0	20.0	-5.8	0
Sweden	10,222,546	37.9	-14.0	0	148.9	-80.1	0	14.6	-7.2	0	24.2	-10.0	0
Eswatini	1,142,109	1.9	22.0	2,513	38.8	30.0	3,430	0.2	7.2	822	0.8	13.4	1,530
Seychelles	102,145	12.9	11.0	112	42.6	26.2	268	11.9	-4.5	0	20.3	-6.1	0
Syrian Arab Republic	14,491,247	7.2	16.7	24,181	12.5	56.3	81,547	4.5	2.9	4,216	7.3	6.9	10,063
Chad	16,398,860	1.0	22.9	37,588	6.8	62.0	101,743	0.1	7.3	12,031	0.3	13.9	22,825
Togo	7,921,527	1.4	22.5	17,861	13.3	55.5	44,001	0.2	7.2	5,723	0.6	13.6	10,783
Thailand	70,111,586	7.0	16.9	118,205	75.1	-6.3	0	7.1	0.3	2,369	5.7	8.5	59,378
Tajikistan	9,492,414	22.8	1.1	1,084	50.9	17.9	16,988	1.4	6.0	5,685	2.7	11.5	10,934
Tokelau	1,411	6.2	17.7	3	39.9	28.9	4	3.6	3.8	1	4.9	9.3	1
Turkmenistan	5,083,080	30.6	-6.7	0	83.9	-15.1	0	3.9	3.5	1,786	5.8	8.4	4,248
Timor-Leste	1,334,823	5.3	18.6	2,478	8.8	60.0	8,014	0.7	6.7	895	2.9	11.3	1,504
Tonga	102,350	3.9	20.0	205	27.8	41.0	420	2.9	4.5	46	1.7	12.5	128
Trinidad and Tobago	1,387,457	18.7	5.2	725	27.2	41.6	5,766	9.7	-2.3	0	3.8	10.4	1,447
Tunisia	11,571,604	10.1	13.8	15,915	26.1	42.7	49,365	3.0	4.4	5,128	2.6	11.6	13,451
Turkey	81,359,693	9.9	14.0	113,547	23.0	45.8	372,823	4.9	2.5	20,307	8.4	5.8	47,493
Tuvalu	11,798	8.7	15.2	18	34.9	33.9	40	2.0	5.4	6	2.8	11.4	13
Taiwan (Province of China)	23,620,243	9.6	14.3	33,861	55.3	13.5	31,859	10.6	-3.2	0	14.3	-0.1	0
United Republic of Tanzania	56,736,116	4.9	19.0	107,613	22.0	46.8	265,641	0.6	6.8	38,627	1.6	12.6	71,447
Uganda	41,117,856	1.8	22.1	91,009	14.9	53.9	221,521	0.3	7.1	29,272	0.8	13.4	54,997
Ukraine	44,042,431	30.3	-6.4	0	113.5	-44.7	0	9.9	-2.5	0	8.1	6.1	26,892
Uruguay	3,436,137	64.2	-40.3	0	71.9	-3.1	0	20.2	-12.8	0	3.8	10.4	3,575
United States of America	327,978,730	27.2	-3.3	0	123.2	-54.4	0	24.3	-16.9	0	14.2	0.0	0
Uzbekistan	33,677,096	20.7	3.2	10,682	117.6	-48.8	0	3.0	4.4	14,770	2.3	11.9	40,124
Saint Vincent and the Grenadines	113,144	5.2	18.7	212	60.2	8.6	98	1.9	5.5	62	3.0	11.2	127

Venezuela (Bolivarian Republic of	28,068,986	18.8	5.1	14,189	27.9	40.9	114,734	7.1	0.3	770	2.4	11.8	33,073
United States Virgin Islands	103,985	20.7	3.2	33	61.0	7.8	81	7.5	-0.1	0	6.0	8.2	85
Viet Nam	96,372,928	7.7	16.2	156,425	9.9	58.9	567,604	4.3	3.1	29,559	6.7	7.5	71,916
Vanuatu	294,550	2.0	21.9	645	18.3	50.5	1,489	0.9	6.5	192	2.1	12.1	355
Samoa	211,354	3.1	20.8	439	17.0	51.8	1,094	2.3	5.1	107	2.7	11.5	244
Yemen	31,502,896	3.7	20.2	63,512	8.1	60.7	191,375	0.3	7.1	22,265	1.6	12.6	39,715
South Africa	55,588,425	8.3	15.6	86,595	34.6	34.2	190,236	2.5	4.9	27,163	3.9	10.3	57,420
Zambia	18,237,683	1.9	22.0	40,186	21.8	47.0	85,802	0.4	7.0	12,853	3.3	10.9	19,960
Zimbabwe	15,010,852	1.9	22.0	32,986	28.6	40.2	60,392	0.2	7.2	10,791	1.2	13.0	19,565

Shortage per 10 000 column reports the difference between the observed HRH density and that of the minimum threshold. Negative values reflect density in excess of the threshold.

Appendix Table 3. SDI quintile and UHC service coverage index by country and territory in 1990, 2000, 2005, 2010, 2015, and 2019

Location	SDI Quintile	UHC in 1990	UHC in 2000	UHC in 2010	UHC in 2015	UHC in 2019
Japan	High SDI	81 ·1	89 ·7	93 ·8	95 ·9	96 ·5
Iceland	High SDI	78 ·4	86 ·5	94.0	94 ·7	95 ·5
Norway	High SDI	72 ·2	80 ·2	90 ·3	93 ·6	94 ·4
Switzerland	High SDI	78 ·3	84 ·3	90 ·9	92 ·6	93 ·6
San Marino	High SDI	83 ·4	88 ·5	91 ·6	92 ·2	92 ·9
Singapore	High SDI	67 ·3	80 ·2	89 ·7	92.0	92 ·6
Luxembourg	High SDI	66 ·4	78.0	87 ·7	90 ·9	91 ·6
Monaco	High SDI	78 ·5	84 ·6	89.0	90 ·2	91 ·6
Andorra	High SDI	80 ·6	86 ·1	90 ·6	90 ·7	91 ·5
Finland	High SDI	71 ·7	81 ·5	88 ·8	91 ·4	91 ·5
Sweden	High SDI	79.0	83 ·9	88 ·5	89 ·9	90 ·5
Canada	High SDI	80.0	83 ·8	88 ·3	90 ·7	90 ·4
Slovenia	High SDI	67 ·2	72 ·1	85 ·2	88 ·9	90 ·1
Spain	High-middle SDI	65 ·3	76 ·1	88 ·5	90 ·2	90 ·1
France	High SDI	66 ·4	78 ·4	88 ·1	89 ·8	90.0
Netherlands	High SDI	70.0	77 ·2	87 ·7	90 ·2	89 ·8
Australia	High SDI	72 ·4	81 ·8	88 ·5	89 ·3	89 ·6
Republic of Korea	High SDI	59 ·5	75 ·2	85.0	89 ·4	89 ·4
Ireland	High SDI	63 ·4	74 ·6	86 ·5	88 ·7	89 ·2
Italy	High-middle SDI	70 ·6	77 ·9	87 ·1	87 ·9	89 ·2
United Kingdom	High SDI	67 ·9	76 ·5	86 ·2	87 ·7	88 ·1
Belgium	High SDI	67 ·8	75 ·4	83 ·6	87 ·1	87 ·6
Germany	High SDI	66 ·8	77 ·3	83 ·7	85 ·3	86 ·5
Austria	High SDI	71 ·4	79.0	82 ·5	85 ·3	86 ·4
Denmark	High SDI	65 ·5	71 ·1	81.0	84 ·2	84 ·3
Portugal	High-middle SDI	61.0	66 ·4	79 ·3	83 ·6	83 ·7
Malta	High-middle SDI	60 ·1	70 ·6	77 ·8	81.0	83 ·2
New Zealand	High SDI	68 ·3	73 ·6	82 ·4	83 ·3	83 ·2
United States of America	High SDI	73 ·8	75 ·5	81 ·4	82 ·1	82 ·3
Czechia	High SDI	56 ·1	70 ·5	77 ·9	80 ·6	82 ·2
Estonia	High SDI	60 ·2	62 ·6	75 ·1	82 ·1	82 ·1
Kuwait	High SDI	65 ·4	67 ·5	75 ·1	82 ·1	81 ·9
Israel	High-middle SDI	59 ·6	69.0	78 ·8	81 ·3	81 ·6
Qatar	High SDI	60.0	63 ·5	76 ·4	82 ·7	80 ·5
Greece	High-middle SDI	66 ·2	73 ·5	80 ·2	80 ·7	80 ·4
Cyprus	High SDI	57 ·1	61 ·5	74 ·9	78 ·1	79 ·4
Taiwan (Province of China)	High SDI	62 ·9	69 ·4	77 ·5	78 ·7	79 ·3
Costa Rica	Middle SDI	66 ·1	69 ·2	74 ·1	78 ·5	79 ·2
Croatia	High-middle SDI	61 ·2	65.0	73 ·5	76 ·4	79 ·2
Slovakia	High SDI	56 ·4	63 ·4	72 ·5	76 ·2	78 ·2
Bermuda	High SDI	56.0	66 ·6	74.0	76.0	77 ·9
Peru	Middle SDI	52 ·3	61 ·6	65 ·4	72 ·4	75 ·9
Puerto Rico	High SDI	51 ·4	60 ·1	72 ·4	75 ·3	75 ·7
Lebanon	High-middle SDI	54 ·4	66 ·2	69 ·1	72 ·5	74 ·7
Chile	High-middle SDI	56 ·2	65 ·6	68 ·4	72 ·2	74 ·6
Colombia	Middle SDI	55 ·8	58.0	66 ·5	71 ·8	74 ·6
Cuba	Middle SDI	59 ·5	66 ·5	71 ·2	71 ·3	72 ·9
Poland	High-middle SDI	50.0	56 ·7	68 ·5	71 ·6	72 ·9
Hungary	High-middle SDI	53 ·9	63 ·7	67 ·4	70.0	72 ·3
Thailand	Middle SDI	60 ·4	55 ·4	66 ·2	69 ·6	71 ·7
Panama	Middle SDI	54 ·2	59 ·9	64 ·1	67 ·3	71 ·4
Bahrain	High-middle SDI	55.0	58 ·5	66 ·5	72 ·4	70 ·8
Belarus	High-middle SDI	57 ·3	53 ·7	58 ·7	68 ·5	70 ·7
Lithuania	High SDI	60 ·7	64.0	66 ·2	69 ·9	70 ·6
Jordan	High-middle SDI	53.0	57 ·2	68.0	70 ·2	70 ·1
Latvia	High SDI	55 ·5	53 ·3	62 ·2	65 ·4	70 ·1
Albania	Middle SDI	54 ·6	60 ·3	68 ·7	67 ·8	69 ·9
China	Middle SDI	49 ·8	53 ·2	63 ·2	67 ·9	69 ·9
Romania	High-middle SDI	50 ·2	55 ·7	62 ·8	68 ·4	69 ·9
Iran (Islamic Republic of)	Middle SDI	52 ·6	60 ·3	62 ·3	65 ·8	69 ·7
Turkey	High-middle SDI	48.0	57 ·6	64 ·6	66 ·3	69 ·5
Russian Federation	High-middle SDI	54 ·4	47 ·5	57 ·8	64 ·2	69 ·2

Greenland	High-middle SDI	54 ·4	57 ·4	63 ·7	67 ·9	69.0
Oman	High-middle SDI	53 ·7	59 ·8	60 ·5	64 ·4	68 ·7
Uruguay	High-middle SDI	53 ·5	61 ·2	64 ·3	67 ·3	68 ·7
Tunisia	Middle SDI	56.0	60 ·7	63 ·8	67.0	68 ·3
Maldives	Low-middle SDI	37.0	51 ·3	63 ·9	67.0	67.0
Malaysia	High-middle SDI	52 ·1	56 ·1	62 ·2	64.0	66 ·8
Libya	High-middle SDI	57 ·9	63 ·5	66 ·8	67 ·5	66 ·5
Montenegro	High-middle SDI	63 ·5	57 ·8	63 ·7	64.0	66 ·3
Sri Lanka	High-middle SDI	53 ·5	54 ·5	57 ·3	62 ·1	66.0
Brazil	Middle SDI	41 ·6	50 ·9	59 ·2	62 ·9	65 ·7
Brunei Darussalam	High SDI	52 ·5	60 ·7	63 ·2	63 ·5	65 ·7
Algeria	Middle SDI	48 ·6	57 ·6	62 ·6	64 ·8	65.0
Ecuador	Middle SDI	51 ·5	57 ·3	57 ·3	60 ·1	64 ·6
Saudi Arabia	High SDI	50 ·3	53 ·7	58 ·7	62 ·5	64 ·5
Bosnia and Herzegovina	High-middle SDI	54 ·4	62 ·1	61 ·8	62 ·6	64 ·4
Guam	High SDI	63 ·1	68 ·3	67 ·2	63 ·4	63 ·9
Paraguay	Middle SDI	53 ·4	57 ·8	59 ·7	60 ·8	63 ·6
Serbia	High-middle SDI	49 ·3	51 ·4	56 ·9	61 ·8	63 ·6
United Arab Emirates	High SDI	55 ·3	55 ·6	63 ·5	63 ·1	63 ·6
Bulgaria	High-middle SDI	52 ·6	52.0	56 ·5	61 ·3	62 ·8
Armenia	Middle SDI	46 ·9	53 ·2	55 ·9	60 ·4	62 ·7
Cook Islands	High-middle SDI	52 ·9	60 ·3	64 ·7	60 ·5	62 ·4
Republic of Moldova	High-middle SDI	48 ·6	48 ·1	48 ·8	56.0	62 ·3
Cabo Verde	Low-middle SDI	49 ·7	52 ·2	61 ·3	58 ·7	62 ·2
Namibia	Middle SDI	43 ·9	34 ·2	49 ·3	60 ·7	62 ·2
El Salvador	Low-middle SDI	41 ·8	50 ·6	56 ·7	59 ·3	61 ·7
Seychelles	High-middle SDI	54 ·7	56 ·4	60 ·4	60 ·1	61 ·7
Mexico	Middle SDI	46 ·2	53 ·5	58.0	59 ·2	61 ·5
Palestine	Low-middle SDI	47 ·2	51 ·6	56 ·7	60 ·8	61 ·4
Argentina	High-middle SDI	46 ·8	53 ·5	59 ·1	62 ·1	61 ·3
Barbados	High-middle SDI	46 ·8	53 ·2	60 ·9	62 ·3	61 ·3
Venezuela (Bolivarian Republic of)	Low-middle SDI	51 ·2	52 ·6	60 ·5	62 ·3	61 ·1
North Macedonia	High-middle SDI	49 ·6	52 ·8	56.0	59.0	61.0
Bahamas	High-middle SDI	45 ·8	52.0	60.0	60 ·6	60 ·7
Northern Mariana Islands	High-middle SDI	62 ·7	62 ·7	63 ·8	62 ·5	60 ·6
Rwanda	Low SDI	23 ·5	24 ·6	45 ·5	52 ·2	60 ·5
Antigua and Barbuda	High-middle SDI	47 ·3	51 ·9	60.0	57 ·7	59 ·8
Viet Nam	Middle SDI	47 ·6	51 ·5	56 ·8	57 ·1	59 ·8
South Africa	Middle SDI	48 ·5	34 ·9	40 ·6	49 ·7	59 ·7
Kazakhstan	High-middle SDI	44 ·9	38 ·1	50 ·2	56 ·8	59 ·4
Saint Lucia	Middle SDI	40 ·2	50 ·1	58 ·4	59 ·1	59 ·4
Morocco	Low-middle SDI	40 ·8	49.0	53 ·4	53 ·6	58.0
Iraq	Middle SDI	45 ·5	47 ·6	50 ·8	54 ·1	57 ·8
Syrian Arab Republic	Middle SDI	45 ·5	53 ·2	56 ·6	55 ·4	57 ·7
Botswana	Middle SDI	44 ·5	37 ·7	46 ·6	50 ·8	57 ·6
Egypt	Middle SDI	38 ·2	48 ·8	50 ·1	54 ·1	57 ·5
Cambodia	Low-middle SDI	30.0	35 ·3	50 ·1	50 ·8	57 ·1
Jamaica	Middle SDI	44 ·8	47 ·1	57 ·7	58.0	57.0
Nicaragua	Low-middle SDI	47 ·3	51 ·4	51 ·5	54 ·6	56 ·9
Ukraine	High-middle SDI	54 ·2	45 ·3	56 ·6	56 ·4	56 ·9
Georgia	High-middle SDI	44.0	49 ·5	53 ·4	55 ·1	56 ·3
Mauritius	High-middle SDI	44.0	46 ·5	49 ·2	54 ·2	56.0
Malawi	Low SDI	31 ·8	30.0	42 ·1	47 ·9	55 ·8
Trinidad and Tobago	High-middle SDI	37 ·7	39 ·9	52.0	55 ·1	55 ·7
Zimbabwe	Low-middle SDI	44 ·5	34 ·4	34 ·5	42 ·2	55 ·5
United Republic of Tanzania	Low SDI	35 ·3	38 ·5	45 ·3	49 ·6	55 ·4
Philippines	Middle SDI	59 ·7	50 ·9	49 ·2	50 ·4	54 ·8
Sao Tome and Principe	Low-middle SDI	41 ·8	44 ·9	51 ·1	52 ·6	54 ·8
Tokelau	Middle SDI	43 ·4	43 ·7	52 ·6	54 ·9	54 ·7
Belize	Low-middle SDI	39 ·7	37 ·2	52 ·3	54 ·8	54 ·5
Honduras	Low-middle SDI	47.0	44 ·3	52 ·7	52 ·3	54 ·3
Bangladesh	Low-middle SDI	31 ·5	42 ·4	50 ·3	54 ·6	54.0
United States Virgin Islands	High-middle SDI	47 ·8	54 ·1	52 ·7	52 ·4	53 ·9
American Samoa	High-middle SDI	54.0	54 ·6	54 ·2	54 ·4	53 ·4
Eswatini	Low-middle SDI	44 ·6	32 ·1	35 ·8	46 ·8	53 ·4
Mauritania	Low-middle SDI	27.0	38 ·1	46 ·2	48 ·5	53 ·3

Gabon	Middle SDI	35 ·8	35 ·5	42 ·1	49.0	53 ·1
Democratic People's Republic of Kore	Low-middle SDI	46 ·6	47 ·4	49 ·9	50 ·7	53.0
Kyrgyzstan	Low-middle SDI	35.0	32 ·5	42 ·8	48.0	53.0
Saint Kitts and Nevis	High-middle SDI	35 ·9	45 ·7	55 ·6	56.0	53.0
Uganda	Low SDI	32.0	29.0	40 ·1	46 ·1	52 ·8
Zambia	Low-middle SDI	29 ·7	25 ·9	38 ·3	45 ·3	52 ·7
Dominican Republic	Low-middle SDI	37 ·7	45 ·9	48 ·6	47 ·2	52 ·6
Tonga	Middle SDI	54 ·2	50 ·3	51 ·8	52 ·5	52 ·6
Bolivia (Plurinational State of)	Low-middle SDI	29.0	37 ·3	45.0	48 ·3	52 ·4
Guatemala	Low-middle SDI	39 ·4	40 ·2	47 ·5	49 ·2	52 ·3
Dominica	High-middle SDI	44 ·2	49 ·2	51 ·6	50 ·8	51 ·9
Sudan	Low-middle SDI	27 ·5	35 ·2	44 ·5	48 ·9	51 ·9
Kenya	Low-middle SDI	42 ·8	35 ·1	42 ·2	47 ·7	51 ·6
Bhutan	Low-middle SDI	42 ·2	44.0	51 ·9	54 ·4	51 ·4
Niue	High-middle SDI	47 ·9	42.0	48 ·7	49 ·7	51 ·2
Grenada	Middle SDI	34.0	44 ·1	48 ·5	51 ·1	50 ·6
Suriname	Middle SDI	36 ·5	41 ·9	49.0	50 ·7	50 ·3
Equatorial Guinea	Middle SDI	16 ·7	27 ·4	42 ·6	43.0	50 ·1
Burundi	Low SDI	26 ·8	25 ·4	38 ·1	42 ·8	49 ·9
Samoa	Middle SDI	39 ·6	43 ·3	43 ·6	47.0	49 ·9
Saint Vincent and the Grenadines	Middle SDI	37 ·9	43 ·4	49 ·9	47 ·3	49 ·7
Senegal	Low SDI	32.0	38 ·4	45 ·1	45 ·6	49 ·7
Ghana	Low-middle SDI	29 ·4	37 ·4	41 ·8	40 ·6	49 ·3
Yemen	Low SDI	30 ·3	35 ·8	47 ·8	48.0	49 ·1
Indonesia	Middle SDI	38 ·6	43 ·1	47 ·4	49 ·7	48 ·7
Azerbaijan	Middle SDI	32.0	31 ·4	40 ·4	45 ·1	48 ·3
Comoros	Low SDI	35 ·6	36.0	41 ·5	44 ·6	48 ·2
Gambia	Low SDI	47 ·5	44 ·1	46.0	48 ·4	48 ·2
Mongolia	Low-middle SDI	27 ·4	28.0	42 ·4	50 ·5	47 ·9
Nepal	Low SDI	29 ·1	39 ·9	44 ·9	44 ·5	47 ·4
Myanmar	Low-middle SDI	28 ·1	32 ·7	39 ·1	40 ·9	47.0
India	Low-middle SDI	26 ·8	32 ·1	42 ·1	48.0	46 ·9
Liberia	Low SDI	19 ·3	28 ·7	36 ·8	41 ·1	46 ·9
Ethiopia	Low SDI	9 ·8	15 ·5	35 ·8	42 ·5	46 ·5
Tajikistan	Low-middle SDI	41 ·6	36 ·6	40 ·5	42 ·1	46 ·4
Timor-Leste	Low-middle SDI	30 ·7	32 ·1	45 ·7	46 ·5	46.0
Djibouti	Low-middle SDI	38 ·7	33 ·6	40 ·6	42 ·3	45 ·4
Fiji	Middle SDI	44 ·5	38 ·4	42 ·6	43 ·8	45 ·3
Democratic Republic of the Congo	Low SDI	22 ·9	20 ·6	30.0	37.0	45 ·2
Palau	High-middle SDI	46 ·1	45 ·5	49 ·6	48 ·1	45.0
Benin	Low SDI	27 ·3	31 ·3	35 ·7	37 ·9	44 ·7
Marshall Islands	Low-middle SDI	38 ·9	37 ·2	42 ·3	42 ·3	44 ·3
Turkmenistan	Middle SDI	31 ·9	30 ·3	39 ·1	40 ·6	44 ·2
Congo	Low-middle SDI	25.0	23 ·6	35 ·9	39 ·9	44 ·1
Mozambique	Low SDI	24 ·1	31 ·7	31 ·5	36 ·9	44 ·1
Lao People's Democratic Republic	Low-middle SDI	15 ·2	21 ·2	34 ·6	38 ·6	43 ·9
Côte d'Ivoire	Low SDI	26 ·5	26 ·9	33 ·8	37 ·2	43 ·2
Togo	Low SDI	32 ·6	27 ·8	33 ·6	39 ·4	42 ·8
Cameroon	Low-middle SDI	29 ·1	26.0	31.0	35 ·6	42 ·3
Uzbekistan	Middle SDI	38.0	30 ·2	35 ·8	37.0	42 ·3
Eritrea	Low SDI	17 ·5	32 ·5	35 ·1	39 ·2	42 ·2
Sierra Leone	Low SDI	28 ·4	28 ·8	36 ·8	40 ·4	42 ·2
Nauru	Middle SDI	39 ·8	32 ·9	38 ·5	43 ·8	42 ·1
Burkina Faso	Low SDI	22 ·9	25 ·4	37 ·7	40 ·2	41 ·7
Mali	Low SDI	20 ·9	26 ·2	35 ·5	38.0	41 ·5
South Sudan	Low SDI	29 ·8	32 ·4	34 ·4	37 ·3	41 ·5
Guyana	Middle SDI	23 ·4	26 ·2	33 ·4	37 ·1	40 ·7
Madagascar	Low SDI	30 ·5	32 ·8	36 ·2	37 ·2	39 ·7
Tuvalu	Low-middle SDI	29 ·4	33 ·5	39.0	39 ·2	39 ·7
Solomon Islands	Low SDI	25 ·5	25 ·6	33 ·3	30 ·8	39 ·5
Afghanistan	Low SDI	21 ·9	20 ·2	31 ·2	36 ·6	39 ·4
Angola	Low-middle SDI	18 ·7	21 ·6	31 ·5	35 ·5	39 ·3
Pakistan	Low SDI	36 ·2	33 ·3	35 ·1	36 ·8	39 ·2
Lesotho	Low-middle SDI	39 ·1	29 ·8	32 ·1	37 ·8	38 ·8
Nigeria	Low-middle SDI	22 ·8	22 ·1	31 ·7	32 ·8	38 ·3
Papua New Guinea	Low SDI	35 ·6	34 ·1	33.0	36 ·3	37 ·9

Haiti	Low SDI	11 ·9	19 ·4	25 ·1	29 ·3	35 ·9
Kiribati	Low-middle SDI	25 ·8	28 ·5	32 ·1	33 ·5	35 ·9
Guinea-Bissau	Low SDI	18 ·5	20 ·5	23 ·7	29 ·1	35 ·6
Niger	Low SDI	18 ·2	19 ·4	31 ·5	31 ·2	35.0
Micronesia (Federated States of)	Low-middle SDI	34 ·3	34 ·4	38 ·1	36 ·2	34 ·6
Vanuatu	Low-middle SDI	33 ·5	35 ·2	33 ·8	34.0	34 ·3
Guinea	Low SDI	20 ·7	24 ·6	31 ·4	27 ·3	32 ·4
Chad	Low SDI	21.0	18 ·4	26 ·1	28 ·9	31 ·3
Somalia	Low SDI	15 ·2	16 ·3	18 ·8	21 ·6	24.0
Central African Republic	Low SDI	17 ·7	15 ·2	16 ·7	16 ·9	22 ·1

Table in order by 2019 UHC score.
